# Integrated multimodal cell atlas of Alzheimer’s disease

**DOI:** 10.1038/s41593-024-01774-5

**Published:** 2024-10-14

**Authors:** Mariano I. Gabitto, Kyle J. Travaglini, Victoria M. Rachleff, Eitan S. Kaplan, Brian Long, Jeanelle Ariza, Yi Ding, Joseph T. Mahoney, Nick Dee, Jeff Goldy, Erica J. Melief, Anamika Agrawal, Omar Kana, Xingjian Zhen, Samuel T. Barlow, Krissy Brouner, Jazmin Campos, John Campos, Ambrose J. Carr, Tamara Casper, Rushil Chakrabarty, Michael Clark, Jonah Cool, Rachel Dalley, Martin Darvas, Song-Lin Ding, Tim Dolbeare, Tom Egdorf, Luke Esposito, Rebecca Ferrer, Lynn E. Fleckenstein, Rohan Gala, Amanda Gary, Emily Gelfand, Jessica Gloe, Nathan Guilford, Junitta Guzman, Daniel Hirschstein, Windy Ho, Madison Hupp, Tim Jarsky, Nelson Johansen, Brian E. Kalmbach, Lisa M. Keene, Sarah Khawand, Mitchell D. Kilgore, Amanda Kirkland, Michael Kunst, Brian R. Lee, Mckaila Leytze, Christine L. Mac Donald, Jocelin Malone, Zoe Maltzer, Naomi Martin, Rachel McCue, Delissa McMillen, Gonzalo Mena, Emma Meyerdierks, Kelly P. Meyers, Tyler Mollenkopf, Mark Montine, Amber L. Nolan, Julie K. Nyhus, Paul A. Olsen, Maiya Pacleb, Chelsea M. Pagan, Nicholas Peña, Trangthanh Pham, Christina Alice Pom, Nadia Postupna, Christine Rimorin, Augustin Ruiz, Giuseppe A. Saldi, Aimee M. Schantz, Nadiya V. Shapovalova, Staci A. Sorensen, Brian Staats, Matt Sullivan, Susan M. Sunkin, Carol Thompson, Michael Tieu, Jonathan T. Ting, Amy Torkelson, Tracy Tran, Nasmil J. Valera Cuevas, Sarah Walling-Bell, Ming-Qiang Wang, Jack Waters, Angela M. Wilson, Ming Xiao, David Haynor, Nicole M. Gatto, Suman Jayadev, Shoaib Mufti, Lydia Ng, Shubhabrata Mukherjee, Paul K. Crane, Caitlin S. Latimer, Boaz P. Levi, Kimberly A. Smith, Jennie L. Close, Jeremy A. Miller, Rebecca D. Hodge, Eric B. Larson, Thomas J. Grabowski, Michael Hawrylycz, C. Dirk Keene, Ed S. Lein

**Affiliations:** 1 Allen Institute for Brain Science, Seattle, WA USA; 2 Department of Statistics, University of Washington, Seattle, WA USA; 3 Department of Laboratory Medicine and Pathology, University of Washington, Seattle, WA USA; 4 Center for Data-Driven Discovery for Biology, Allen Institute, Seattle, WA USA; 5 Department of Physiology and Biophysics, University of Washington, Seattle, WA USA; 6 Chan Zuckerberg Initiative, Redwood City, CA USA; 7 Kaiser Permanente Washington Health Research Institute, Seattle, WA USA; 8 Department of Neurological Surgery, University of Washington, Seattle, WA USA; 9 Department of Statistics and Data Science, Carnegie Mellon University, Pittsburgh, PA USA; 10 Department of Radiology, University of Washington, Seattle, WA USA; 11 Department of Neurology, University of Washington, Seattle, WA USA; 12 Department of Medicine, University of Washington, Seattle, WA USA

**Keywords:** Gene expression, Epigenomics, Alzheimer's disease, Cellular neuroscience

## Abstract

Alzheimer’s disease (AD) is the leading cause of dementia in older adults. Although AD progression is characterized by stereotyped accumulation of proteinopathies, the affected cellular populations remain understudied. Here we use multiomics, spatial genomics and reference atlases from the BRAIN Initiative to study middle temporal gyrus cell types in 84 donors with varying AD pathologies. This cohort includes 33 male donors and 51 female donors, with an average age at time of death of 88 years. We used quantitative neuropathology to place donors along a disease pseudoprogression score. Pseudoprogression analysis revealed two disease phases: an early phase with a slow increase in pathology, presence of inflammatory microglia, reactive astrocytes, loss of somatostatin^+^ inhibitory neurons, and a remyelination response by oligodendrocyte precursor cells; and a later phase with exponential increase in pathology, loss of excitatory neurons and Pvalb^+^ and Vip^+^ inhibitory neuron subtypes. These findings were replicated in other major AD studies.

## Abstract (web summary)

The affected cellular populations during Alzheimer’s disease progression remain understudied. Here the authors use a cohort of 84 donors, quantitative neuropathology and multimodal datasets from the BRAIN Initiative. Their pseudoprogression analysis revealed two disease phases.

## Main

Alzheimer’s disease (AD) is characterized by deposition of hallmark pathological peptides and neurodegeneration that progress across partially overlapping neuroanatomical and temporal axes^1,2^. This process is generally believed to follow a stereotyped progression with amyloid beta (Aβ) plaques starting in the cerebral cortex^3^ and hyperphosphorylated Tau (pTau) aggregation (neurofibrillary tangles (NFTs)) starting in the brainstem and limbic system^4^. Single-cell and spatial genomics technologies now offer a dramatically higher-resolution analysis of complex brain tissues; multiple studies have now begun to apply them to identify cellular vulnerabilities and molecular changes with AD^5–15^.

Recent work catalyzed by the BRAIN Initiative Cell Census Network (BICCN) and BRAIN Initiative Cell Atlas Network (BICAN) has established best practices in experimental and quantitative analyses to harness single-cell genomics, spatial transcriptomics and patch sequencing (patch-seq) methods to characterize cellular properties and build a knowledge base of brain cell types^16–22^. Systematic BICCN and BICAN efforts are now producing the first brain-wide cell atlases of the mouse^23^ and human brain^17–19^, providing robust and highly curated, genomically based reference cell classifications, spatial maps of cellular distributions, and characterization of cellular properties in the normal brain. These reference classifications provide an extremely powerful foundational reference to understand the cellular, molecular and epigenomic underpinnings of AD. Furthermore, mapping to this reference allows integration across data modalities and across independent studies to validate findings and leverage a growing knowledge base on the properties and function of cell types that are affected in disease.

The Seattle Alzheimer’s Disease Brain Cell Atlas (SEA-AD) consortium aims to use these advances to produce the highest-resolution, multimodal, brain-wide cell atlas of AD and related dementias mapped to the BICCN foundational references. Keys to achieving this goal are: (1) a high-quality donor cohort spanning the full spectrum of AD pathology (instead of a case-control design), recruited from longitudinal cohort studies with well-characterized participants; (2) the use of improved tissue preparation methods for single-nucleus and spatial genomics^16–19,24^; (3) deep donor characterization with all analytical methods applied to the same donors; (4) sufficient sampling to analyze the full diversity of cell types; (5) mapping profiled cells to the highly granular and curated BICCN cell type reference; and (6) validating cellular phenotypes across cortical areas, orthogonal modalities and independent datasets.

The current study focused on the middle temporal gyrus (MTG), an area involved in language and semantic memory processing^25^ and higher-order visual processing^26^. Many studies, from the histopathology of Braak and Braak^4^ to longitudinal studies of Tau positron emission tomography imaging^27,28^, demonstrate that MTG is a transition zone between aging-related or preclinical AD-related medial temporal lobe pTau and more advanced stages of AD, where neocortical pTau extends across the brain and is strongly correlated with dementia^4,29–31^. By combining temporal modeling of disease severity based on quantitative neuropathology with single-nucleus genomics and spatial analyses, this approach provides a comprehensive understanding of the specific, highly granular cell types affected over the course of disease, where those affected cells are located in tissue microarchitecture and when they are affected as disease progresses. This strategy to integrate data across studies to a common reference is highly extensible and provides a unifying framework for the AD community. Study data are freely available at the SEA-AD’s website (https://portal.brain-map.org/explore/seattle-alzheimers-disease).

## Results

### Profiling AD progression across pathological stages

**Fig. 1:**
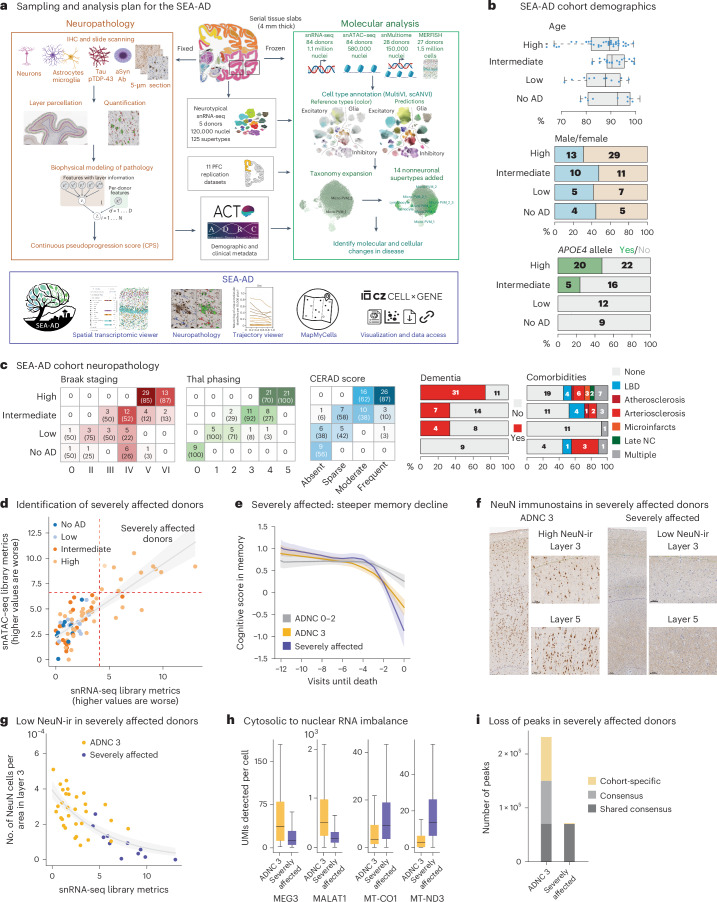
SEA-AD study of the MTG and cohort description. **a**, Schematic detailing the experimental design for applying quantitative neuropathology, snRNA-seq, snATAC–seq, snMultiome and MERFISH to the MTG of SEA-AD donors. **b**, SEA-AD cohort demographics, depicting age at death, biological sex and *APOE4* allele, stratified according to ADNC score. Age at death is represented by box-and-whisker plots with the box representing the interquartile range (IQR) and the whiskers representing 1.5 times the IQR. The solid line indicates the median. **c**, SEA-AD cohort composition stratified according to ADNC versus Braak stage Thal phase (left), and CERAD score as heatmaps, with dementia or comorbidities as bar plots. The number of donors in each box and the fraction are shown in parentheses. **d**, First PC for snRNA-seq versus snATAC–seq quality control metrics for each library color-coded according to ADNC category. The dashed red lines indicate the point where values are above 1.5 times the IQR. The gray line represents the linear regression (Pearson *R* = 0.80) **e**, The center lines represent the mean of the locally estimated scatter plot smoothing (LOESS) regression on longitudinal cognitive scores in the memory domain across ADNC 0–2 donors in gray, ADNC 3 donors in gold and ADNC 3 severely affected donors in purple. Uncertainty represents the s.e. from 1,000 LOESS fits with 80% of the data randomly selected in each iteration. **f**, Exemplar low-power and high-power micrographs showing the entire cortical column and cortical layers 3 and 5 from an ADNC3 donor (left) and a severely affected donor lacking NeuN-ir (right). Immunostaining was performed in the entire SEA-AD cohort (*n* = 84). **g**, Scatter plot showing the number of NeuN immunoreactive cells per area in cortical layer 3 versus the PC for snRNA-seq in **d**. Severely affected donors (purple) localize at the end of this trajectory. Gray, logistic regression; error bars, s.d. **h**, Box-and-whisker plots showing the number of unique molecular identifiers (UMIs) detected per cell for MEG3 and MALAT1, MT-CO1 and MT-ND3, ADNC high donors or severely affected donors. Outliers are not shown. *n* = 543,252 represents the total number of cells across selected donors. **i**, Bar plot showing the number of chromatin accessible regions in 11 randomly selected ADNC high donors or severely affected donors. ‘Shared consensus’ are regions shared across both groups; ‘consensus’ denotes regions shared across members of each group; and ‘cohort-specific’ depicts peaks unique to some members of each cohort. The cohort demographics can be found in Supplementary Table 1. **f**, Scale bar, 100 μm. Schematics in **a** created using BioRender.com.

**Extended Data Fig. 1:**
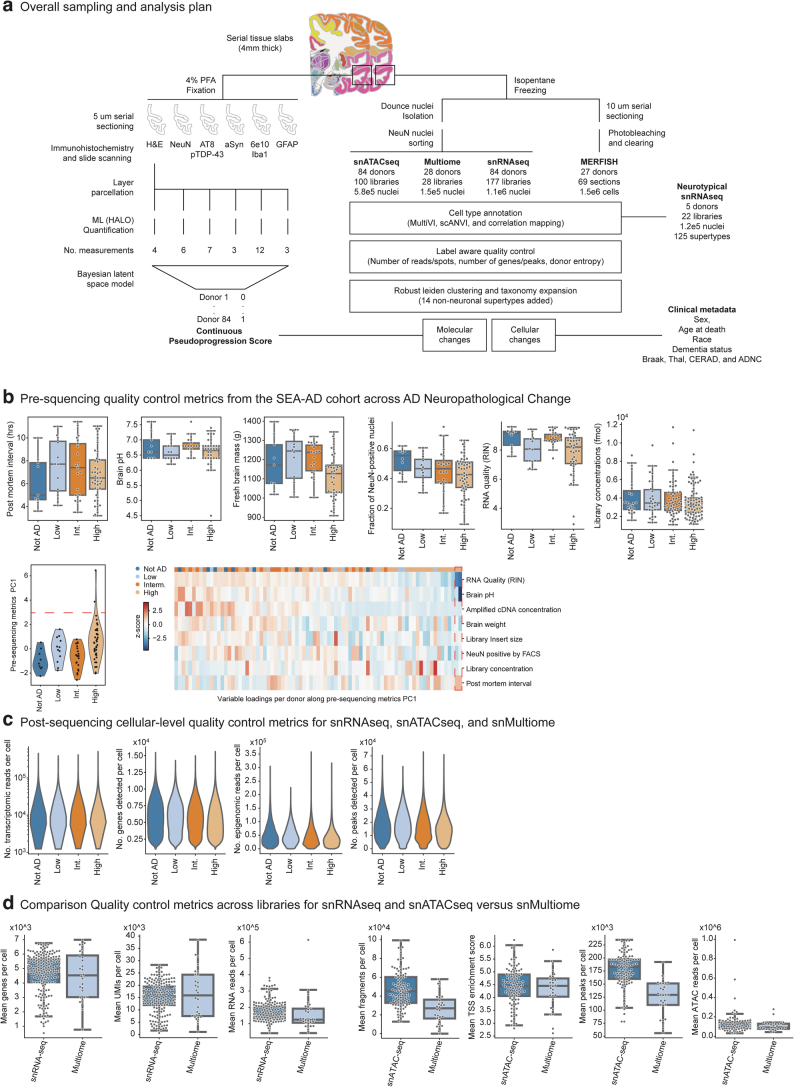
SEA-AD Brain Cell Atlas study design. **a**) Schematic detailing experimental design for applying quantitative neuropathology, single nucleus RNA sequencing (snRNAseq), single nucleus ATAC sequencing (snATAC-seq), single nucleus Multiome (Multiome), and multiplexed error robust fluorescence in situ hybridization (MERFISH) to middle temporal gyrus (MTG) of SEA-AD donors as well as the analysis plan for construction of a pseudo-progression score from quantitative neuropathology, integration across -omics data modalities, common cell type mapping to the BRAIN initiative reference, and use of demographic and clinical metadata to identify cellular and molecular changes in AD. **b**) Top, boxplots showing pre-sequencing quality control metrics for donor tissue (for example PMI, RIN, brain pH and mass) and single nucleus preparations (for example fraction of NeuN positive nuclei and library concentration) organized by AD Neuropathological Change (ADNC). Bottom, A donor by metric matrix was constructed for the values indicated, using a simple average for variables that had multiple values per donor (for example multiple sequencing library concentrations). Principle component analysis (PCA) was then run on the matrix. Bottom and left, Violin plot showing the eigenvalues for each donor along the first principal component organized by ADNC. Bottom and right, heatmap showing z-scores of the pre-sequencing quality control metrics (rows) in each donor (columns). Donors and metrics are ordered based on the first principal component eigenvalues and eigenvectors. Red dashed box, two outlier values along first principal component for two donors that were driven by low RIN and brain pH. N represents the total number of donors in SEA-AD, N = 84. **c**) Violin plots showing cellular-level post-sequencing quality control metrics for single nucleus transcriptomics, chromatin accessibility and multiome data organized by ADNC. Significant p-values: NeuN Fraction Not AD versus High=0.05. **d**) Violin plots comparing library-level post-sequencing quality control metrics of snRNA-seq to snMultiome (left) and snATAC-seq to snMultiome (right). N represents the total number of libraries profiled with snRNA-seq and Multiome, N = 205. Cohort demographic can be found in Supplementary Table 1.

To construct an integrated multimodal cellular atlas of AD and comorbid related disorders (AD/AD and related dementias) we generated (1) quantitative neuropathological measurements, (2) single-nucleus RNA sequencing (snRNA-seq), single-nucleus assay for transposase-accessible chromatin with sequencing (snATAC–seq) and single-nucleus multiome (snMultiome), and (3) cellularly resolved spatial transcriptomics (multiplexed error-robust fluorescence in situ hybridization (MERFISH)) in the MTG from a cohort of 84 aged donors spanning the spectrum of AD pathology (Fig. 1a and Extended Data Fig. 1a). We collectively profiled 3.4 million high-quality nuclei across all modalities, mapping each to one of 139 molecular cell types from an expanded BRAIN Initiative MTG cellular taxonomy^18^ that included disease-associated states. A continuous pseudoprogression score (CPS) was constructed from quantitative neuropathology, which ordered donors along a neuropathological continuum, and increased discovery power to identify molecular and cellular changes. To validate and replicate these results, we generated a similar 1.2-million nuclei snRNA-seq dataset from Brodmann area 9 (A9) in the same 84 donors, mapping to a matched BRAIN Initiative A9 taxonomy. To replicate findings, we uniformly reprocessed ten publicly available datasets that applied snRNA-seq to 4.3 million high-quality nuclei from the prefrontal cortex (PFC), which includes the A9 of 707 additional donors also spanning the spectrum of AD pathology. These multimodal datasets, tools to explore them and tools to map new datasets to this new cellular taxonomy are all available at SEA-AD.

The SEA-AD cohort was derived from longitudinally characterized research brain donors from the community-based Adult Changes in Thought (ACT) study and the University of Washington (UW) Alzheimer’s Disease Research Center (ADRC)^32–35^. Brains were collected using highly optimized brain preparation methods (mean postmortem interval = 7.0 h; Extended Data Fig. 1b) that enable exceptionally high-quality snRNA-seq, snATAC–seq and MERFISH profiling^17–19,23,36,37^. Donors were included if death occurred within the specific time of data collection (except for specific exclusion criteria noted in the Methods) (Supplementary Table 1). SEA-AD includes donors across the range of AD neuropathological change (ADNC) (nine, no AD; 12 low; 21 intermediate; 42 high ADNC) who were all aged (minimum age at death = 65, mean = 88; Fig. 1b, top).

Female donors outnumbered male donors (51 females, 33 males), particularly in those with high ADNC (29 females, 13 males), which is consistent with the known prevalence of AD in females^38^ (Fig. 1b, middle). Donors with an *APOE4* allele included nearly half (20 of 42) of high ADNC cases, a quarter (five of 21) of intermediate cases and no low ADNC or no AD cases (Fig. 1b, bottom). Braak stage (tangles), Thal phase (plaques) and Consortium to Establish a Registry for Alzheimer’s Disease (CERAD) (neuritic plaques) increased as expected with ADNC (Fig. 1c, left). Nearly three-quarters (31 of 42) of high ADNC cases had dementia before death, versus a third in intermediate (seven of 21) and low (four of 12) ADNC cases, and none in no AD cases (Fig. 1c, middle). Donors with any level of Lewy body dementia (LBD), vascular pathology or limbic-predominant age-related TDP-43 encephalopathy (LATE) were included because these conditions are common comorbidities in AD^39,40^. Roughly half (42 of 84) had one or more severe copathologies (Fig. 1c, right).

**Extended Data Fig. 2:**
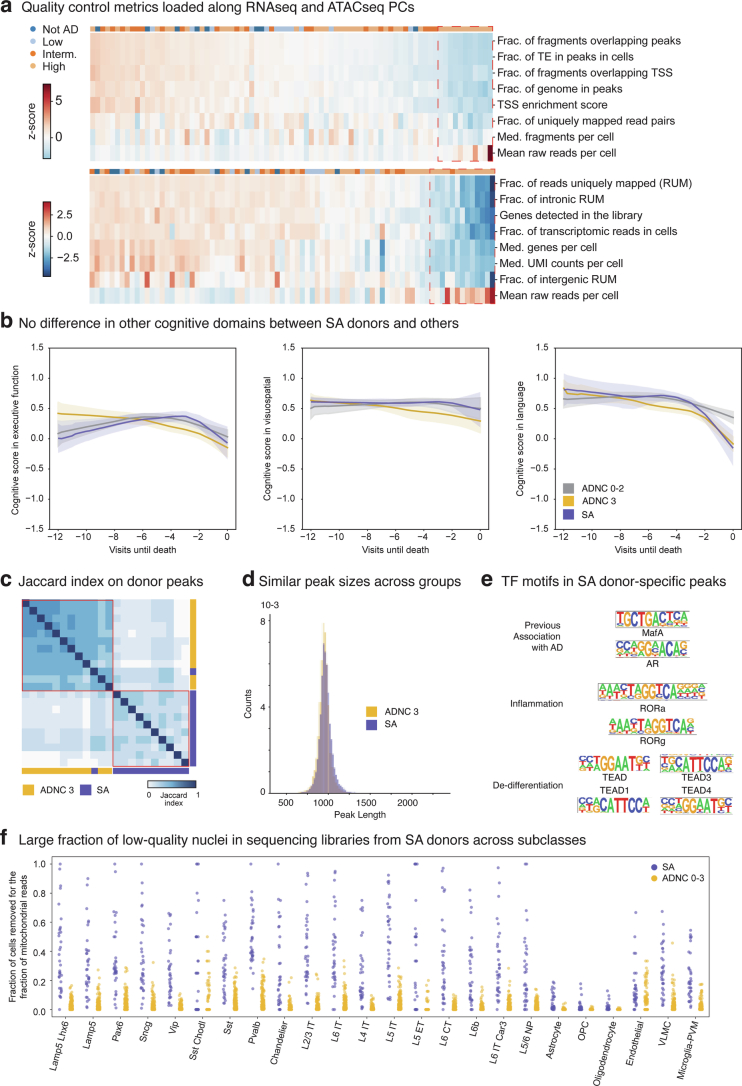
Altered multimodal metrics within severely affected donors. **a**) Donor by metric matrices were constructed for the library-level post-sequencing quality control values indicated, using a simple average when multiple libraries were sequenced per donor. Principle component analysis (PCA) was then run on each matrix. Heatmaps showing z-scores of snATAC-seq (top) and snRNA-seq (bottom) metrics (rows) in each donor (columns). Donors and metrics are ordered based on the first principle component eigenvalues and eigenvectors. Red dashed boxes, donors with outlier eigenvalues along each PC. **b**) LOESS regression on longitudinal cognitive scores in the executive, visuospatial, and language domain across ADNC 0-2 (Not AD to Intermediate) in grey, ADNC 3 donors that were not severely affected in gold, and ADNC 3 donors that were in purple. Center lines are the mean from LOESS fits; uncertainty represents the standard error from 1000 LOESS fits with 80% of the data randomly selected in each iteration. Significant p-values for cognitive decline in language: SA donors versus ADNC 0-2 = 0.009, Other ADNC 3 versus ADNC 0-2 = 0.021. Statistical test is a multinomial logistic regression as described in methods’ section Testing for differential cognitive slopes. **c**) Heatmap showing the pairwise jaccard distances based on the peak universes from 11 randomly selected ADNC 3 donors (yellow) and all 11 severely affected donors (purple) hierarchically ordered. Red boxes, two clusters within the hierarchy that largely correspond to the separation between ADNC3 and SA donors. **d**) Histogram showing the distribution of peak lengths of accessible regions in ADNC 3 (yellow) and severely affected donors (purple). **e**) Transcription factors binding sites enriched in chromatin accessible regions uniquely found in severely affected donors organized by their gene ontology category. Transcription factors that bind to them are indicated. **f**) Stripplot showing the fraction of cells removed from each library for having too many mitochondrial reads during quality control organized by subclass and by severely affected donors (purple) and ADNC 0–3 donors (yellow). Cohort demographic can be found in Supplementary Table 1.

Nearly all (82 of 84) had high presequencing quality control metrics (for example, brain pH, RNA integrity number (RIN) scores and sequencing library yield) across the whole range of disease severity (Extended Data Fig. 1b), with two outlier samples excluded because of low RIN and brain pH. Post-sequencing metrics were also uniformly high across disease severity (Extended Data Fig. 1c), suggesting no inherent tissue quality degradation related to advanced age and neuropathology in most donors. However, principal component analysis on snRNA-seq and snATAC–seq library-level metrics identified a subset of high pathology donors (11 of 42, 26.2%) with slightly lower-quality data in both modalities (Fig. 1d and Extended Data Fig. 2a). Longitudinal cognitive testing in our cohort spanned four cognitive domains (memory, executive, language and visuospatial function^41^). These donors had steeper memory decline compared to other high pathology donors (slopes in memory decline = −0.15 in severely affected donors versus −0.11 in all other high ADNC donors; *P* value with no AD and low ADNC donors as base outcome = 0.01 versus 0.15; Fig. 1e); other cognitive domains showed a similar trajectory across groups (Extended Data Fig. 2b). Immunoreactivity to neuronal nuclear protein (NeuN-ir) was previously shown to anticorrelate with pTau pathology^42^. These 11 donors had a pronounced reduction in NeuN-ir that was not due entirely to cell loss (Fig. 1f,g). Given the steeper cognitive decline and effects on multiple data modalities, we named these donors severely affected.

Despite having more reads per nucleus, snRNA-seq libraries from severely affected donors had fewer UMIs, genes detected, uniquely mapped reads (mostly reflecting increased ribosomal RNA) and reads with introns (reflecting mRNA versus pre-mRNA) (Extended Data Fig. 2a). Nuclei from severely affected donors had lower nuclear-localized RNA^43^ (for example, *MALAT1* and *MEG3*) and higher cytosolic localized RNA (for example, RNA from mitochondrially encoded genes) compared to other high ADNC donors (Fig. 1h). To disentangle whether reduced nuclear representation was due to global transcriptional shutdown or degradation, we computed open chromatin peaks from high pathology donors and assessed their similarity according to Jaccard distance. The chromatin landscape segregated the 11 severely affected donors from matching high ADNC donors (Extended Data Fig. 2c). We saw no difference in consensus peak length distributions between groups (Methods and Extended Data Fig. 2d). However, severely affected donors showed many fewer peaks (Fig. 1i), which were almost entirely a subset of peaks seen in other high pathology donors. Notably, there was a small number of peaks (*n* = 1,574) unique to severely affected donors that were enriched for binding motifs for transcription factors associated with inflammation, dedifferentiation and AD pathology (Extended Data Fig. 2e). Taken together, these results suggest that severely affected donors underwent global chromatin repression leading to transcriptional shutdown. As severely affected donors showed systematically lower data quality (Extended Data Fig. 2f), we excluded them from the analyses on gene expression changes.

### Quantifying the progression of AD severity

**Fig. 2:**
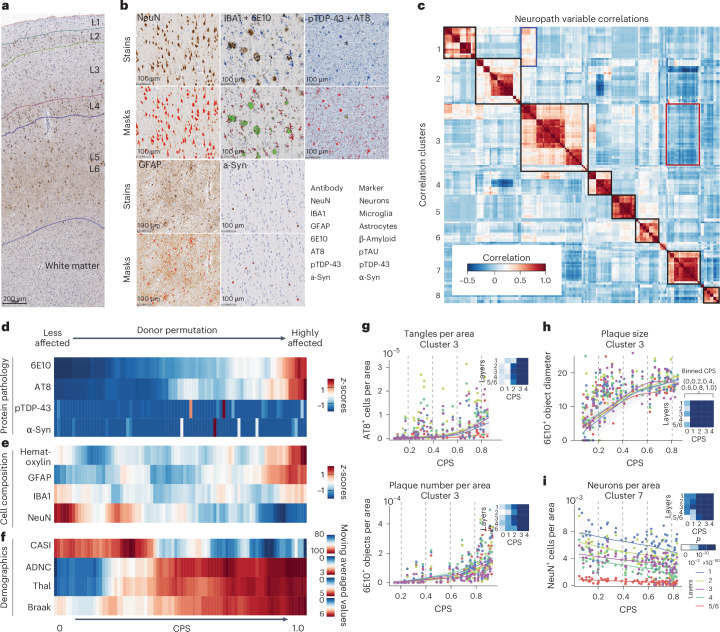
MTG quantitative neuropathology orders donors according to pseudoprogression of disease. **a**, Representative cortical column visualized with immunohistochemistry (IHC). Cortical layers (L1–L6) and white matter are indicated. Immunostaining was performed in the entire SEA-AD cohort (*n* = 84). **b**, Higher-powered micrographs showing IHC staining for protein aggregates and cellular populations. Bottom, masks showing positive voxels generated by HALO in red for single staining and both red and green for duplex staining. Immunostaining was performed in the entire SEA-AD cohort (*n* = 84). **c**, Heatmap showing a hierarchically organized co-correlation matrix of quantitative neuropathology variables. The black boxes on the diagonal indicate eight correlated clusters. The red box indicates the anti-correlation representing AD protein pathologies and NeuN immunoreactivity (NeuN-ir), respectively. The blue box indicates the correlation between variables related to NFTs and pTDP-43 variables. **d**, Heatmap showing the number of pathological protein objects detected per unit area across all cortical layers in each donor, ordered along a CPS. All values were converted to *z*-scores and adjusted according to a moving average. **e**, Heatmap showing the number of cellular objects detected per unit area across all cortical layers, ordered along the CPS. Hem, hematoxylin^+^ nuclei; GFAP, IBA1 and NeuN indicates the number of positive cells. All values were converted to *z*-scores and adjusted according to a moving average. **f**, Heatmap showing the cognitive scores at the last visit (CASI) and AD pathology stage (ADNC, Thal, Braak), ordered along the CPS. All values were adjusted according to a moving average. **g**–**i**, Scatter plots showing how specific quantitative neuropathological variables relate to CPS. The dots represent donor values in the cortical layer; the lines are LOESS regressions within each layer. **g**,**h**, Cluster 3 consists of variables increasing along pseudoprogression, such as the number of AT8^+^ cells per unit area, 6E10^+^ objects per unit area (**g**) or the average 6E10^+^ object diameter of the 6E10-ir Aβ plaques (**h**). **i**, Cluster 7 included variables decreasing their value along CPS, such as the number of NeuN^+^ cells or percentage NeuN-ir cell area. The heatmap on each quantifiable neuropathological variable across layers represents the *P* value from a general additive model. *P* values are the two-tailed *P* values for the *t*-statistics of the parameters as described in the Python package statsmodels. The cohort demographics can be found in Supplementary Table 1. **a**, Scale bar, 200 μm.

**Extended Data Fig. 3:**
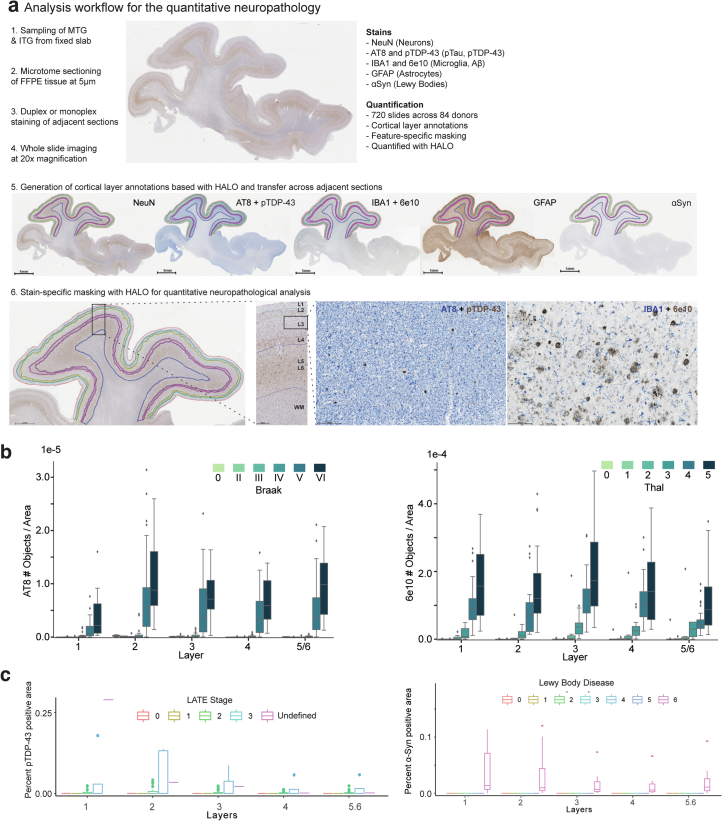
Human MTG neuropathological stains track brain-wide pathological states. **a**) Schematic depicting neuropathological data acquisition pipeline (ordered 1 to 6). **b**) Boxplots showing the number of pTau-bearing cells per unit area organized by Braak stage (left) and number of Aβ plaques per unit area organized by Thal phase (right) across donors. Note, in later stages there is considerable variability in plaque and tangle number, underscoring limitations in classical staging. **c**) Boxplots showing the percent of pTDP-43-positive voxels (left) and percent of α-Syn-positive (α-Synuclein) voxels across donors organized by to LATE-NC stage (left) and Lewy Body Disease stage (right). Lewy Body Disease is coded numerically (0=Not or Incompletely Assessed, 1=Not Identified, 2=Amygdala-predominant, 3=Brainstem-predominant, 4=Limbic (Transitional), 5=Olfactory bulb only, 6=Neocortical). Note, only donors in later stages have large accumulation of co-pathology. Cohort demographic can be found in Supplementary Table 1.

To create a quantitative aggregate metric of the local burden of pathology that accompanies AD progression, we first used machine learning approaches to quantify neuropathological variables (Extended Data Fig. 3a). This included markers for conventional AD neuropathological staging, including pTau (AT8) for NFTs and Aβ (6E10) for amyloid plaques, and additional markers for associated comorbid pathologies (pTDP-43, alpha synuclein (α-Syn)) and cellular changes (ionized calcium-binding adapter molecule 1 (IBA1) for microglia, glial fibrillary acidic protein (GFAP) for astrocytes, NeuN for neurons and hematoxylin and eosin to assess cytopathology and white matter integrity; Fig. 2a,b and Supplementary Table 2).

The number of Aβ plaques and pTau^+^ neurofibrillary-tangle-bearing neurons in each donor were consistent with traditional staging thresholds for Braak stage and Thal phase, respectively (Extended Data Fig. 3b). However, at higher Braak stages and Thal phases, we observed high variability in pathological burden that underscored the limitation of classical staging (Extended Data Fig. 3b). pTDP-43 and α-Syn pathologies were detected in the relatively small number of donors with high-stage LATE-NC^44^ and neocortical LBD, respectively (Extended Data Fig. 3c). Cross-correlation of the quantifiable neuropathological variables followed by hierarchical clustering revealed eight biologically coherent clusters (Fig. 2c), with two anticorrelated clusters: cluster 3, which contained measurements of AD-related pathological proteins (that is, diameter of Aβ plaques, number of Aβ plaques or pTau-bearing cells); and cluster 7, which contained NeuN-ir in neuron-related variables (that is, the number of NeuN-ir nuclei per area).

**Extended Data Fig. 4:**
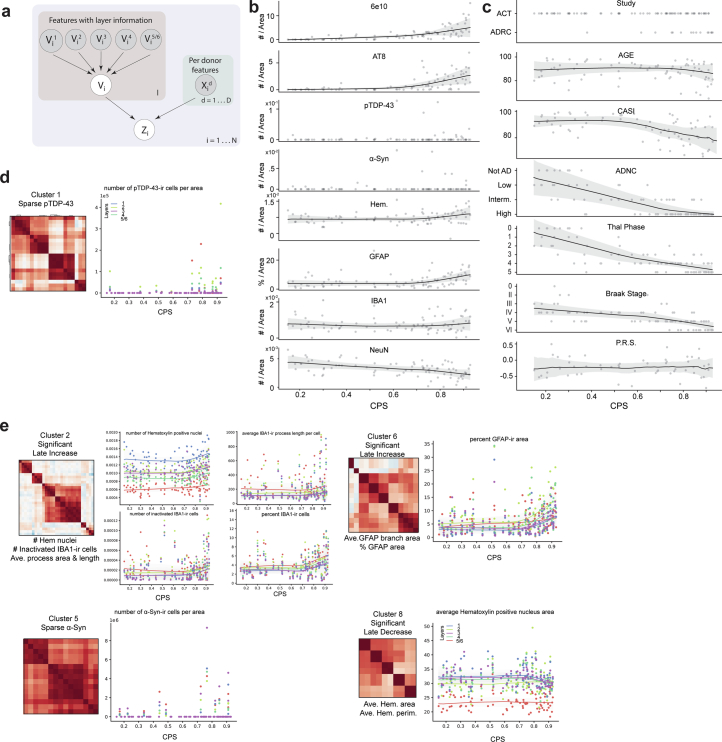
MTG pseudo-progression scores orders quantitative neuropathological variables following increasing disease severity. **a**) Graphical model used to infer the continuous pseudo-progression score (CPS). **b, c**) LOESS regression plots relating mean quantitative neuropathological (QNP) variables across layers (B) and demographic/clinical metadata (C) indicated to CPS. Dots represent individual donor values. Uncertainty in each line represents the standard error from 1000 LOESS fits with 80% of the data randomly selected in each iteration. Note, variables from (C) were not used to construct the model. CASI, Cognitive Abilities Screening Instrument; ADNC, AD Neuropathological Change; PRS, Polygenic Risk Score. **d**) Left, Subset of heatmap from Fig. 2c showing co-correlation of QNP variables in cluster 1. Right, Scatterplot showing how the QNP variable number of pTDP-43 positive cells per unit area, which is within correlation cluster 1, relates to CPS. Dots represent values from each donor in the cortical layer indicated, lines are LOESS regressions for measurements across donors within each layer. **e**) Same plots as in (D) but for clusters 2, 6, 5, and 8. Cohort demographic can be found in Supplementary Table 1.

Inspired by biophysical studies^45^, which suggest that pathology aggregates exponentially in AD, we constructed a Bayesian model to infer AD pathological burden from the trajectory of each quantifiable neuropathological variable. The models assigned a continuous pseudoprogression score (CPS) from 0 to 1 to each donor (Extended Data Fig. 4a). Along the CPS, the number of pathological pTau-bearing neurons and Aβ plaques increased exponentially across donors (Fig. 2d and Extended Data Fig. 4b). There was no clear relationship to pTDP-43 and α-Syn levels. The number of NeuN-ir nuclei decreased along the CPS but had linear dynamics. Later in the CPS, in donors with the highest pathological burden, we observed an increased number of nuclei detected per area of GFAP^+^ nuclei (Fig. 2e and Extended Data Fig. 4b), which is consistent with later-stage astrogliosis. Importantly, CPS correlated with independent clinical data not included in the model, including Braak stage, Thal phase, ADNC score and cognitive scores (Cognitive Abilities Screening Instrument (CASI)), but not other covariates such as age (Fig. 2f and Extended Data Fig. 4c).

To understand quantifiable neuropathological dynamics, we divided CPS into five equal bins and determined whether significant changes occurred in each with a generalized additive model. Cluster 3 included several variables related to plaque and tangle pathology that mostly had their first significant increases later in the CPS (Fig. 2g). Specifically, a CPS of 0.4–0.6 (bins 2 and 3) was a critical point when pTau-bearing cells and Aβ plaques started accumulating more substantially and cognitive deficits increased. Within cluster 3, Aβ plaque diameter increased early (Fig. 2h), with significant change starting at a CPS of 0.2 (bin 1), suggesting that other Aβ species such as peptides and oligomers may be present. NeuN immunoreactivity decreased significantly along the CPS (Fig. 2i). Furthermore, we observed an interaction between clusters 1 and 3 (Fig. 2c, blue box and Extended Data Fig. 4d) that captures the accumulation and colocalization of pTDP-43 inclusions in pTau-bearing cells, as described previously^46^. Most of the remaining variables displayed significant increases after CPS bin 3 (Extended Data Fig. 4e). Taken together, CPS captures AD severity in a continuous quantitative metric and defines two epochs: (1) an early epoch where donors have low levels of pathology and are cognitively unaffected but exhibit neuronal loss and evidence of early amyloid pathology; and (2) a late epoch where donors have markedly increased levels of AD pathology, neuronal loss and cognitive impairment.

### Constructing an integrated, multimodal AD atlas in the MTG

**Fig. 3:**
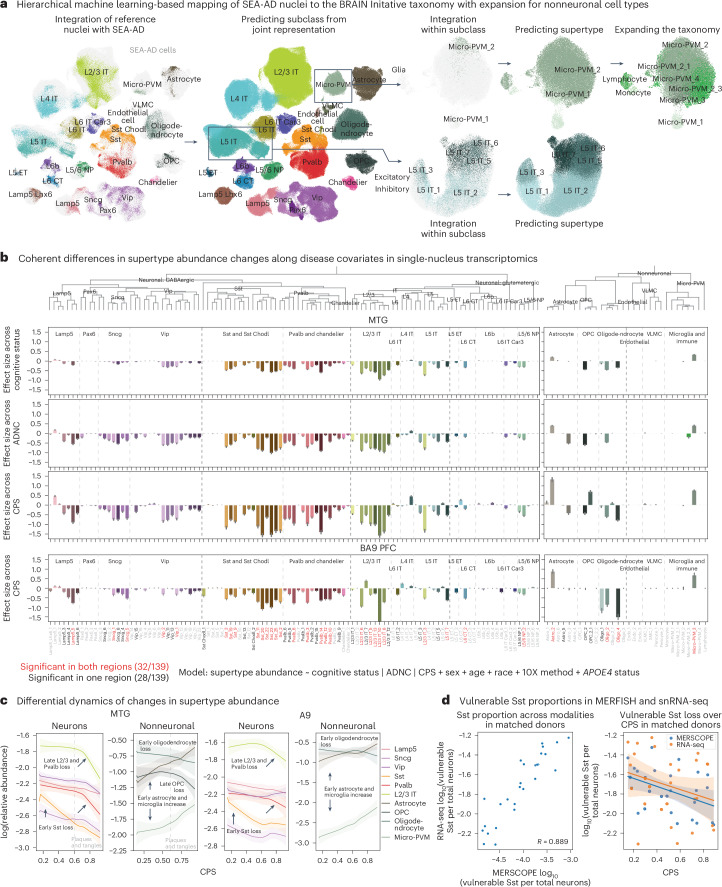
Vulnerable populations in the MTG concentrate around superficial supragranular layers. **a**, Schematic showing the hierarchical mapping procedure used to create the SEA-AD taxonomy and annotate all SEA-AD cells. Reference MTG cells were used to define neuronal supertypes (Methods). SEA-AD nuclei are colored light gray. Cell subclasses and supertypes are indicated. **b**, Bar plots showing the effect sizes for relative abundance changes in MTG associated with cognitive status (top), ADNC (middle) or CPS (bottom), controlling for sex, age, single-cell technology and *APOE4* status. Below, effect sizes for A9 across CPS, controlling for sex, age at death and race. Red, significantly changed in both cortical regions; dark gray, significantly changed in one cortical region; light gray, not significantly changed. The light gray lines separate subclasses in the same cellular neighborhood; darker gray lines separate cellular neighborhoods. The bar plots and lines represent the average and s.e.m. over 139 compositional tests in which we rotated the reference population. In each test, *n* = 82 donors were used to fit the model. **c**, Center lines are the mean of the LOESS regressions relating the log-normalized relative abundance (within all neuronal or all nonneuronal nuclei) of supertypes that were significantly changed in the MTG (two plots on the left) or A9 (two plots on the right) to the CPS. Supertypes were grouped according to their subclasses to facilitate visualization of how each set of supertypes changed. Sst supertypes decreased in their relative abundance early in CPS, before an exponential increase in the number of plaques and tangles present (indicated on each plot with a dashed light gray line). In contrast, L2/3 IT and Pvalb supertypes decrease as AD pathology increases. Uncertainty in each line represents the s.e. from 1,000 LOESS fits with 80% of the data randomly selected in each iteration. **d**, Left, scatter plot showing the correlation of vulnerable Sst supertype relative abundance in snRNA-seq and MERFISH data from matched donors (*R* = 0.84). Right, scatter plot relating the relative abundance of vulnerable Sst supertypes to CPS in the snRNA-seq (orange) and MERFISH (blue) datasets from the same donors. The lines represent the linear regression fits; the error bars are the s.e. from 1,000 bootstraps using 80% of the data in each. The cohort demographics can be found in Supplementary Table 1.

**Extended Data Fig. 5:**
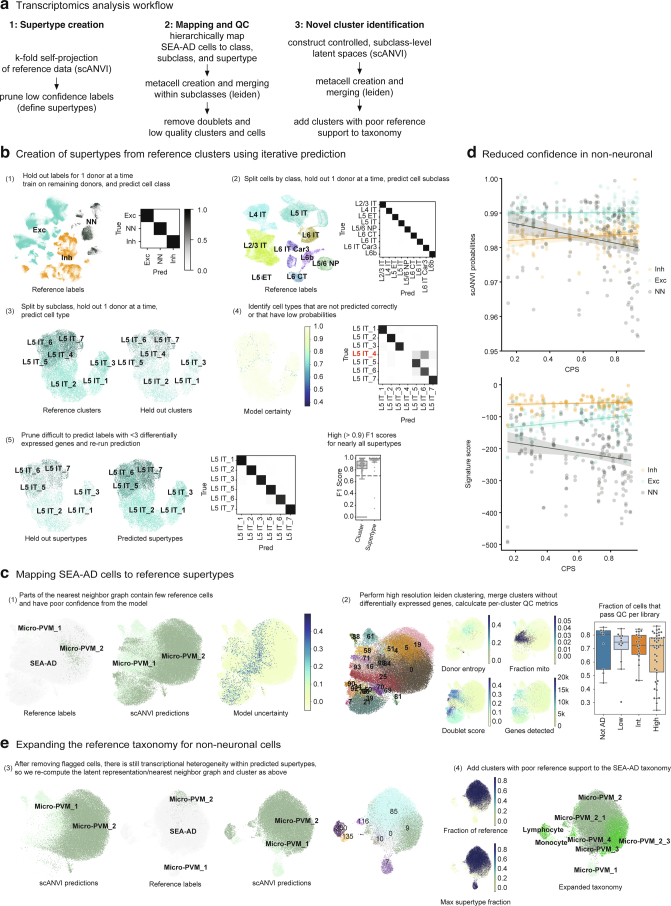
Pipeline for the creation of the SEA-AD MTG taxonomy. **a**) Schematic showing steps involved in supertype creation from snRNA-seq data in neurotypical reference donors. **b**) Hierarchical procedure for the creation of robustly mappable cell types, termed supertypes. Labels from 1 of 5 reference donors was systematically held out and predicted using a deep generative model (DGM) trained on the remaining 4 donors. Steps 1 to 3 represent mapping cells to one of three classes, splitting each class and mapping to one of 24 subclasses, splitting each subclass and mapping to one of 151 clusters from the original BRAIN Initiative taxonomy. 26 of 151 clusters were pruned, mostly representing cell types that were intermediates of others. Finally, in step 5 we repeat mapping with the 125 highly mappable supertypes and show consistently high F1 scores across them (box and whisker plot). **c**) After hierarchically mapping SEA-AD nuclei to supertypes using the same approach as above, we filtered low quality nuclei within subclasses (The microglia subclass is shown as an example). Left, scatterplots showing the UMAP coordinates of all SEA-AD and reference nuclei within the microglia subclass. In the first plot, reference nuclei are labeled and colored and SEA-AD nuclei are in light grey. In the second and third plots, we show the supertype predictions for each nucleus from the DGM as well as the uncertainty in the prediction (darker nuclei are more uncertain). In the fourth plot we show robust, high resolution Leiden clusters and color them by their quality control metrics (that is donor entropy, mean fraction of mitochondrial reads, mean doublet score, and mean number of genes detected). **d**) Scatterplots showing scANVI probabilities (top) and supertype signature scores (bottom) organized by cell classes. Lines represent linear regressions. Note, decreasing probabilities and signature scores for non-neuronal supertypes, but not others. **e**) After removing low quality nuclei new latent representations were learned with DGMs, which were then underwent robust Leiden clustering. Clusters with low fractions of nuclei from neurotypical reference donors ( < 10%) were added to the taxonomy. Cohort demographic can be found in Supplementary Table 1.

**Extended Data Fig. 6:**
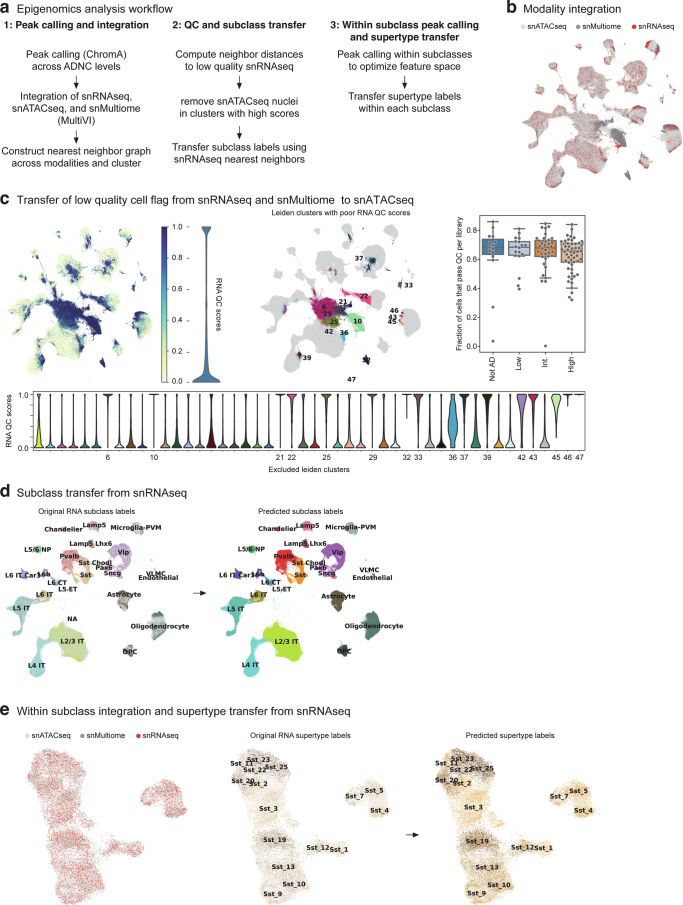
Pipeline for the annotation of chromatin accessibility data sets. **a**) Schematic showing steps involved in processing the SEA-AD snATAC-seq data, which include global peak calling and modality integration, quality control filtering and subclass mapping, and within subclass peak calling and supertype mapping. **b**) Scatterplot showing the UMAP coordinates of all nuclei profiled in the middle temporal gyrus (MTG) color coded by indicated data modalities. **c**) Top and left, Same scatterplot as in (B) but color coded by low quality cell score (left) and (right) by Leiden clusters with mean low quality cell scores greater than 0.5. Violin plot to the right of the first plot shows the binary distribution of the low quality cell scores (RNA QC score). Bottom, violin plots showing the distribution of the low quality cell score per Leiden cluster, with the number of those that were flagged indicated. Top and right, box and whisker plot showing the fraction of cells in each snATAC-seq library that were filtered during quality control. **d**) Scatterplots showing the UMAP coordinates from (B) of only the high quality nuclei colored by neurotypical reference subclasses versus SEA-AD in light grey (left) and by predicted subclass (right). **e**) Scatterplots showing UMAP coordinates of nuclei from 1 example subclass (Sst) based on integrated space constructed with subclass-specific peaks. Plots are color coded by modality (left), by reference supertypes versus SEA-AD in light grey (middle) and by predicted supertype (right). Cohort demographic can be found in Supplementary Table 1.

Previous BICCN efforts identified 151 transcriptionally distinct cell types and states in the MTG from neurotypical adult reference donors^17^, hierarchically organized into 24 subclasses (for example, L2/3 intratelencephalic-projecting excitatory neurons or L2/3 IT) within three main classes (excitatory neurons, inhibitory neurons and nonneuronal cells). We used this BICCN reference as a base to construct a cellular taxonomy for SEA-AD. To map SEA-AD data to cell types consistently across all 84 donors, we first defined robust transcriptional types, named supertypes, in the BICCN reference; 125 supertypes represented cell types that could be reliably reidentified in reference datasets (mean F1 score = 0.91) using hierarchical probabilistic Bayesian mapping^47,48^(Extended Data Fig. 5a,b). We then mapped SEA-AD snRNA-seq and snMultiome nuclei to these supertypes using the same mapping method (Fig. 3a). After removing low-quality nuclei (Extended Data Fig. 5c), we noted some nonneuronal nuclei that had systematically lower mapping scores, which suggested SEA-AD-specific cell types or states (Extended Data Fig. 5d). We used a clustering-based approach to identify and add 14 nonneuronal cell types or states to the final SEA-AD taxonomy of 139 supertypes (Fig. 3a, Extended Data Fig. 5e and Methods). A9 snRNA-seq data from the same SEA-AD donors were mapped to a matched A9 BRAIN Initiative cellular taxonomy using identical methods. We then extended our transcriptionally defined supertypes across snRNA-seq, snATAC–seq and snMultiome datasets to construct a joint multiomic representation^49^ from both neurotypical reference donors and donors with the disease (Extended Data Fig. 6a–e).

**Extended Data Fig. 7:**
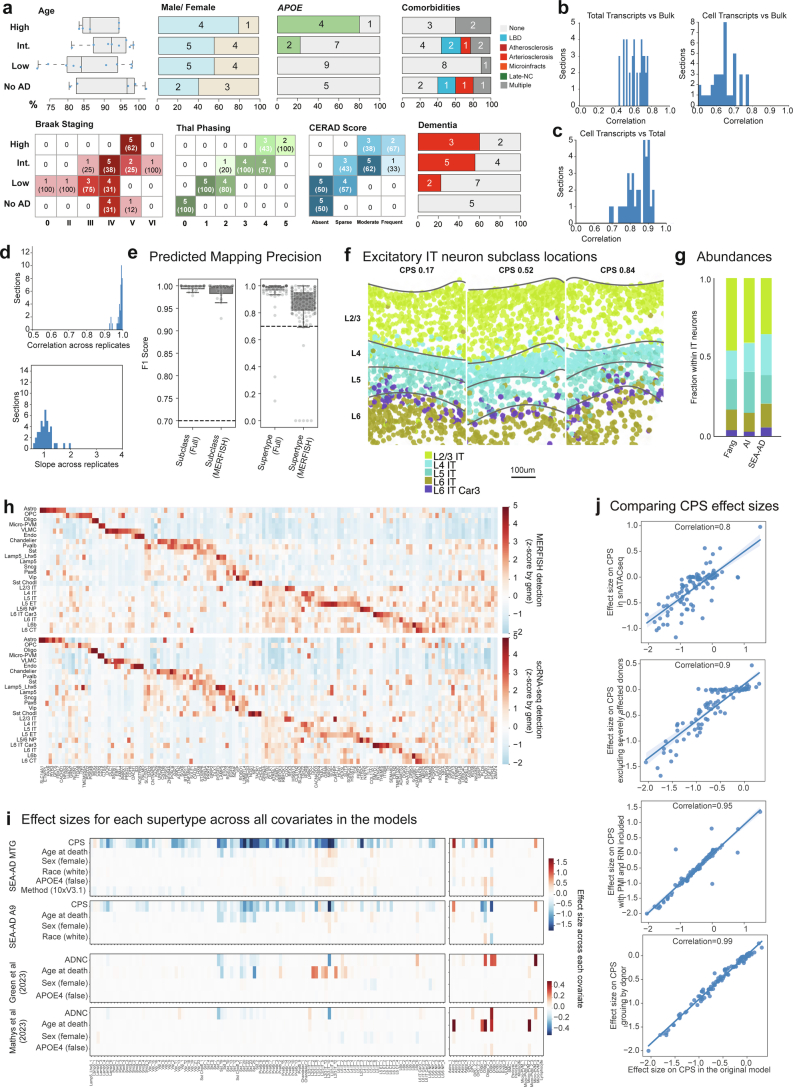
Pipeline for the acquisition of high quality spatial transcriptomic data in the human MTG. **a**) Top, SEA-AD MERFISH cohort demographics stratified by AD neuropathological change (ADNC) score. Numbers indicate the number of donors in each group. **b**) Histograms showing the correlation between total slide transcripts (left) or transcripts within cells (right) and bulk RNAseq across sections. **c**) Histogram showing the correlation between total slide transcripts and transcripts in cells. **d**) Left, histogram showing the correlation in total slide transcripts across sections from the same donor. Right, Histogram showing the slope from a linear regression comparing total slide transcripts across sections from the same donor. **e**) Box and whisker plot showing F1 scores for subclasses (left) and supertypes (right) from the procedure where 1 donor was systematically held out at a time in neurotypical reference snRNA-seq data where the model could use all genes (Full) or only the 140 genes in the MERFISH panel (MERFISH). **f**) Scatterplots showing the positions of excitatory IT neurons as dots from example sections from donors with an early (0.17), middle (0.52) and late (0.84) CPS color coded by their subclass. **g**) Barplot showing the relative abundance of excitatory IT neurons across data collection efforts in neurotypical specimens from previous studies compared to SEA-AD data. **h**) Heatmaps showing the average gene expression levels of genes included in the 140 gene MERFISH panel at the subclass level in snRNA-seq (top) and MERFISH (bottom) data from MTG. **i**) Heatmaps showing the effect sizes of relative abundance changes along each covariate from neuronal (left) and non-neuronal (right) scCODA models MTG dataset, the SEA-AD snRNA-seq A9 dataset, Green et al. (2023) snRNA-seq dataset, and Mathys et al. (2023) snRNA-seq dataset. **j**) Scatterplots relating the effect sizes of each supertype along CPS from scCODA model on SEA-AD MTG dataset to a similar model run on SEA-AD snATAC-seq MTG dataset, to a model run without the severely affected donors, then including post-mortem interval (PMI) and RIN as covariates (third) and grouping data by donor instead of by library. Cohort demographic can be found in Supplementary Table 1.

To define the spatial distribution of supertypes and to validate cellular changes, we generated a large-scale, cellularly resolved MERFISH dataset, using a 140-gene panel (Supplementary Table 3) and including 69 sections from a subset of SEA-AD donors (*n* = 27; Extended Data Fig. 7a). This dataset passed stringent quality control metrics; when compared against bulk RNA-seq from brain samples and correlated transcript counts across whole-tissue sections, it also exhibited high donor technical reproducibility and high supertype mapping accuracy (Extended Data Fig. 7b–e). After mapping each cell in the spatial transcriptomic dataset to subclasses and supertypes, we found concordance between expected and mapped spatial distributions; for example, excitatory intratelencephalic (IT) subclasses were restricted to expected cortical layers and matched proportions observed in previous studies of neurotypical MTG tissue^17,50^ (Extended Data Fig. 7f,g). There was also high qualitative correspondence in gene expression across subclasses between donor-matched snRNA-seq and MERFISH data (Extended Data Fig. 7h).

### Vulnerable and disease-associated supertypes

To identify vulnerable and disease-associated cell populations as a function of AD progression^9,51–55^, we analyzed changes in supertype abundance across cognitive status, ADNC and CPS in the MTG snRNA-seq, snATAC–seq and snMultiome datasets. We conducted tests for neuronal and nonneuronal cells separately as we sorted these populations separately (Methods). Multiple neuronal and nonneuronal supertypes decreased in relative abundance as a function of disease severity, while a few highly specific nonneuronal supertypes increased (Fig. 3b). A similar pattern of changes in supertype abundance was seen for all disease metrics, with 36 of 139 (26%) supertypes significantly affected (mean inclusion probability greater than 0.8) across each disease-related covariate. The number and effect sizes of the affected supertypes were significantly less in other covariates; we observed consistent results with and without the severely affected donors and in other single-nucleus data modalities (Extended Data Fig. 7i,j and Supplementary Table 4).

Only a subset of supertypes in most subclasses were affected, highlighting the importance of analysis at high cellular granularity. We refer to cell types that decrease in their relative abundance along the CPS as vulnerable, those that increase as associated, those that are unchanged as unaffected and those encompassing vulnerable and associated supertypes as affected. The extensive annotation of the BICCN reference enabled meaningful interpretation of affected cells. The vulnerable neuronal supertypes included a subset of excitatory IT neuron types largely in layer 2 or 3 (L2/3 IT), a subset of GABAergic interneuron types derived from the medial ganglionic eminence (MGE) (somatostatin inhibitory (Sst) and Pvalb) and caudal ganglionic eminence (CGE) (Vip, Lamp5 and Sncg) (Fig. 3b, left). Among nonneuronal populations that were affected, we observed increases in one microglial and one astrocytic supertype and decreases in one oligodendrocyte and one oligodendrocyte progenitor cell (OPC) supertype (Fig. 3b, right). Sst interneuron and oligodendrocyte supertypes decreased early and continuously with CPS, accompanied by increases in microglial and astrocyte supertypes (Fig. 3c, left). Notably, L2/3 IT neurons and Pvalb interneurons decreased sharply at high CPS. More than half (32 of 58) of supertypes affected in the MTG also changed in the same donors in A9, affected later in disease progression, including nearly all types (32 of 34) showing changes in A9 (Fig. 3b). The dynamics of supertype changes with CPS were also remarkably similar across regions (Fig. 3c, left). Spatial transcriptomics corroborated the vulnerability of specific Sst supertypes. The relative abundances of vulnerable Sst neurons were highly correlated (correlation = 0.84) between the snRNA-seq and MERFISH datasets (Fig. 3d, left) and there was a consistent decline in Sst supertypes across modalities (Fig. 3d, right).

**Extended Data Fig. 8:**
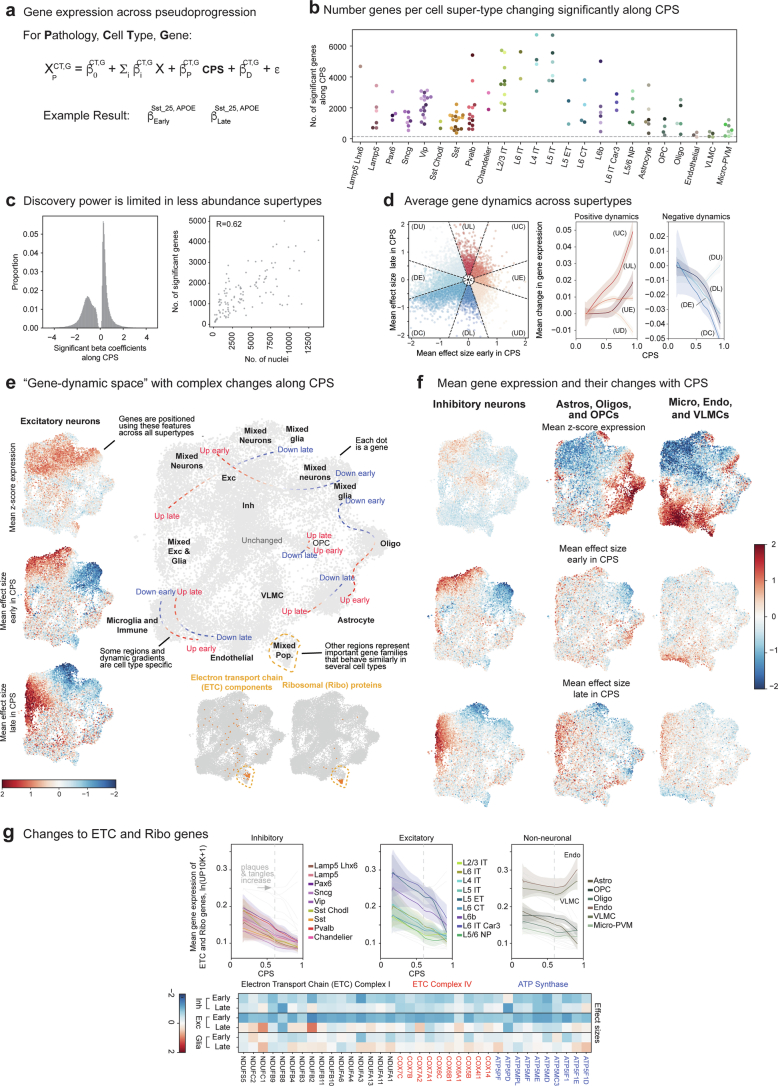
Construction of the gene-dynamic space. **a**) Schematic for identifying differentially expressed genes in each disease epoch along CPS using a generalized linear mixed model. **b**) Swarmplot showing the number of genes significantly changed with the continuous pseudo-progression score (CPS) in each supertype, organized by subclass. Grey dashed line, expected false discovery rate. **c**) Left, histogram showing the effect sizes across all supertypes of significantly changed along CPS. Note, many significant changes had relatively small effect sizes. Right, Scatterplot showing a weak (but present, R = 0.62) correlation between the number of nuclei and number of genes called as significantly changed along CPS. **d**) Scatterplot relating the mean effect size across supertypes of each gene estimated using donors from the early versus late disease epochs along CPS. Genes were categorized into 8 bins given their early and late effect sizes: DU, down up. DE, down early. DC, down consistently. DL, down late. UD, up down. UE, up early. UC, up consistently. UL, up late. Right, LOESS regression relating the mean expression of all genes in each category to CPS. **e**) Framework to unsupervised exploration of gene expression changes. Early and late effect sizes and z-scored mean gene expression values were collected across supertypes. Next, an unsupervised low-dimensional representation is built. Right, gene low dimensional representation qualitatively annotated to show areas of genes with cell type specific expression (black labels) and CPS gene expression dynamics (blue to red labels and dashed lines). **f**) Scatterplots of the gene-dynamic space colored by mean z-scored expression, early and late effect size across the supertypes in the cellular neighborhoods indicated. **g**) Top, LOESS regression relating the mean expression of electron transport chain (ETC) and ribosomal (Ribo) genes to CPS, color coded by inhibitory (left), excitatory (middle), and non-neuronal (right) subclasses. Dashed grey line, point in CPS when pathology is increasing (CPS = 0.6); error-bars are the standard error from 1000 bootstraps using 80% of the data in each. Bottom, heatmap displaying mean effect sizes across cell class for genes within the ATP synthase complex (blue) and complexes 1 (black) and 4 (red) from the electron transport chain. Cohort demographic can be found in Supplementary Table 1.

Finally, to understand molecular processes dysregulated by disease, we tested for expression changes along the CPS across each supertype (Extended Data Fig. 8a and Supplementary Table 5). The numbers of genes with significantly altered expression ranged from roughly 6,000 (in highly abundant IT excitatory neurons) to 180 (endothelial cells and vascular leptomeningeal cells (VLMCs)) (Extended Data Fig. 8b), the latter close to the expected false discovery rate. There was modest correlation (Pearson = 0.62) between the number of nuclei in a supertype and the number of genes called significant (Extended Data Fig. 8c). To visualize the complex temporal changes in gene expression, we created a gene-dynamic space encompassing each gene’s mean expression, and earlier and later effect sizes across CPS in all supertypes (Extended Data Fig. 8d–f). This integrated space illustrates both cell-type-selective changes and temporal dynamics common across broader cell subclasses. Supplementary Table 6 contains the gene set enrichments for 31 curated gene sets related to molecular processes implicated in AD. For example, nearly every type of neuron showed decreases along the CPS in the electron transport chain (ETC) and several ribosomal genes (Extended Data Fig. 8g).

### An integrated atlas of community AD data

**Fig. 4:**
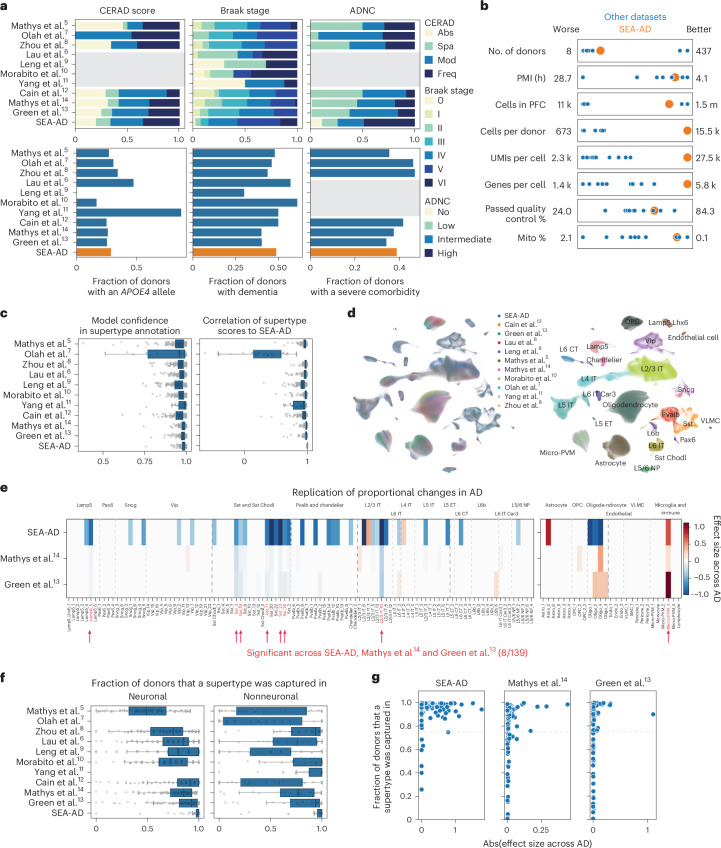
A9 single-nucleus data integration replicates MTG vulnerable populations with AD. **a**, Bar plots showing the fraction of donors in each publicly available snRNA-seq dataset harmonized in this study. Neuropathological stages (top) or possessing an *APOE4* allele, dementia or a severe comorbidity (bottom). Gray boxes, unavailable metadata. Neuropathological staging included CERAD score, Braak stage and ADNC. All datasets applied snRNA-seq to the prefrontal cortex (PFC) in human donors that contained sporadic AD cases. Abs, absent; Spa, sparse; Mod, moderate; Freq, frequent. **b**, Scatter plots showing the relative study size, dataset depth and mean quality control metrics across publicly available snRNA-seq datasets (shown as blue dots) and SEA-AD (shown as a larger orange dot). **c**, Left, box-and-whisker plot showing the mapping confidence across datasets for each supertype. Right, box-and-whisker plot showing the Spearman correlation of each supertype’s signature score across all nuclei in each dataset compared to the SEA-AD. **d**, Scatter plot showing the uniform manifold approximation and projection (UMAP) coordinates computed from the integrated latent representation of cells and nuclei from the SEA-AD snRNA-seq dataset on A9 and each publicly available dataset color-coded according to dataset of origin (left) or subclass (right). **e**, Heatmap comparing the effect size of the relative abundance change of each supertype in A9 across CPS (SEA-AD) or ADNC (refs. ^13^^,^^14^), controlling for sex, age at death and race in the SEA-AD or sex, age and *APOE4* status in refs. ^13^^,^^14^. Red indicates supertypes that were significantly changed in abundance across all three studies. The light gray dashed lines separate subclasses within cellular neighborhood; darker gray lines separate cellular neighborhoods. **f**, Box-and-whisker plots showing the fraction of donors that each supertype was captured in across all 11 integrated datasets. *n* as in **c**. *n* represents the total number of cells in each study dataset ordered as in the figure from top to bottom: 32,312, 11,020, 77,791, 77,631, 25,267, 44,514, 28,064, 89,358, 1,502,282, 1,420,559, 1,330,571. **g**, Scatter plots relating the effect size for each supertype to the fraction of donors for which the supertype was captured in. No populations captured in less than 75% of profiled donors were detected as significant across all studies. The cohort demographics can be found in Supplementary Table 1.

**Extended Data Fig. 9:**
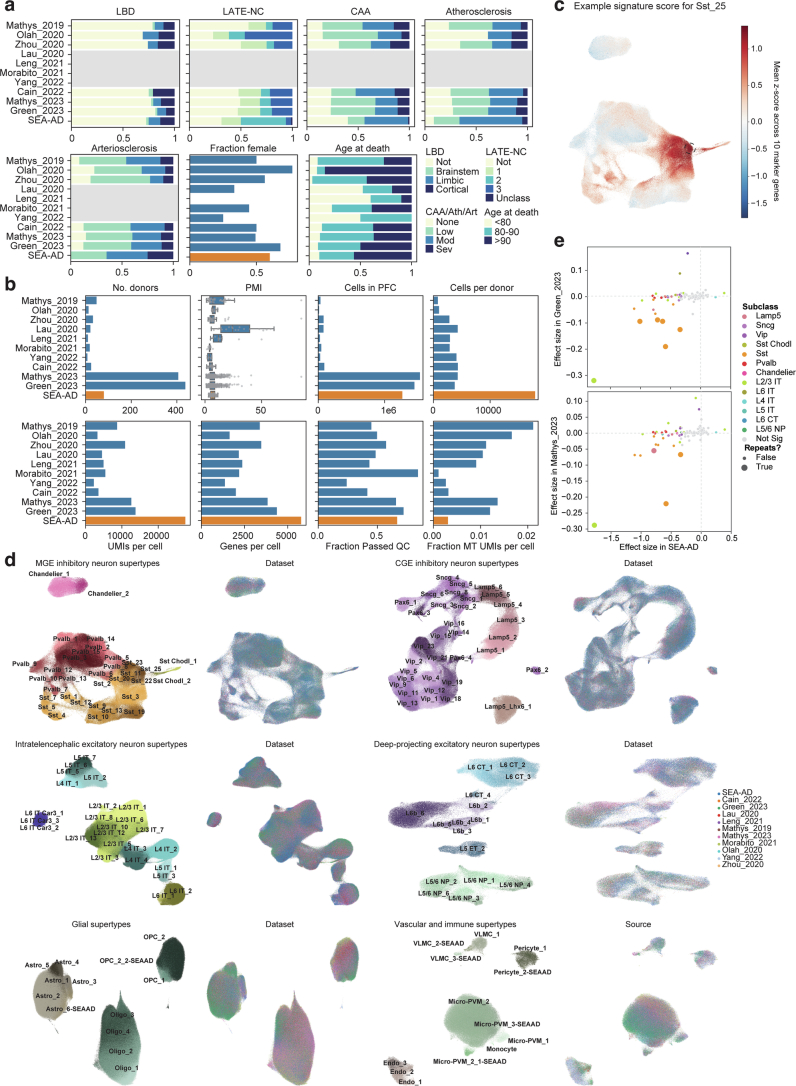
Integration of publicly available snRNA-seq datasets. **a**) Barplots showing the fraction of donors in each of the publicly available snRNA-seq datasets that we harmonized metadata for and integrated classified in co-pathology neuropathological stages (LBD, Lewy Body Disease; LATE-NC, limbic-predominant age-related TDP-43 encephalopathy neuropathologic changes; CAA, Cerebral amyloid angiopathy; Ath, Atherosclerosis; Art, Arteriosclerosis), that were female, or were in defined age groups. Grey boxes, metadata that was unavailable. **b**) Box and whisker or barplots showing quality control metrics across each of the publicly available datasets. Metrics for the SEA-AD A9 snRNA-seq dataset are shown at bottom in orange for comparison. **c**) Scatterplot showing UMAP coordinates for MGE-derived inhibitory interneuron supertypes across all publicly available and the SEA-AD A9 dataset. Nuclei or cells are colored based on the signature score for Sst_25, which are indicated with the black dashed circle. **d**) Scatterplots showing UMAP coordinates of all supertypes within their cellular neighborhoods (that is MGE-derived inhibitory neurons, CGE-derived inhibitory neurons, Intratelencephalic excitatory neurons, Deep-projecting excitatory neurons, glial cells, and vascular and immune cells. In each neighborhood on left are nuclei and cells colored by supertype and on right cells are colored by dataset. **e**) Scatterplots relating the effect size for the change in relative abundance across supertypes in the SEA-AD A9 dataset to those observed in the Green_2023 (top) and Mathys_2023 (bottom) datasets. Each point is a supertype colored by their subclass and supertypes that are significant in both datasets have bigger circles. Dashed grey lines are at 0. Note, several Sst, 1 L2/3 IT and 1 Lamp5 supertypes that have significant negative effect sizes in both datasets. Cohort demographic can be found in Supplementary Table 1.

Previous studies described AD-associated molecular and cellular changes^5–15^; however, cross-study comparisons are challenging without common cell annotations. To corroborate the results, we harmonized snRNA-seq data and associated donor metadata from the PFC from ten additional AD studies spanning 707 donors^5–14^. Cohorts from most studies, including the SEA-AD, spanned the spectrum of plaque and tangle pathology (Fig. 4a, top and Extended Data Fig. 9a), although the SEA-AD contained a greater fraction of donors with neurofibrillary tangle spread into the PFC (Braak stages V and VI). With rare exceptions^9^, the fraction of donors in each cohort with an *APOE4* allele, clinically diagnosed with dementia and with severe comorbidities were similar (Fig. 4a, bottom and Extended Data Fig. 9a). The SEA-AD profiled a relatively large number of donors, number of overall nuclei and number of nuclei per donor, while also having high sequencing depth and gene detection per nucleus, designed to allow highly granular cell type analyses (Fig. 4b and Extended Data Fig. 9b).

All datasets were mapped to the BRAIN Initiative A9 cellular taxonomy using the same hierarchical approach as outlined above; marker-based signature scores were computed for each supertype in each dataset (Extended Data Fig. 9c). Model confidence and supertype signature scores were uniformly high across types (Fig. 4c except for ref. ^7^), allowing construction of an integrated representation across all cells and across cells in each cell type neighborhood (Fig. 4d and Extended Data Fig. 9d). Two studies^13,14^ contained sufficient cells and donors to assess supertype abundance along the ADNC. Eight of 34 supertypes with significant changes in A9 in SEA-AD also changed in these studies (Fig. 4e, Extended Data Fig. 9e and Supplementary Table 4). This included five Sst interneuron, one microglia, one Lamp5 interneuron and one L2/3 IT supertype. Only oligodendrocytes had contradictory significant effect sizes (decreasing in both the MTG and A9 of SEA-AD and increasing in both ref. ^13^ and ref. ^14^) Effect sizes were consistently lower in these datasets, more than could be explained by using ADNC versus CPS alone (compared to Fig. 3b). The difference may relate to both studies having fewer donors with a high Braak stage (V and VI; >70% of their donors would lack pTau tangles in PFC) and to sampling fewer nuclei per donor, which limited the capture of each supertype consistently (roughly 30% of supertypes were missing in at least a quarter of donors compared to only 4% in SEA-AD) (Fig. 4f). Significant changes were only detected in supertypes present in at least 75% of donors in all three studies (Fig. 4g), suggesting that this sparsity was particularly detrimental. Notably, some of the supertypes that were not replicated had nonsignificant effect sizes that were directionally consistent with SEA-AD, such as Sst_20.

### Vulnerable Sst neurons in early AD

**Fig. 5:**
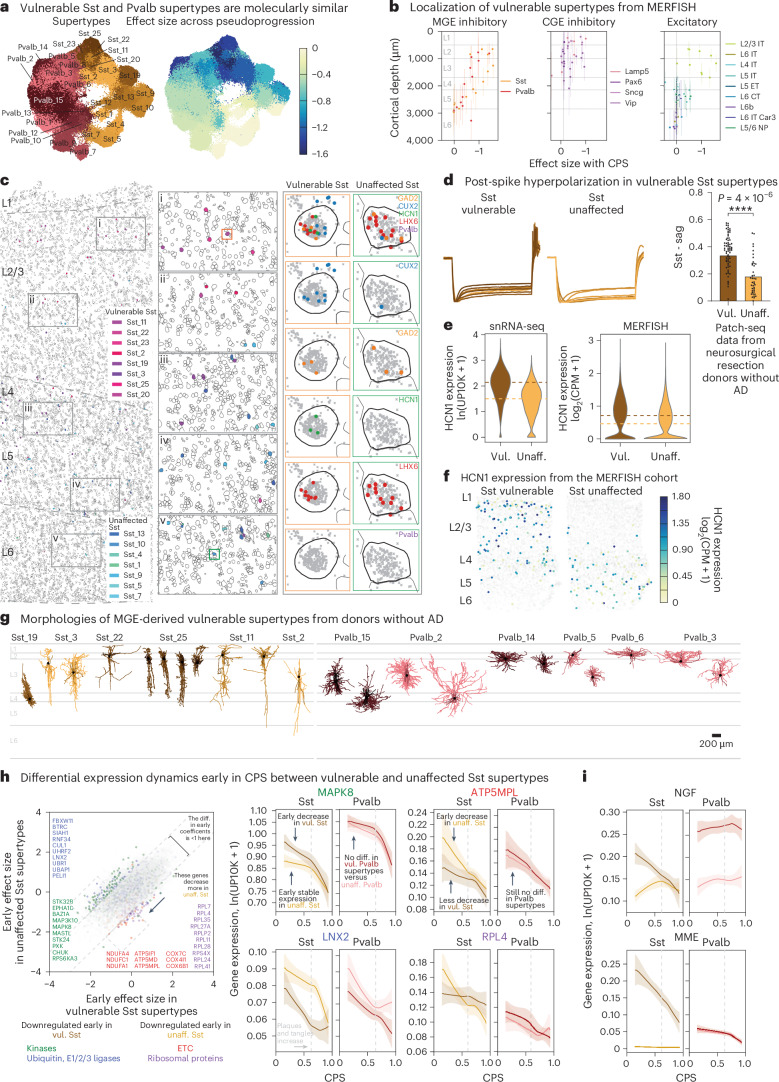
Changes in superficial vulnerable MGE-derived inhibitory interneurons with common electrophysiological feature. **a**, UMAP coordinates for MGE interneurons color-coded according to supertype (left) or the effect size of the relative changes in abundance from scCODA along the CPS (right). **b**, Scatter plots relating the effect size of the changes in abundance to the cortical depth for each neuronal supertype. Each point indicates the MERFISH-derived mean depth of the supertype; the error bars indicate the s.d. *n* represents the total number of MERFISH cells with quantified cortical depth (*n* = 349,941). **c**, Example MERFISH data from early CPS (0.23), with cell locations and boundaries. Cortical layers are separated by the dashed gray lines. Vulnerable Sst neurons are indicated by pink-purple hues; unaffected neurons are indicated by green-blue hues. **d**, Left, electrophysiological traces showing post-spike membrane potential hyperpolarization over time (*y* axis) in vulnerable Sst neurons recorded from human donors without AD. Right, bar and swarm plot indicating the Sag distributions. A logistic regression test was used to identify the differential electrophysiological features (*P* = 4 × 10^−6^). The *P* values for the differential intrinsic features are shown in Supplementary Table 8. *n* represents the total number of Sst cells profiled using patch-seq (*n* = 209). **e**, Violin plots of HCN1 expression in Sst neurons in snRNA-seq (left) and MERFISH (right). The colored dashed lines represent the mean expression. ln(UP10K + 1), natural log of UMIs/10,000 + 1. log_2_(counts per million (CPM) + 1). The statistical test was a negative binomial regression implemented in Nebula as described in the Methods. **f**, Scatter plot of Sst cells indicating cell position and HCN1 expression level in an early CPS donor (0.23). Superficial Sst cells have higher HCN1 expression. **g**, Patch-seq-derived morphological reconstructions of vulnerable MGE-derived interneurons from donors without AD. Dendrites are colored according to supertype. **h**, Scatter plot relating the mean early effect size for genes in vulnerable versus unaffected Sst supertypes. Gene families with decreased expression in vulnerable types are shown in blue (ubiquitin ligases, *P* = 0.036) and green (kinases, *P* = 8.92 × 10^−11^). Gene families with decreased expression in unaffected types are shown in red (ETC, *P* value near 0) and purple (ribosomal proteins, *P* value near 0). The statistical test is a negative binomial regression implemented in Nebula and gene family enrichment tests as described in the Methods and Supplementary Note. Right, LOESS regression plots of mean gene expression for vulnerable (dark orange) and unaffected (light orange) Sst types and vulnerable (dark red) and unaffected (light red) Pvalb types. The center lines are the mean from the LOESS fits; uncertainly, lines represent the s.e. from 1,000 LOESS fits with 80% of the data randomly selected in each iteration. ln(UP10K + 1), natural log UMIs/10,000 + 1. **i**, LOESS regression plots as in **h**. *NGF* and *MME* gene expression decreased in vulnerable Sst supertypes. Center lines and error bars as in **h**. The cohort demographics can be found in Supplementary Table 1. **g**, Scale bar, 200 μm. Diff., difference; unaff., unaffected; vul., vulnerable.

**Extended Data Fig. 10:**
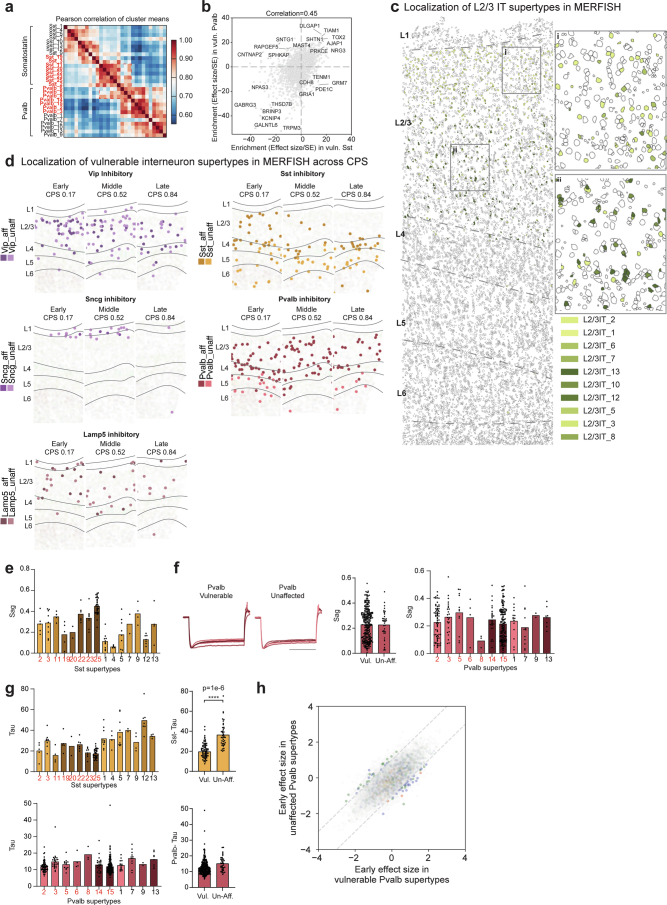
Characteristics of vulnerable neuronal supertypes. **a**) Heatmap showing the pairwise correlations of the mean expression of all genes across the MGE-derived supertypes indicated. Red labels are vulnerable supertypes. **b**) Scatterplot relating the mean enrichment (defined as the effect size divided by its standard error (SE) from NEBULA) of each gene in vulnerable (vuln) Sst and Pvalb supertypes compared to unaffected types in their respective subclasses. **c**) MERFISH-profiled brain slice in early CPS donor (CPS = 0.23) showing each cells location and boundaries defined by the cell segmentation, with cortical layers indicated (L1-L6) and separated by dashed grey lines. Vulnerable L2/3 intratelencephalic (IT) neurons are color-coded. Insets: i) L2/3 IT supertypes have characteristic depths within layers 2 and 3. **d**) Scatterplots showing the spatial locations of individual cells of the inhibitory neuron subclasses indicated from representative MERFISH sections in donors at increasing CPS stages. Vulnerable supertypes (aff) are shown in darker colors and unaffected supertypes (unaff) in lighter ones. **e**) Bar and swarm plot showing the Sag values for Sst supertypes from PatchSeq data on non-AD donors. Vulnerable supertypes are colored in red. **f**) Left, electrophysiological traces showing post-spike hyperpolarization of membrane potential (y-axis) over time in almost all Pvalb neurons from tissue of non-AD human donors that underwent surgical resection. Middle, bar and swarm plot showing Sag distributions in individual vulnerable (Vul) and unaffected (Unaff) Pvalb neurons. Right, Bar and swarm plot showing the Sag values for Pvalb supertypes from PatchSeq data on non-AD donors. Vulnerable supertypes are colored in red. **g**) Left top and bottom, Bar and swarm plot showing the Tau apparent membrane time constant values for Sst (top) and Pvalb (bottom) supertypes from PatchSeq data on non-AD donors. Vulnerable supertypes are colored in red. Middle top and bottom, Bar and swarm plots for data on left grouped by vulnerable (vul) and unaffected (un-aff) Sst (top) and Pvalb (bottom) supertypes. Logistic regression test is described in ‘Identifying differential electrophysiological features’, p-value = 1e-6. P-values for all differential electrophysiological features are in Supplementary Table 8. **h**) Scatterplot relating the mean early effect size of each gene (dots) in vulnerable versus unaffected Pvalb supertypes. Cohort demographic can be found in Supplementary Table 1.

Nearly all vulnerable neuron supertypes were located in the upper (supragranular) cortical layers. Spatial transcriptomics revealed that vulnerable supertypes from MGE-derived Sst^+^ and Pvalb^+^ subclasses are localized primarily to supragranular layers 2 and 3 (Fig. 5b,c). Other vulnerable neurons, including all CGE-derived interneurons (for example, Lamp5^+^, Vip^+^, Sncg^+^ and Pax6^+^ neurons) and L2/3 IT neurons were also only found in upper, supragranular layers (Fig. 5b and Extended Data Fig. 10c,d). Sst^+^ and Pvalb^+^ interneuron subclasses form a transcriptional continuum (Fig. 5a, left); vulnerable Sst and Pvalb supertypes were as transcriptionally similar to each other as they were to other, unaffected supertypes within their respective subclasses (Fig. 5a, right and Extended Data Fig. 10a). Interestingly, hundreds of genes were selectively expressed in both vulnerable Sst and Pvalb supertypes, but not in unaffected supertypes from these subclasses (Extended Data Fig. 10b and Supplementary Table 7).

The typical morphoelectrical properties (that is, before disease onset) of the MGE supertypes were recently characterized using patch-seq profiling in neurosurgically resected tissues from human donors without AD^21,22^; the supragranular localization of vulnerable Sst and Pvalb interneurons was qualitatively confirmed. Vulnerable Sst supertypes had higher post-spike hyperpolarization (Sag) and lower membrane polarization time constants (Tau) compared to unaffected Sst supertypes (Fig. 5d, Extended Data Fig. 10e and Supplementary Table 8), differences not seen between vulnerable and unaffected Pvalb supertypes (Extended Data Fig. 10f,g). Hyperpolarization-activated cyclic nucleotide-gated 1 (HCN1) expression (HCN channel activity is involved in setting the Sag level) was higher in vulnerable Sst supertypes in both snRNA-seq and MERFISH in donors with AD (Fig. 5c,e,f). The vulnerable supertypes spanned a wide morphological range that includes non-Martinotti, sparse, basket, basket-like and double bouquet cells (Fig. 5g).

Vulnerable Sst supertypes showed specific molecular changes with AD. Unlike all other neuronal supertypes, they did not downregulate components of the ETC (Fig. 5h, red) and ribosomal genes (Fig. 5h, purple). Compared to unaffected Sst supertypes, the vulnerable Sst supertypes collectively downregulated specific kinases (from the tyrosine kinase^56^ and calcium^2+^/calmodulin-dependent kinase^57^ families) (Fig. 5h, green) and E3 ubiquitin ligases (from the homologous to the E6-AP carboxyl terminus (HECT) family^58^) (Fig. 5h, blue). In contrast, vulnerable and unaffected Pvalb supertypes had no gene families affected differentially between them early in CPS (Extended Data Fig. 10h). Several notable genes were sharply downregulated early in CPS specifically in vulnerable Sst supertypes, including nerve growth factor (NGF) and genome-wide association study hit membrane metalloendopeptidase (MME) (Fig. 5i). The cognate receptor for NGF and NGFR is expressed specifically in oligodendrocytes and OPCs, suggesting potential disruption in communication with vulnerable Sst supertypes that may impact myelination^59^.

**Fig. 6:**
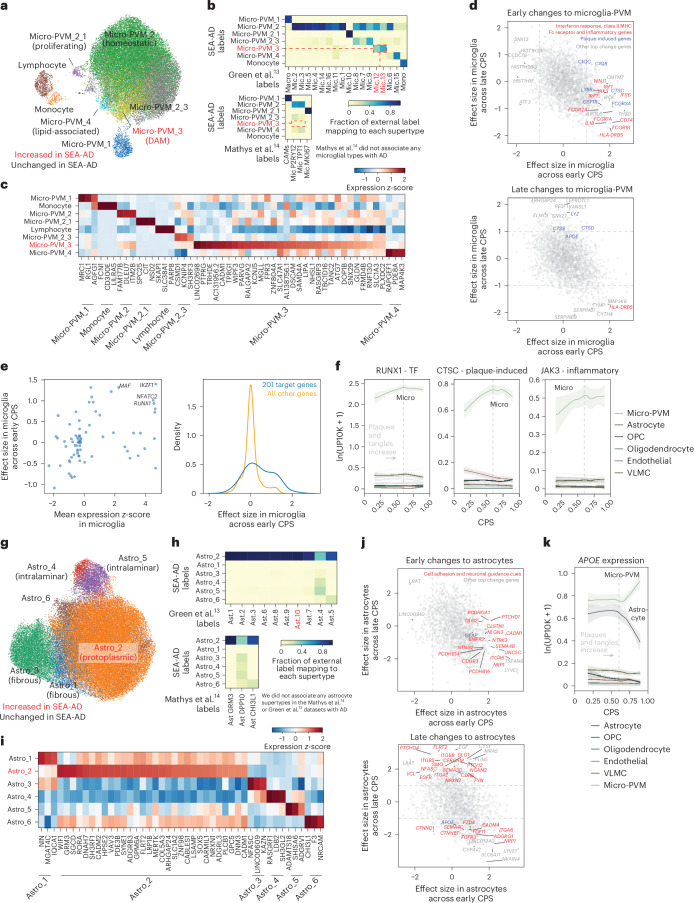
Early microglial and astrocyte activation compared across publicly available datasets. **a**, Scatter plot showing the UMAP coordinates for MTG micro-PVM supertypes, colored according to supertype identity. Red, disease-associated microglial state. **b**, Heatmaps showing confusion matrices comparing microglial annotations in refs. ^13,14^ with the SEA-AD cellular taxonomy. Red, SEA-AD supertypes significantly increased in all datasets. **c**, Heatmap showing the mean *z*-scored expression across microglial supertypes of marker genes identified using Nebula. **d**, Scatter plot relating the mean effect size of each gene across microglial supertypes in the early versus late epochs along the CPS. The gray dashed lines denote effect sizes of 1 and −1. The statistical test was a negative binomial regression implemented in Nebula, together with gene family enrichment tests as described in the Methods and Supplementary Note. **e**, Left, scatter plot relating transcription factor mean *z*-scored gene expression identified by the GRNs versus the effect size in the early disease epoch along the CPS. Right, cumulative density plot depicting the effect sizes in the early disease epoch along the CPS of the genes downstream of the transcription factors identified based on the GRNs (left, in blue) versus the effect sizes of all other genes (yellow). **f**, LOESS regression plots relating the mean expression of the indicated genes from families noted in **d** to CPS across nonneuronal supertypes organized and colored according to subclass; ln(UP10K + 1), natural log UMIs per 10,000 + 1. **g**, Scatter plot showing the UMAP for the MTG astrocyte supertypes colored according to supertype identity. Red, disease-associated protoplasmic astrocyte supertype. **h**, Heatmaps showing the confusion matrices comparing the annotations of astrocyte cells in the studies in refs. ^13,14^, with the same cells annotated with the SEA-AD cellular taxonomy. **i**, Heatmap showing the mean *z*-scored expression across astrocyte supertypes of marker genes identified by Nebula. **j**, Scatter plot relating the mean effect size of each gene across astrocyte supertypes in the early (*x* axis) versus late (*y* axis) epochs along the CPS. The gray dashed lines denote effect sizes of 1 and −1. **k**, Same LOESS regression plots as in **f** for the strongly disease-associated *APOE* gene, which decreased in expression in astrocytes and increased in expression in microglia in the late disease epoch along the CPS. The cohort demographics can be found in Supplementary Table 1. TF, transcription factor.

### Microglia and astrocyte activation in early AD

Several cellular taxonomies for myeloid immune cells in the brain have been proposed using snRNA-seq data from healthy and diseased humans^7,12,13^. These taxonomies generally agree on three major types of brain myeloid lineage cells: monocytes, central nervous system (CNS)-associated macrophages (CAMs)/perivascular macrophages (PVMs) and a heterogenous group of microglia that has been difficult to reconcile across taxonomies. Comparing the SEA-AD microglial taxonomy (Fig. 6a) to the highly diverse one from ref. ^13^, revealed strong agreement across studies (Fig. 6b, top). Most notably, both taxonomies contained disease-associated types (micro-PVM_3 in SEA-AD and Mic.12 and Mic.13 in ref. ^13^), which were consistently increased in abundance with AD across datasets and have a common molecular signature (Fig. 6c). Also, both studies identified homeostatic, proliferative and lipid-associated types^60^ (micro-PVM_4 in SEA-AD and Mic.6 and Mic.15 in ref. ^13^), and one other subtype with no functional data or tissue localization information. While the SEA-AD taxonomy is more conservative in splitting subtypes (with many one-to-many relationships to those with ref. ^13^), the same transcriptional continuum is captured in both datasets. Mathys et al.^14^ were even more conservative, describing only homeostatic and proliferative subtypes, despite their datasets containing other subtypes (Fig. 6b, bottom), such as a disease-associated microglia (DAM) subtype.

In addition to confirming the existence of DAMs in the SEA-AD dataset, broader molecular changes in microglia along the CPS were consistent with previous studies. Early changes included significant upregulation of gene sets involved in inflammatory processes (*IL1B*, *CSF1R*, *STAB1*, *NINJ1*, *JAK3*)^61–65^, interferon response (*IRF1*, *IRF7*, *IFI16*), Fc receptors (*FCGR1A*, *FCGR1B*, *FCGR2A*, *FCGR3B*), components of the class II major histocompatibility complex (MHC) (*CD74*, *HLA-DRB5*) and complement components (*C1QA*, *C1QB*) (Fig. 6d, top, red). Surprisingly, we also observed early upregulation of several homologs of genes induced by Aβ plaques in AD (*CSF1R*, *CTSC*, *C1QA*, *C1QB*, *LY86*, *FCGR3A*)^66^ (Fig. 6d, top, blue). Other plaque-induced genes were upregulated later in CPS in donors with higher levels of pathology (Fig. 6d, bottom, blue), including more cathepsins (*CTSD* and *CTSS*) that may facilitate Aβ clearance^67^, the gene encoding lysozyme (*LYZ*) and *APOE*, which is by far the most strongly associated genetic risk factor for AD^68^. To identify the transcription factors driving early upregulation of proinflammatory and plaque-induced genes, we used snATAC–seq data to construct microglial gene regulatory networks (GRNs). Four transcription factors (RUNX1, IKZF1, NFATC2, MAF) showed specific microglial expression and were upregulated early in CPS (Fig. 6e, left). These transcription factors are predicted to coregulate 201 genes, including genes noted above (Fig. 6e, right and Fig. 6f).

Astrocytes have been ascribed diverse roles in AD pathophysiology^69^, which makes understanding their molecular subtypes crucial. The SEA-AD taxonomy encompasses interlaminar, protoplasmic, fibrous and a yet-to-be-described astrocyte supertype (Fig. 6g). In contrast (Fig. 6h, top), Green et al.^13^ split protoplasmic astrocytes into several subtypes, grouped interlaminar astrocytes into one subtype and found few fibrous astrocytes (Fig. 6i). In both MTG and A9 datasets, protoplasmic astrocytes (Astro_2) specifically increased early in CPS. While this association could not be replicated in ref. ^13^ or ref. ^14^, their original manuscript noted an increase in one protoplasmic subtype (Ast.10) with AD. This suggests agreement that at least a subset of astrocytes is increased with disease. Mathys et al.^14^ had the fewest types, with one subtype for protoplasmic astrocytes, one subtype for fibrous and interlaminar astrocytes together, and one unknown subtype that was also similar to a type we identified (Fig. 6h, bottom).

Next, we sought to describe the early and late molecular changes occurring in astrocyte supertypes. Early changes included upregulation of cellular adhesion molecules (CADM1, CDRH3, PCDHGA1, PCDHB14, PCDHB16, CLSTN1, ITGA6, NEO1, ANOS1) and neuronal guidance cues (NLGN3, NTRK3, SEMA4B, NTNG2), signaling receptors (PTCHD1, NRP1, BMPR2, UNC5C)^70^ and GFAP, a known hallmark of AD and astrogliosis^71^ (Fig. 6j, top). Later in CPS, astrocytes continue to upregulate molecules involved in cellular adhesion, axonal guidance and signaling receptors, including NCAM2 and CERCAM, additional hedgehog signaling receptors (PTCHD4, PTCH2, SMO) and their downstream target transcription factor GLI1, and both the epidermal growth factor ligand and its receptor (Fig. 6j, bottom). Astrocytes also downregulated APOE (Fig. 6k). Collectively, these molecular changes suggest a highly stimulatory extracellular environment occurring early in disease, even in donors with relatively low levels of pathology.

### Oligodendrocyte loss and remyelination by OPCs

**Fig. 7:**
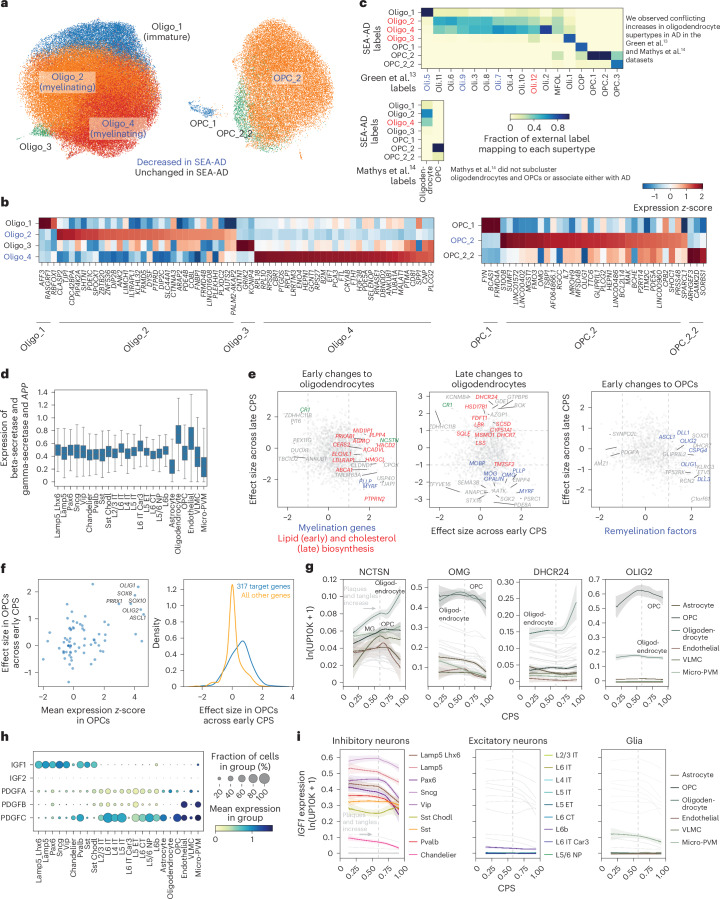
Early loss of oligodendrocytes with a remyelination program in OPCs across publicly available datasets. **a**, Scatter plots showing the UMAP coordinates for MTG oligodendrocyte and OPC supertypes, colored according to supertype identity. **b**, Heatmap showing the mean *z*-scored expression across oligodendrocyte (left) and OPC (right) supertypes of marker genes identified by Nebula. **c**, Heatmaps showing confusion matrices comparing oligodendrocyte and OPC annotations in refs. ^13,14^ with the SEA-AD taxonomy. Red, SEA-AD supertypes that were significantly increased in AD in these datasets. Red and blue also denote cell types that were associated or vulnerable with disease in the original studies. **d**, Box-and-whisker plot showing the mean expression (natural log UMIs per 10,000 + 1) of beta and gamma-secretase components and the *APP* gene organized according to subclass. The center lines denote the median; the error bars are 1.5 times the IQR. Outliers are not shown. **e**, Scatter plot relating the mean effect size of genes across the oligodendrocyte and OPC supertypes in the early versus late epochs. Significant genes involved in fatty acid biosynthesis (left) or cholesterol biosynthesis (middle, *P* = 0.0040 late) are color-coded red; myelin components (*P* = 0.006 late) are color-coded blue. Significant genes in the OPC early phase (right) that are part of the remyelination program (*P* = 9.62 × 10^−5^ early) are color-coded blue. The statistical test used was a negative binomial regression implemented in Nebula; gene family enrichment tests were carried out as described in the Methods and Supplementary Note. **f**, Left, scatter plot relating transcription factor mean *z*-scored gene expression identified by GRNs versus their effect size in the early disease epoch along the CPS. Right, cumulative density plot depicting the effect sizes in the early disease epoch of genes downstream of the transcription factors identified (left) based on the GRNs (blue) versus the effect sizes all other genes (yellow). *n* represents the number of OPCs, *n* = 28,429. **g**, LOESS regression plots relating the mean expression of the indicated genes from the families in **e** to the CPS, colored according to subclass. ln(UP10K + 1), natural log UMIs per 10,000 + 1. **h**, Dot plot depicting the mean gene expression and fraction of cells in each group with nonzero expression in the SEA-AD MTG dataset organized according to the subclasses for the genes indicated. Expression is natural log UMIs per 10,000 + 1. The statistical test was negative binomial regression implemented in Nebula; gene family enrichment tests were used as described in the Methods and Supplementary Note. **i**, LOESS regression relating the mean expression of *IGF1* to CPS, color-coded by inhibitory (left), excitatory (middle) and nonneuronal (right) subclasses. The cohort demographics can be found in Supplementary Table 1.

Dysfunction of oligodendrocytes and myelin breakdown may be early events in AD^72–75^. Among oligodendrocytes, two supertypes (Oligo_2 and Oligo_4) were vulnerable early in both MTG and A9 (Fig. 7a); both supertypes are found throughout the cortical column in the BRAIN Initiative reference dataset^18^. *CNP* was expressed in both (albeit higher in Oligo_4) (Fig. 7b), suggesting they are myelinating oligodendrocytes. We also observed a late decrease in one OPC supertype (OPC_2), which is found across cortical layers 2 through 6. When compared against publicly available datasets, SEA-AD oligodendrocytes and OPCs largely agreed with the fine-grained types described in ref. ^13^, with most supertypes having one-to-one or one-to-many relationships (Fig. 7c).

The mean expression of genes implicated in Aβ synthesis in oligodendrocytes (*BACE1*, *BACE2*, *PSEN1*, *PSEN2*, *APH1A*, *NCSTN*^15^) was replicated in the SEA-AD data (Fig. 7d), with oligodendrocytes having the highest levels of both *APP* and *PSEN1*. Therefore, the early loss of oligodendrocytes may be attributed to these higher levels of Aβ molecules that have known cytotoxicity. Additionally, there is an early upregulation of a gamma-secretase component (*NCSTN*), the transcription factor MYRF, which regulates myelination^76^, and a structural component of myelin itself (PLLP) (Fig. 7e, left, and Fig. 7g). Significant increases in expression of the cholesterol biosynthesis gene family, a proposed key process in AD etiology^77^, occur later in CPS (*DHCR24*, *LBR*, *FDFT*, *HSD17B1*, *SC5D*, *CYP51A1*, *SQLE*, and *DHCR7*) (Fig. 7e, middle, and Fig. 7g). Furthermore, late in CPS there is downregulation of *MYRF* and several components of myelin and myelination (*MOBP*, *MOG*, *OMG*, *PLLP*, *OPALIN*). The late change in both gene sets suggests that they may represent a reaction to pathology rather than an early driver of dysfunction.

In OPCs, there was early upregulation of several transcription factors (OLIG1, OLIG2, SOX10, SOX8, PRRX1, ASCL1) and Notch ligands (DLL1, DLL3) known to regulate differentiation^78–81^ to oligodendrocytes after loss of surrounding oligodendrocytes (Fig. 7e, right, and Fig. 7g). Because of the overwhelming number of transcription factors involved in differentiation that changed early, we queried our OPC-specific GRN and identified 317 genes downstream of these factors (Fig. 7f, left). These genes were also upregulated early (Fig. 7f, right) compared to all other genes, and were predominantly involved in OPC differentiation. Next, we examined the expression of two signaling pathways that are important for OPC differentiation to oligodendrocytes: insulin-like growth factor (IGF)^82^ and platelet-derived growth factor (PDGF)^83^. While expression of *PDGF* genes spanned several cellular subclasses, expression of *IGF* was restricted to inhibitory interneurons and a small subset of microglia (Fig. 7h). *IGF1* expression decreased later in CPS in several inhibitory interneuron populations, suggesting that these inhibitory populations may be the main source of IGF1 and the driver of changes in myelination (Fig. 7i).

## Discussion

We describe an integrated atlas of AD in the MTG, selected both as a transition area in AD pathology^4^ and the region with the greatest aggregated knowledge about cell type phenotypes^20–22^. The atlas illustrates the utility of the BICCN reference as a unifying framework to map cell types at high resolution, incorporate cell types and states not included in the reference, and replicate results. The core results presented in this article were replicated across data modalities, cortical regions and datasets from independent studies. The results demonstrate the value of this integration in defining a robust and specific series of cellular and molecular events that show what cells are affected, where they are (co)localized and when these events happen as disease pathology increases. All data presented are publicly accessible through a suite of data resources available through SEA-AD (https://portal.brain-map.org/explore/seattle-alzheimers-disease).

**Fig. 8:**
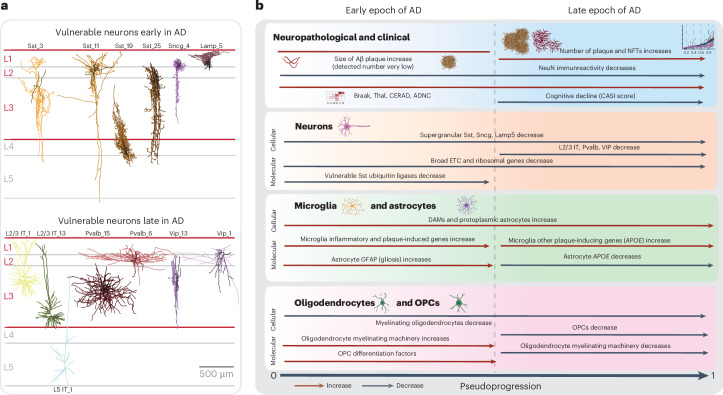
MTG cells impacted by AD, predominantly localizing to superficial layers, can be organized into two epochs: an early and a late phase. **a**, Diagram illustrating cortical columns with actual neuronal reconstruction from vulnerable populations (from donors without AD) organized according to early (top) and late (bottom) disease epochs. During the early epoch, superficial Sst, Sncg and Lamp5 interneurons were lost. In the late epoch, most lost neurons localized superficially (L2/3 IT, Pvalb and Vip), with the addition of deep cortical and striatum-projecting L5 IT neurons. **b**, First box, the dynamic changes associated with AD progression can be organized into early and late epochs. In the early epoch, the first neuropathological event is an increase in the size of sparse Aβ plaques, subsequently followed by an exponential aggregation of both pTau and plaque burden. A decrease in NeuN^+^ cells occurs throughout. Second box, supragranular interneurons (Sst, Sncg, Lamp5) are lost early on. During this period, genes encoding the ETC complex, and ribosomal proteins, are downregulated broadly across neurons, except in the vulnerable Sst interneurons. In the latter cells, there is a strong downregulation of ubiquitin ligases and kinases. Later on, not only inhibitory cells (Pvalb and Vip) are lost but also long-range-projecting pyramidal neurons (L2/3 IT and L5 IT). Third and fourth boxes, nonneuronal cells accompany these changes with the early emergence of DAMs and an increase in protoplasmic astrocytes, while myelinating oligodendrocytes decrease their abundance. Concurrently, DAMs upregulate inflammatory and plaque-inducing genes, while OPCs attempt to compensate for oligodendrocyte loss by upregulating their OPC differentiating genes. Later, OPC cells are impacted and lost while myelination genes in oligodendrocytes are downregulated. The cohort demographics can be found in Supplementary Table 1. Schematics created using Biorender.com.

Modeling disease severity using pseudotrajectory analyses based on a quantitative local neuropathological burden was highly successful and increased effect sizes beyond aggregate scores like Braak^4^, Thal^3^, CERAD^84^ and ADNC^39^. These measure distribution of pTau, aβ and neuritic plaques, but rely on binary present or absent scores that do not capture the level of pathology in any given brain region. This pseudotrajectory was driven by AD phenotypes and captured two major epochs in AD progression (Fig. 8), including an early phase with slowly increasing neuropathology and a late phase with exponentially increasing neuropathology, culminating in the terminal state observed for severely affected donors. In the early epoch, donors had sparse Aβ plaques (albeit increasing in size) and pTau^+^ tangle-bearing neurons, accompanied by early increases in inflammatory or reactive microglial^7^ and astrocytic states^69^ and associated gene expression changes in relevant inflammatory^85^ and plaque-induced genes (Fig. 8b). This epoch also features losses of oligodendrocytes and a dramatic increase in OPC differentiation and remyelination factors that may represent a compensatory response like that seen in models of oligodendrocyte loss^86,87^. Neuronal cells exhibited loss of particular Sst interneuron types that downregulate kinases and E3 ubiquitin ligases, but not the ETC and ribosomal pathways (which were downregulated in other neuronal populations) (Fig. 8b). These vulnerable Sst supertypes were localized to superficial cortical layers, whereas deeper-layer Sst supertypes were unaffected (Fig. 8a), and exhibited distinctive electrophysiological properties, such as higher Sag, compared to unaffected supertypes. These neurons are lost earlier than L2/3 IT excitatory types, which bear the highest Tau burden in the cortex^46^, suggesting high pathological susceptibility and an initial event of circuit dysfunction. Sst interneurons have been implicated in AD^53,55^, but not at the same molecular, morphological and electrophysiological level, which was achieved, in part, by our neuronal enrichment strategy (harnessing fluorescence-activated nuclei sorting (FANS) to enrich neuronal populations). Severely affected donors exhibited a decrease in NeuN immunostaining, raising some concern that the use of NeuN as a neuronal marker in our FANS protocol could have biased cellular proportions across donors as a function of AD pathology if some neuronal types lost NeuN labeling disproportionately. However, this is unlikely as our main findings were replicated in an additional cortical region, an orthogonal technology (MERFISH) and in publicly available datasets that did not enrich for neurons^13,14^.

What might be the consequences of an early loss of Sst neurons? Loss of inhibitory neurons would naturally be expected to disrupt excitatory and inhibitory balance; impaired inhibition may therefore increase the susceptibility of patients with AD to epilepsy, a clinical symptom found in more than 10% of patients^88^. This is supported by previous observations highlighting an antiepileptic role of Sst^+^ interneurons^89^, and our observation that susceptible inhibitory interneurons express a high level of the HCN1 channel, whose dysfunction has been linked to several epileptogenesis pathways and the generation of hyperexcitability^90^. From a circuit perspective, Sst interneurons are uniquely positioned to exert control over both excitation and inhibition in the cortex as they target excitatory and all other subclasses of inhibitory cortical neurons, but not themselves^91^. They also participate in a powerful disinhibitory loop via reciprocal connections with the Vip subclass^92^. Furthermore, they mediate the effects of arousal in cortical circuits^92^ under the effects of acetylcholine, which is also highly disrupted early in AD through the loss of cholinergic neurons in the basal forebrain^93^. Thus, reduction in numbers of Sst neurons probably has wide-ranging consequences beyond reduced network stability, affecting cognitive processes that rely on proper interactions in distributed brain areas. Finally, it is possible that Sst neuron loss could also disrupt trophic support of connected neurons^94^, ultimately leading to the loss of long-range corticocortical connectivity that would be expected to affect cognitive function.

In the later epoch, there is an exponential rise in Aβ and pTau pathology, continued increases in inflammatory microglia and astrocyte states, and a decrease in the expression of both the OPC differentiation program and oligodendrocyte expression of myelin-associated proteins (previously characterized using quantitative PCR^95^). There is also broader loss of excitatory (L2/3 IT) and inhibitory (Pvalb and Vip) neurons (Fig. 8b). Vulnerable neuron types are specific, including a subset of the supertypes within each broader subclass, and are largely localized to the upper layers of the cortex (Fig. 8a). For example, there was a selective loss of excitatory neurons in the supragranular layers (L2/3 IT)^9,54^.

Putting these two epochs together, the overall progression suggests a sequence of events in which early microglial activation at low levels of pathology triggers reactive astrocytes and potentially oligodendrocyte loss^96^. Furthermore, the early loss of Sst neurons in the upper cortical layers could lead to excitatory and inhibitory circuit imbalance (Fig. 8b) that could in turn lead to loss of other colocalized (and thus probably connected) excitatory and inhibitory neurons, including long-range corticocortical (L2/3 IT) neurons that contribute to cognitive decline. Donors with the steepest memory cognitive decline late in life showed particularly broad cellular dysfunction, suggesting that this was not due to poor quality samples but rather a biological outcome of AD pathology and subsequent cognitive decline. These severely affected donors had lower transcription and reduced chromatin accessibility that may correspond to senescent states^97^, or global epigenome dysregulation that is indicative of cell identity loss^98^.

The results presented in this study in the MTG demonstrate that systematic application of single-cell genomic and spatial technologies coupled with quantitative neuropathology can effectively model disease progression across the spectrum of AD severity. Importantly, the BICCN reference allows the integration and direct comparison across many studies to use common annotation of the same cell types and states, and to cross-validate results to demonstrate their robustness and consistency.

## Methods

### SEA-AD cohort selection

Postmortem brain tissue and donor metadata were obtained via the UW BioRepository and Integrated Neuropathology (BRaIN) laboratory from participants in the Kaiser Permanente Washington Health Research Institute ACT Study and the University of Washington ADRC. In compliance with all ethical standards, informed consent for research brain donation was obtained according to protocols approved by the UW and Kaiser Permanente Washington Health Research Institute Institutional Review Boards. ACT participants receive compensation for parking and transportation and an incentive of US$50 after completing each study visit. Work at the Allen Institute received a regulatory determination of non-human subject research. The study cohort was selected based solely on donor brains undergoing precision rapid procedure (optimized tissue collection, slicing and freezing) during an inclusion time period at the start of the SEA-AD study, excluding those with a diagnosis of frontotemporal lobar degeneration, Down syndrome, amyotrophic lateral sclerosis or other confounding degenerative disorder (not including Lewy body disease, limbic-predominant TDP-43 encephalopathy or microvascular brain injury). The cohort was chosen in this manner to represent the full spectrum of AD neuropathology, with or without common comorbid age-related pathologies. No randomization was used in cohort selection. Unless otherwise specified, the experimental donor cohort contains 84 donors, 51 females and 33 males, aged 65–102 (mean 88). See Supplementary Table 1 for a breakdown of the specific donors included in each experiment.

### Single and duplex IHC for quantitative neuropathology

The STG-MTG tissue blocks were sectioned (cut at 5 µm), deparaffinized by immersion in xylene for 3 min three times. Then, they were rehydrated in graded ethanol (100%, 3×, 96%, 70% and 50% for 3 min each) and washed with Tris-buffered saline with 0.25% Tween-20 (TBST) twice for 3 min. The slides were immersed in Diva Decloaker 10X solution (cat. no. DV2004, Biocare Medical) for heat-induced epitope retrieval using the Decloaking Chamber at 110 °C for 15 min for most of the antibodies. To detect the α-synuclein protein, enzymatic antigen retrieval with protein kinase (cat. no. AR551, Leica Biosystems) was used. After heat-induced epitope retrieval was completed, the slides were cooled for 20 min at room temperature. Afterward, slides were washed with TBST for 5 min twice.

Chromogenic staining was performed using the fully automated intelliPATH (Biocare Medical). Blocking with 3% hydrogen peroxide (cat. no. PX968, Biocare Medical), Bloxall (Vector Laboratories), Background Punisher (Biocare Medical) and levamisole (Vector Laboratories) was performed to avoid any cross-reactivity and background. The following primary antibodies were used for the first target protein at the dilutions indicated: NeuN (1:500, clone A60, mouse, MAB5374, Merck Millipore), pTDP-43 (1:1,000, Ser409/410, clone ID3, rat, cat. no. 829901, BioLegend), β-amyloid (1:1,000, clone 6E10, mouse, cat. no. 803003, BioLegend), α-synuclein (1:200, clone LB509, mouse, cat. no. 180215, Invitrogen) and GFAP (1:1,000, rabbit, cat. no. Z033401-2, DAKO). After incubation with primary antibodies, sections were washed four times for 2 min with TBST and stained with species-appropriate secondary probe or antibody with a polymer horseradish peroxidase (HRP) (manufacturer’s proprietary dilution, MACH 3 mouse (cat. no. M3M530) and MACH 3 rabbit (cat. no. M3R531), Biocare Medical; manufacturer’s proprietary dilution, ImmPRESS goat anti-rat IgG (cat. no. MP-7444), Vector Laboratories). Sections were washed two times for 2 min with TBST; the antibody complex was visualized after 3–7 min by HRP-mediated oxidation of 3,3′-diaminobenzidine (DAB) (intelliPATH, cat. no. IPK5010) by HRP (brown precipitate). Counterstaining was done with hematoxylin after the DAB reaction.

In duplex IHC (6E10/IBA1 and AT8/pTDP-43), slides were washed for 22 min in TBST and then incubated with primary antibodies at the dilutions indicated after the DAB reaction: IBA1 (1:1,000, rabbit, cat. no. 019-19741, Wako) and pTau (1:1,000, clone AT8, mouse, cat. no. MN1020, Thermo Fisher Scientific). They were washed as above and stained with species-appropriate secondary polymers conjugated to an alkaline phosphatase (MACH 3 mouse (cat. no. M3R532), MACH 3 rabbit (cat. no. M3R533), Biocare Medical). The complex was then visualized with the intelliPATH Ferangi Blue Chromogen Kit (cat. no. IPK5027, Biocare Medical; blue precipitate). Once staining was completed, the slides were removed from the automated stainer and immersed in TBST for 3 min, then dehydrated in graded ethanol (70%, 96%, 100%, 2×) for 3 min and xylene (or xylene substitute in the case of duplex IHC), three times each for 3 min. Finally, coverslipping was carried out with a Tissue-Tek automated cover slipper (Sakura Finetek) using the Ecomount medium (cat. no. EM897L, Biocare Medical).

### Creation of the CPS

Our quantitative neuropathological data, ${X}_{d}^{\,m,l}$, was measured as *d* = 1… *D* = number of donors, in *l* = 1… *L* cortical layers and *m* = 1… *M* distinct neuropathological measurements. To estimate a CPS of pathological severity in MTG for each brain donor, we created a latent Bayesian statistical model. We assigned to each donor a latent variable, termed ${t}_{d}\in [\mathrm{0,1}]$, representing CPS. In addition, we proposed to infer the most probable donor permutation $\pi$ to facilitate latent space exploration. As described in the main text, the observation model has a mean value dictated by the exponential biophysical dynamics $\mu ={e}^{{{k}_{m}^{l}{t}}_{d}+{a}_{m}^{l}}$, where ${k}_{m}^{l}$ and ${a}_{m}^{l}$ are the per-layer and per-quantifiable neuropathological measurement dynamic parameters representing rise time and initial condition, respectively. We assumed that our data were corrupted with observational noise described with a Poisson distribution. We imposed Bayesian priors on this model and obtained the following hierarchical Bayesian generative statistical model:

$$\begin{array}{l}\pi \sim{\mathrm{Uniform}}(\pi )\\ t \sim {\mathrm{Uniform}}({\mathrm{Partition}}\,{\mathrm{Simplex}})\\ {a}_{m},{k}_{m} \sim {\mathrm{Normal}}(0,1)\\ {a}_{m}^{l} \sim {\mathrm{Normal}}({a}_{m},1)\\ {k}_{m}^{l} \sim {\mathrm{Normal}}({k}_{m},1)\\ {X}_{d}^{m,l} \sim {\mathrm{Poisson}}({e}^{{k}_{m}^{l}{t}_{\pi (d)}+{a}_{m}^{l}}\,)\end{array}$$

in which the symbol ~ indicates that we are taking draws from a distribution. The hierarchical nature of this model enables ‘borrowing information’ across layers; for each measurement, the layer-specific parameters ${k}_{m}^{l}$ and ${a}_{m}^{l}$ are sampled from their population parameters *k*_*m*_ and *a*_*m*_.

We performed approximate Bayesian inference in this model to obtain draws from an approximate posterior distribution given the model and the underlying priors for *a*, *k*, *π* and *t*. Our inferential strategy is based on a Gibbs block coordinate sampler where we iteratively sampled from each block of variables (*t*, *π* or (*a*, *k*)) conditioned on the others being fixed. To sample an element *t* of the simplex that we unequivocally associated with an increasing sequence of times fixed, we used the sampler described in ref. ^99^. To sample permutations of *π*, we resorted to the parametric Gumbel-Sinkhorn family of distributions over permutations^100^ to approximate the otherwise intractable conditional distribution (hence, our method was approximate). Finally, to sample the model parameters (*a*, *k*) we used Stan (v.2.34) with 1,000 burn-out iterations and collected samples from multiple chains. After the initial burned-out samples, we iterated through this procedure.

### Tissue processing for single-nucleus isolations

Cortical areas of interest were identified on tissue slab photographs taken at the time of autopsy and at the time of dissection using the Allen Human Reference Atlas as a guide for region localization. MTG was sampled at the level of first appearance of the lateral geniculate nucleus corresponding to the intermediate subdivision of area (A) 21. A9 was sampled in tissue slabs anterior to the first appearance of the corpus callosum within the superior frontal gyrus (SFG) corresponding to the rostrodorsal portion of the PFC (A9 (ref. ^101^)). Tissue blocks encompassed the full height of the cortex from the pia to the white matter (~5 mm) and were ~2–3 mm wide and 4 mm thick. To dissect regions of interest, tissue slabs were removed from storage at −80 °C, briefly transferred to a −20 °C freezer to prevent tissue shattering during dissection and then handled on a custom cold table maintained −20 °C during dissection. Dissections were performed using dry-ice-cooled razor blades or scalpels to prevent warming of tissues. Photographs were taken before and after each dissection to document the precise location of each resected tissue block. Dissected tissue samples were then transferred to vacuum seal bags, sealed and stored at −80 °C until the time of use. Single-nucleus suspensions were generated using a previously described standard procedure (https://www.protocols.io/view/isolation-of-nuclei-from-adult-human-brain-tissue-ewov149p7vr2/v2). Briefly, after tissue homogenization, isolated nuclei were stained with a primary antibody against NeuN (FCMAB317PE, Sigma-Aldrich) to label neuronal nuclei. Nucleus samples were analyzed using a BD FACSAria flow cytometer (software BD Diva v.8.0, BD Biosciences); nuclei were sorted using a standard gating strategy to exclude multiplets^24^ (Supplementary Table 1). A defined mixture of neuronal (70% from the NeuN^+^ gate) and nonneuronal (30% from the NeuN^−^ gate) nuclei was sorted for each sample. Nuclei isolated for 10X Genomics v.3.1 snRNA-seq were concentrated by centrifugation after FANS, and were frozen and stored at −80 °C until later chip loading. Nuclei isolated for 10X Genomics Multiome and 10X Genomics Single Cell ATAC v.1.1 were concentrated by centrifugation after FANS and were immediately processed for chip loading.

### Isolation of RNA and determination of RIN from frozen human brain tissue

To assess RNA quality, three tissue samples (roughly 50 mg each) were collected from the tissue slab corresponding to the frontal pole of each donor brain. Tissue samples were collected from three different regions of the tissue slab to assess within-slab variability in RNA quality. Dissected tissues were stored in 1.5-ml microcentrifuge tubes on dry ice or in −80 °C until the time of RNA isolation. Tissue samples were homogenized using a sterile Takara BioMasher (cat. no. 9791A). RNA isolation was performed using a QIAGEN RNeasy Plus Mini Kit (cat. no. 74134) or a Takara NucleoSpin RNA Plus kit (cat. no. 740984) according to the manufacturer’s protocol. RIN values for each sample were determined using the Agilent RNA 6000 Nano chip kit (cat. no. 5067-1511) and an Agilent Bioanalyzer 2100 instrument according to the manufacturer’s protocol.

### 10X genomics sample processing

10X Genomics chip loading and postprocessing of the emulsions to the sequencing libraries were done with the Chromium Next GEM Single Cell 3′ Gene Expression v.3.1, Chromium Next GEM Single Cell ATAC v.1.1 and Chromium Next GEM Single Cell Multiome ATAC Gene Expression kits according to the manufacturer’s guidelines. Nuclei concentration was calculated either manually using a disposable hemocytometer (DHC-NO1, INCYTO) or using the NC3000 NucleoCounter.

### 10X sequencing and preprocessing

All 10X libraries were sequenced according to the manufacturer’s specifications on a NovaSeq 6000 using either a NovaSeq X or S4 flow cell. Reads were demultiplexed to FASTQ files using BCL Convert (v.4.2.7) for libraries run on NovaSeq X flow cells and bcl2fastq (v.2-20-0) for libraries run on S4 flow cells. Reads from snRNA-seq libraries were mapped to the 10X Genomics official human reference (GRCh38-2020-A); UMIs per gene were counted using the Cell Ranger (v.6.1.1) pipeline with the --include--introns parameter included. Reads from the snATAC–seq and snMultiome libraries were mapped to the same reference using Cellr Anger ATAC (v.2.0.0) and Cell Ranger Arc (v.2.0.0) pipelines, respectively, with default parameters.

### Comparing the peak universes of severely affected donors to other high pathology donors

We used the ChromA^102^ Python package (https://github.com/marianogabitto/ChromA, v.2.1.2) with default parameters on fragment files from each donor individually to call a set of donor-specific peaks. As part of this procedure, peaks are filtered by whitelisted regions existing in 10X Cell Ranger ATAC.

All peak sets were then combined using concatenation. They were then subjected to fusion, that is, if two peaks shared a 10% overlap, their coordinates would be merged using the default BEDTools^103^ merge mode. ChromA was used to then compute counts by peaks matrices for each donor using the peak set defined above using fragment and peak files as inputs.

### Spatial transcriptomics data collection

Human postmortem frozen brain tissue was embedded in optimal cutting temperature medium (cat. no. 25608-930, VWR) and sectioned on a Leica cryostat at −17 °C at 10 μm onto Vizgen MERSCOPE coverslips. These sections were then processed for MERSCOPE imaging according to the manufacturer’s instructions. Briefly, sections were allowed to adhere to these coverslips at room temperature for 10 min before a 1-min wash in nuclease-free PBS and fixation for 15 min in 4% paraformaldehyde in PBS. Fixation was followed by three 5-min washes in PBS before a 1-min wash in 70% ethanol. Fixed sections were then stored in 70% ethanol at 4 °C before use and for up to 1 month. Human sections were photobleached using a 240 W LED array for 72 h at 4 °C (with temperature monitoring to keep samples below 17 °C) before hybridization, and then washed in 5 ml Sample Prep Wash Buffer (cat. no. 20300001, Vizgen) in a 5-cm Petri dish. Sections were then incubated in 5 ml Formamide Wash Buffer (cat. no. 20300002, Vizgen) at 37 °C for 30 min. Sections were hybridized by placing 50 μl of Vizgen Gene Panel Mix onto the section, covering with parafilm and incubating at 37 °C for 36–48 h in a humidified hybridization oven. After hybridization, sections were washed twice in 5 ml Formamide Wash Buffer for 30 min at 47 °C. Sections were then embedded in acrylamide by polymerizing Vizgen Embedding Premix (cat. no. 20300004) according to the manufacturer’s instructions. Sections were embedded by inverting sections onto 110 μl of Embedding Premix and 10% ammonium persulfate (cat. no. A3678, Sigma-Aldrich) and TEMED (cat. no. 161-0800, Bio-Rad Laboratories) solution applied to a Gel Slick (cat. no. 50640, Lonza) treated 2×3 inch glass slide. The coverslips were pressed gently onto the acrylamide solution and allowed to polymerize for 1.5 h. After embedding, sections were cleared for 24–48 h with a mixture of Vizgen Clearing Solution (cat. no. 20300003) and proteinase K (cat. no. P8107S, New England Biolabs) according to the manufacturer’s instructions. After clearing, sections were washed two times for 5 min in Sample Prep Wash Buffer (cat. no. 20300001). Vizgen 4′,6-diamidino-2-phenylindole (DAPI) and PolyT Stain (cat. no. 20300021) was applied to each section for 15 min followed by a 10-min wash in Formamide Wash Buffer. Formamide Wash Buffer was removed and replaced with Sample Prep Wash Buffer during the MERSCOPE setup. then, 100 μl of RNase Inhibitor (cat. no. M0314L, New England BioLabs) was added to 250 μl Imaging Buffer Activator (cat. no. 203000015); this mixture was added via the cartridge activation port to a pre-thawed and mixed MERSCOPE Imaging cartridge (cat. no. 1040004, Vizgen). Then, 15 ml mineral oil (cat. no. m5904-6X500ML, Sigma-Aldrich) was added to the activation port and the MERSCOPE fluidics system was primed according to the instructions provided by Vizgen. The flow chamber was assembled with the hybridized and cleared section coverslip according to the specifications provided by Vizgen; the imaging session was initiated after collection of a 10X mosaic DAPI image and selection of the imaging area. Specimens were imaged and automatically decoded into transcript location data and a cell-by-gene table. All postprocessing and segmentation was completed using the vizgen-postprocessing docker container v.0.0.5 (https://github.com/Vizgen/vizgen-postprocessing). For each section, segmentation was run on a single z-plane (z = z3). Segmentation was a combination of cellpose-cyto2 2D segmentation (with contrast-limited adaptive histogram equalization and normalized DAPI and PolyT images as inputs) and cellpose nuclei-only segmentation (using contrast-limited adaptive histogram equalization and normalized DAPI images only). Results were then fused using the harmonize strategy and returned as cell metadata summary files and parquet mosaic geometry files. If segmentation failed on the *z* = z3 image plane, *z* = z4 image data were used instead.

### Compositional analysis of supertypes

To model changes in the composition of cell types as a function of CPS and other covariates, we used the Bayesian method scCODA^104^ (v.0.1.7). We tested compositional changes in neuronal and nonneuronal nuclei separately because they were sorted to have a defined ratio (70% neuronal nuclei, 30% nonneuronal nuclei in each donor). To do this, we created separate AnnData objects of neuronal and nonneuronal nuclei with supertype annotations, sequencing library IDs and relevant donor-level covariate information (noted below) for all snRNA-seq and snMultiome nuclei formatted as per https://sccoda.readthedocs.io/en/latest/data.html using the sccoda.util.cell_composition_data function with cell_type_identifier set to supertype and sample_identifier set to the sequencing library ID. As we did not know which supertypes would be affected by AD, we ran models with each supertype set as the unchanged reference population, as recommended by the authors of scCODA. We set up an ensemble of models to test whether supertypes were credibly affected across cognitive status (no dementia (0) versus dementia (1)), ADNC (no AD (0), low (1/3), intermediate (2/3), high (1)) and CPS (interval (0,1)) using the scconda.util.comp_ana.CompositionalAnalysis function with formula set to sex + age at death + race + 10X chemistry + *APOE4* status + (disease covariate) with each supertype as the reference population (yielding a total of 417 models) and obtained posterior estimates for each parameter with a Markov chain Monte Carlo process implemented in the sample_hmc function with default parameters. The sampling occasionally stayed at fixed points, so we reran models with fewer than 60% accepted epochs. We defined credibly affected supertypes as those that had a mean inclusion probability across models greater than 0.8. The same approach was used for testing compositional changes across CPS in the snRNA-seq data from the SEA-AD A9 dataset using the formula sex + age at death + ace + (disease covariate) and across ADNC in snRNA-seq data from refs. ^13^^,^^14^ using the formula sex + age at death + *APOE4* status + (disease covariate), with the sample_identifier set to donor ID as there was no one-to-one or one-to-many relationship between donors and libraries across these datasets.

### Identifying differential electrophysiological features in vulnerable neurons from patch-seq data

We obtained publicly available patch-seq^20–22^ data from 2,602 cells, originating from slices from 401 donors. These cells were recorded in healthy tissue extracted during surgical resection due to cancer pathology or epilepsy (95% of cases) and hydrocephalus, encephalomyelitis, aneurism and ventriculoperitoneal shunt (5% of cases). We subsetted the dataset to include only cells obtained from the MTG and mapped the snRNA-seq data from them to the SEA-AD MTG cellular taxonomy using the same iterative scVI and scANVI approach described above for the SEA-AD nuclei and the publicly available datasets.

We tested for differences in each electrophysiological feature between vulnerable and unaffected neurons in Sst and Pvalb using a logistic regression implemented in the Python scikit-learn package (v.1.1.1) using the sklearn.linear_model.LogisticRegression function with default parameters. The outcome variable was 0 for unaffected supertypes and 1 for vulnerable ones; all models were adjusted for age at death, sex and whether the slices were cultured or not cultured. Covariates were adjusted with either the minmax_scale (for age at death) or the OneHotEncoder (for sex and culture status) functions in sklearn.preprocessing. We then fitted the model using the sklearn.linear_model.LogisticRegression.fit function. *P* values were corrected for multiple hypothesis testing with the Benjamini–Hochberg method using an alpha value of 0.05.

### Statistics, data visualization and reproducibility

All data were analyzed either in Python with custom-written scripts or libraries that are described extensively within this section of the Methods or the Supplementary Note. Data distribution was assumed to be Poisson for quantitative neuropathological data, zero-inflated negative binomial for gene expression data, Bernoulli for chromatin accessibility data and negative binomial for spatial transcriptomic data, but this was not formally tested. Data collection and analysis were not performed blind to the conditions of the experiments. Two data points were excluded from the analysis of single-nucleus datasets because of low-quality RIN values; 11 data points were excluded from the gene expression tests given that these donors belonged to the severely affected group and exhibited systematically lower-quality data. No randomization was necessary due to the experimental conditions. No statistical methods were used to predetermine sample sizes, but our sample sizes are similar to those reported in previous publications^5–14^. All experiments showing representative images were repeated with similar outcomes. For all box plots, the center is the median, the minima and maxima of the box are defined by the IQR and the whiskers are 1.5 times the IQR; unless otherwise stated in the figure legend, all data points are shown.

### Reporting summary

Further information on research design is available in the Nature Portfolio Reporting Summary linked to this article.

## Online content

Any methods, additional references, Nature Portfolio reporting summaries, source data, extended data, supplementary information, acknowledgements, peer review information; details of author contributions and competing interests; and statements of data and code availability are available at 10.1038/s41593-024-01774-5.

## Supplementary information

Supplementary Information Supplementary Note and Supplementary Fig. 1.

Reporting Summary

Supplementary Table Supplementary Tables 1–8.

## Data Availability

FASTQ files containing the sequencing data from the snRNA-seq, snATAC–seq and snMultiome assays are available through controlled access at Sage Bionetworks (accession no. syn26223298). Instructions for access to data on the AD Knowledge Portal is provided by Sage Bionetworks. Nuclei-by-gene matrices with counts and normalized expression values from the snRNA-seq and snMultiome assays are available through the Open Data Registry in an AWS bucket (sea-ad-single-cell-profiling) as AnnData objects (h5ad files) and viewable on CELLxGENE and Allen Brain Cell Atlas (https://portal.brain-map.org/atlases-and-data/bkp/abc-atlas). Nuclei-by-peak matrices for the snATAC–seq data (with peaks called across all nuclei) are in the same AWS bucket. Cell-by-gene matrices containing spatial coordinates from MERFISH data are also available through the Open Data Registry in an AWS bucket (sea-ad-spatial-transcriptomics). The MERFISH data are also viewable on the Allen Brain Cell Atlas. Donor, library and cell-level metadata are available in these objects and also at https://portal.brain-map.org/explore/seattle-alzheimers-disease. Raw images from the quantitative neuropathology data are available at the Open Data Registry on AWS in an AWS bucket (sea-ad-quantitative-neuropathology) and the variables derived from HALO at https://portal.brain-map.org/explore/seattle-alzheimers-disease. We obtained raw sequencing reads from ten publicly available datasets that performed single-cell or single-nucleus RNA-seq on or near the PFC of human cohorts that included sporadic AD donors. These included datasets from the AD Knowledge Portal hosted on Synapse: ref. ^5^ (accession no. syn18485175; stated brain region: PFC, Brodmann area 10), ref. ^8^ (accession no. syn21670836; stated brain region: DLPFC, ref. ^7^ (accession no. syn21438358; stated brain region: DLPFC), ref. ^12^ (accession no. syn16780177; stated brain region: DLPFC), ref. ^13^ (accession no. syn31512863; stated brain region: DLPFC, Brodmann area 9) and ref. ^14^ (accession no. syn52293417; stated brain region: DLPFC). It also included datasets from the Sequencing Read Archive (SRA): ref. ^6^ (accession no. PRJNA662923; stated brain region: PFC), ref. ^9^ (accession no. PRJNA615180; stated brain region: SFG), ref. ^10^ (accession no. PRJNA729525; stated brain region: PFC) and ref. ^11^ (accession no. PRJNA686798; stated brain region: SFC). From each of these repositories, including separate data use agreements with the Rush ADRC (for donors from the ROSMAP cohort), we also obtained clinical metadata and harmonized it to a standardized schema included. We are working with the relevant data repositories to obtain approval to share the reprocessed and integrated, publicly available datasets under the data use agreements that govern them. In the meantime, we have placed the cell type annotations in an AWS bucket (sea-ad-single-cell-profiling) without gene expression data or donor metadata.

## Code availability

The collection of scripts used to annotate the SEA-AD and publicly available datasets, perform all the analyses and create each figure are available at the Allen Institute GitHub page: https://github.com/AllenInstitute/SEA-AD_2024.
