# Supplementary material for: Integrated multimodal cell atlas of Alzheimer’s disease
Source: Nat Neurosci. 2024 Oct 14;27(12):2366–83. doi: 10.1038/s41593-024-01774-5 (PMC11614693; doi:10.1038/s41593-024-01774-5)

nature neuroscience

Supplementary information

https://doi.org/10.1038/s41593-024-01774-5

# Integrated multimodal cell atlas of Alzheimer's disease

In the format provided by the authors and unedited

# Supplementary Information - Integrated multimodal cell atlas of Alzheimer's disease

## SEA-AD cohort description and brain tissue collection

The ACT study is a community cohort study of older adults from Kaiser Permanente Washington (KPW), formerly Group Health, in partnership with the UW. The ACT study seeks to understand the various conditions and life-long medical history that can contribute to neurodegeneration and dementia and has been continuously running since 1994, making it the longest running study of its kind. In 2004, ACT began continuous enrollment with the same methods to replace attrition from dementia, dropout, and death, ensuring a consistent cohort of ≥2,000 at risk for dementia. Total enrollment is nearing 6,000, with over 1,000 incident dementia cases; more than 900 have had autopsies to date with an average rate of approximately 45-55 per year. The study completeness of the follow up index is between 95 to 97%. Subjects aged 65 or older without dementia are invited to enroll by random selection from the greater Seattle area patient population of KPW Seattle and undergo bi-annual study visits for physical and mental examinations. In addition to this study data, the full medical record is available for research through KPW. Approximately 55% of ACT autopsies are from people with no dementia at their last evaluation, while roughly 45% meet criteria for dementia. Thus, the ACT study provides an outstanding cohort of well-characterized subjects with a range of mixed pathologies including many controls appropriate for this study. Approximately 30% of the ACT cohort consents to research brain donation upon death, and tissue collection is coordinated by the UW BRaIN lab, which preserves brain tissue for fixed, frozen, and fresh preparations (described below), as well as performing a full post-mortem neuropathological examination and diagnosis by Board-certified neuropathologists using the NIA-AA and other relevant, current guidelines.

The UW Alzheimer's Disease Research Center (ADRC) has been continuously funded by NIH since 1984. It is part of a nationwide network of ADRCs funded through the NIA and contributes uniquely to this premier program through its vision of precision medicine for AD: comprehensive investigation of an individual's risk, surveillance with accurate and early detection of pathophysiologic processes while still preclinical, and interventions tailored to an individual's molecular drivers of disease. Participants enrolled in the UW ADRC Clinical Core undergo annual study visits, including mental and physical exams, donations of biospecimens including blood and serum, and family interviews. The UW ADRC is advancing understanding of clinical and mechanistic heterogeneity of Alzheimer's disease, developing pre-clinical biomarkers, and, in close collaboration with the ACT study, contributing to the state of the art in neuropathological description of the disease. For participants who consent to brain donation, tissue is also collected by the UW BRaIN lab, and is preserved and treated with the same full post-mortem diagnosis and neuropathological work up as described above.

Human brain tissue was collected at rapid autopsy (postmortem interval <12 hours, mean close to 7 hours, **Extended Data Fig. 1a**). One hemisphere (randomly selected) was embedded in alginate for

uniform coronal slicing (4mm), with alternating slabs fixed in 10% neutral buffered formalin or frozen in a dry ice isopentane slurry on Teflon-coated plates[1]. Superior and Middle Temporal Gyrus (STG-MTG) for quantitative neuropathology was sampled from fixed slabs and subjected to standard processing, embedding in paraffin (**Extended Data Fig. 1b**).

## Methods for Quantitative Neuropathological Analysis

### Image acquisition of whole slide images

To analyze the different slides obtained from the MTG tissue samples processed for IHC, the slides were scanned on the Aperio AT2 digital scanner (Leica, software version 102.0.7.5), which captures sequential images of a 20x field of view, using slide settings optimized for our IHC protocols which are subsequently assembled or stitched into whole slide images (WSIs) to be exact replicas of the glass slides. All images are scanned at 20x magnification and using the same gain, brightness and exposure times to avoid image to image variations (**Extended Data Fig. 2a**)

### Quantification of whole slide images

The quantitative pathological assessment for the WSIs obtained from the MTG region were analyzed using the HALO® v.3.4.2986 (Indica labs, Albuquerque, New Mexico, USA).

First, DenseNet[2], a deep learning convolutional neural network was trained to segment MTG cortical ribbon into cortical layers. The DenseNet network is a minimally pretrained classifier developed to recognize patterns in the tissue structure provided by Halo. Training data was created by manually annotating cortical layers labelled with NeuN in 10 cases. Based on the cellular architecture and the relative position withing the cortical ribbon the following layers were annotated: Layer1 (molecular layer), layer 2 (external granular layer), layer 3 (external pyramidal layer), layer 4 (Internal granular layer) and layers 5-6 (internal pyramidal and multiform layers) (Figure 1). Then the trained classifier was applied to the NeuN-labelled sides from all donors. All results of the automatic segmentation were examined by a scientist trained in cortical neuroanatomy and adjusted when necessary. Manual adjustment of the annotations also included removal of staining artifacts and non-parenchymal structures, such as large blood vessels by drawing exclusion areas around them.

Second, using the Serial Section registration tool, all 5 WSIs belonging to the same case (labelled with NeuN, GFAP, α-Syn, Aβ combined with Iba1, and pTau combined with pTDP-43) were registered to each other in order to establish anatomical correspondence between all 5 tissue sections, and the cortical annotations from the NeuN-labelled slide were copied to the other 4 IHC stained slides (noted above). We then applied different algorithms and approaches to obtain stain-specific metrics from all the slides for each cortical layer. Area quantification algorithm (Area Quantification module) was used for determining the area of positive staining for all proteins of interest (NeuN, GFAP, Iba1, Aβ, pTau, α-Syn, and pTDP-43). Multiplex IHC module for used to determine the number of cells displaying positive labelling for NeuN, pTau, α-Syn, and pTDP-43). For the double labelled slides, Multiplex IHC module was used to estimate the area of co-localization of pTau with pTDP-43, and Aβ with Iba1. Microglia Activation module was used to determine the number of cells positive for Iba1, measure the cell process area and length, as well as to classify the cells according to the activation state (activated vs not, based on the process thickness). The

same module was adapted to estimate the process length and process area for cells positive for GFAP. In the slides double-labelled for Aβ and Iba1 the Object Colocalization module was used to determine the number of abet Aβ-positive objects (amyloid plaques), the average object area, median object diameter, and the number of objects that were double-positive for Aβ and Iba1.

Development, optimization, and testing of all analysis algorithms was done by a scientist trained in neuropathology. The final quantitative neuropathology dataset includes raw measurements (absolute values) and metrics normalized to the unit area. (**Supplementary Table 2**).

## Methods for QC and Analysis of Severely Affected Donors

### Identification of donors with low quality tissue

To identify donors with correlated poor tissue-level and pre-sequencing metrics (brain pH, brain weight, postmortem interval, RIN, cDNA amplification concentration, and snRNA-seq library insert size) we constructed an AnnData object using the scanpy[3] python package (version 1.9.1). Each donor was treated as an observation and each quality control metric above as a variable instead of the typical cell by gene construction. We then centered and scaled each metric with the scanpy.pp.scale function with default parameters and performed principal component analysis on the matrix with the scanpy.pp.pca function also with default parameters. Two donors were outliers on the first principal component (e.g. had values beyond 1.5 times the interquartile range centered at the median), which were driven by severely low RIN scores and brain pH. These donors were excluded from downstream analyses.

### Identification of severely affected donors

To identify donors with systematically lower data quality by *post-sequencing metrics,* we repeated the above procedure with library-level snRNA-seq and snATAC-seq metrics from the cellranger and cellranger-atac pipelines. The metrics included mean raw reads per cell, median UMIs per cell, median genes detected per cell, fraction of reads mapped to the genome, fraction of reads mapped uniquely to the genome, fraction of reads mapped to intronic regions, fraction of reads mapped to exonic regions, fraction of reads mapped to intergenic regions, fraction of reads mapped antisense, fraction of reads mapped to the transcriptome, fraction of transcriptomic reads in cells, and total genes detected across the library for snRNA-seq and mean raw reads per cell, median fragments detected per cell, fraction of uniquely mapped reads, fraction of the genome in peaks, fraction of fragments overlapping peaks, fraction of fragments overlapping transcription start site (TSS), TSS enrichment, and fraction of transposition events in peaks in cells for snATAC-seq. The first principle component for the snRNA-seq and snATAC-seq metrics explained 71.4% and 65.5% of the variance, respectively, so were taken as composite scores of these highly correlated library level metrics. 11 donors were outliers along the first principle component from both modalities (again defined as values beyond 1.5 times the interquartile range centered at the median), so were flagged as having systematically lower data quality. We classified these donors as severely affected based on differences in the steepness of their memory decline in life compared to other donors with similar pathology (see methods section Testing for differential cognitive slopes in severely affected donors below and Results section SEA-AD: Multimodal profiling Alzheimer's disease progression across wide pathological stages above). There were no severely affected donors in the snMultiome dataset.

### Testing for differential cognitive slopes in severely affected donors

Cognitive testing was previously co-calibrated and harmonized into cognitive composites for memory, executive functioning, visuospatial recognition, and language for all ACT and UW ADRC study participants using confirmatory factor analyses [4]. To test for differences in the decline of these domains between severely affected and other high pathology donors, each of the composite scores was standardized to a normal distribution (i.e. *N(0, 1)*) across the broader cohort they came from at baseline. We calculated slopes of memory decline over time using a mixed-effects models using the mixed function from Stata (version 18), where time was parameterized as years before death. We also created a multinomial outcome variable with a reference level consisting of lower pathology donors (i.e. donors with ADNC score Not AD, Low, or Intermediate) and two distinct test groups. The first included donors who were ADNC high, but were not outliers based on library level metrics and the second included donors who were outliers. We then ran a multinomial logistic regression for our outcome variable using the mlogit function from Stata on the slope of each cognitive domain separately, adjusting for age at death and sex and study site (i.e. ACT or UW ADRC). P-values from the models were Bonferroni corrected with the number of cognitive domains tested and called as significant with an alpha value of 0.05.

### Testing for differential quantitative neuropathological features in severely affected donors

We tested for differences in each quantitative neuropathology variable (described above in Quantification of whole slide images) using a similar model implemented in the python scikit-learn package (version 1.1.1) using the sklearn.linear_model.LogisticRegression function with default parameters. The multinomial outcome variable was identical to that above and all models adjusted for age at death and sex. Covariates were adjusted with either the minmax_scale (for age at death) or the OneHotEncoder (for sex) functions in sklearn.preprocessing. We then fit the model using the sklearn.linear_model.LogisticRegression.fit function. P-values were corrected for multiple hypothesis testing with the Benjamini-Hochberg method using an alpha value of 0.05.

### Transcription factor motif enrichment in peaks specific to severely affected donors

To explore the relationship between Transcription factors (TF) and regions of accessible chromatin specific to severely affected donors, we used the human TF motif list from HOCOMOCO[5] as an input to FIMO[6]. We collected the list of peaks unique to severely affected donors (cf. Testing for differential peaks in severely affected donors) and retrieved fasta sequences using BED2FASTA. Finally, we run fimo with the following paramters: fimo --oc seq.fa --verbosity 1 --bgfile --nrdb-- --thresh 1.0E-4 H12CORE_meme_format.meme seq. The results were then filtered on q-value <=.05 and sum aggregated to identify the TF with top represented motifs.

## Methods for Omics Processing.

### Creation of supertypes in neurotypical reference data

We defined supertypes as a set of fine-grained cell type annotations for single nucleus expression data that could be reliably predicted on held-out neurotypical reference data (where ground truth labels were assigned as described above) using state-of-the-art machine learning approaches[7,8] in the scvi-tools

python package (version 0.14.3). From 5 neurotypical donors in a related study with roughly 140K nuclei captured with 10x snRNA-seq[9] we systematically held out labels from 1 donor and used scVI to compute joint latent space then scANVI to iteratively and probabilistically predict their class (3 labels), subclass (24 labels), and then cluster (151 labels). When predicting each nucleus' class, we selected the top 2,000 highly variable genes (using the scanpy.pp.highly_variable_genes function implemented in the scanpy[3] python package, version 1.9.1, with the flavor parameter set to seurat_v3, n_top_genes parameter set to 2000) along with the top 500 differentially expressed genes unique to each class (calculated from the reference cells from donors that had their labels retained using a Wilcoxon rank sum test implemented in scanpy.tl.rank_gene_groups with the method parameter set to wilcoxon, tie_correct parameter set to True, pts parameter set to True) to use as features in training the model and specified the donor's ID and number of genes detected as categorical and continuous covariates, respectively, in the setup_anndata function implemented in scvi.model.SCVI and scvi.model.SCANVI. 2 hidden layers were used for all models, specified by setting n_layer to 2 when initializing the model. The scVI model was then trained using the scvi.model.SCVI.train function with max_epochs set to 200 and passed to scANVI with the scvi.model.SCANVI.from_scvi_model function. The scANVI model was then trained for an additional 20 epochs using the scvi.model.SCANVI.train function. We then obtained the latent representation from the scANVI model with the scvi.model.SCANVI.get_latent_representation function and label predictions with the scvi.model.SCANVI.predict function where the soft parameter was set to True to export probabilities. Nuclei were then separated by their predicted class and features were re-selected with the same criteria to predict subclasses and again in predicting clusters. A differential expression test (same Wilcoxon test and parameters as above) was run on clusters with an F1 score below 0.7, and those without 3 positive markers when compared against nuclei from their constituent subclass (cutoff paramters: corrected p-value <0.05, fraction of in-group expression >0.7, fraction out of group expression <0.3) were dropped from the taxonomy, with the remaining clusters representing supertypes.

**Mapping transcriptomic SEA-AD nuclei to reference supertypes**

SEA-AD nuclei with transcriptomic data (from either snRNA-seq or snMultiome) with fewer than 500 genes detected were removed upstream of supertype mapping. After defining supertypes in neurotypical donors, we iteratively and probabilistically predicted each SEA-AD nucleus's class, subclass, and supertype using scANVI[8], as above. Briefly, each SEA-AD nucleus' class was predicted after projecting them into a shared latent space with reference nuclei using models trained with 2000 highly variable genes and 500 differentially expressed genes per class (from reference data, where donor name and number of genes were passed as categorical and continuous covariates, respectively). Nuclei were then split by predicted class, projected into a new class-specific latent space where subclass was predicted, and again for supertype. The subclass-specific latent spaces were then used to construct a nearest neighbor graph with the scanpy.pp.neighbors function with default settings and represented with a two-dimensional uniform manifold approximation and projection (UMAP) computed with scanpy.tl.umap with default settings. Predictions from scANVI were evaluated by probabilities from the model and by known marker gene expression (signature scores were computed by summing the absolute value of the t-statistic between reference and SEA-AD nuclei for the top 50 differentially expressed genes for each supertype computed from reference nuclei using the same Wilcoxon test as above). Areas of the nearest neighbor graph with few reference nuclei could represent droplets with ambient RNA, multiplet nuclei, dying cells, or

transcriptional states missing from the reference, unique to a donor, or found only in aging or disease. To assess these possibilities, we fractured the graph into tens to hundreds of clusters (called metacells) using high resolution Leiden clustering implemented in the scanpy.tl.leiden function with the resolution parameter set to 5 and then merged them based on differential gene expression using the merge_clusters function in the transcriptomics_clustering python package from the Allen Institute (https://github.com/AllenInstitute/transcriptomic_clustering [will be made public upon publication of this manuscript]) with default merging thresholds for gene expression and cluster size. Clusters and metacells were then flagged and removed if they had poor group doublet scores[1], fraction of mitochondrial reads, number of genes detected, or donor entropy (computed with scipy.stats.entropy with default parameters, version 1.8.1), with cutoffs adjusted for each subclass based on their distributions (to account for dramatically different RNA content found across cell types).

The NIH Brain initiative snRNA-seq dataset generated from A9 of neurotypical reference donors[10] was mapped to the MTG cellular taxonomy using the same iterative procedure. A9 snRNA-seq data from SEA-AD donors were then mapped to the A9 neurotypical reference dataset with the predicted MTG labels to ensure a common cellular taxonomy using the same procedure. All downstream quality control steps were also performed identically to those done for the SEA-AD dataset.

**Expanding the reference taxonomy for non-neuronal cells**

After removing common technical axes of variation, we next identified nuclei that were transcriptionally distinct from the reference and added them to our supertype taxonomy. To do so, we constructed a new latent space for each subclass using scVI, where the model was passed the supertype predictions as cell labels; gene dispersion was allowed to vary per supertype; sex, race and 10x technology (multiome versus singlome) were included as categorical covariates; and the number of genes detected in each nucleus and the donor age at death were passed as continuous covariates. We then d trained the scVI model and obtained the latent representation using the same functions and parameters described above. Using the neighborhood graph computed from this latent representation, we clustered the nuclei into tens to hundreds of groups and merged them based on differential gene expression using the transcriptomics_clustering package, as above. We defined merged clusters with fewer than 10% of all reference cells or of any single supertype as having poor reference support and added them to the taxonomy (systematically named Subclass_Number-SEAAD). In cases where more than 90% of SEA-AD nuclei within these poorly supported groups were predicted to be one supertype, their new label reflected that assignment (e.g., Subclass_SupertypeNumber_Number-SEAAD). These cell type assignments are used as baseline for the analyses, plots, and tools developed for the web product and this manuscript.

**Mapping epigenomic SEA-AD nuclei to supertypes**

To define the peak universe used across all nuclei with epigenomic data (from snATAC-seq and snMulitome), we first separated the 84 donors by their AD neuropathological changes into 4 groups (Not AD, Low, Intermediate, and High) and randomly selected 5 donors from each group (excluding severely affected donors). In each group, we identified group-specific peaks using the atac function in the ChromA[11] python package (https://github.com/marianogabitto/ChromA, version 2.1.2) with default parameters. We created a union peak set across the 4 groups using the version 2.3.11 bedtoolsmerge function. We

then used the count function in ChromA with default parameters to quantify the number of UMIs within each peak to construct a nucleus by peak matrix. We next integrated the snRNA-seq, snATAC-seq, and snMultiome datasets using MultiVI[12] (from scvi-tools 0.14.3), with modality (e.g. snRNA-seq, snATAC-seq, or snMulitome) set as the batch_key, and donor ID and sex passed to the model as categorical covariates in the scvi.model.MULTIVI.setup_anndata function. After training the model using the scvi.model.MULTIVI.train function with default parameters, we obtained the joint latent representation with the scvi.model.MULTIVI.get_latent_representation function and constructed the nearest neighbor graph across modalities as above and clustered the nuclei using the leiden algorithm implemented in scanpy.tl.leiden with default settings (not high-resolution clustering). We performed quality control on snATAC-seq nuclei based on those from snRNA-seq and snMulitome data in this integrated space. Briefly, we calculated a quality control score for each snATAC-seq nucleus by computing the fraction of its neighbors that were flagged as low-quality snRNA-seq and snMultiome nuclei. snATAC-seq nuclei in leiden clusters with scores greater than 0.5 were removed. We then transferred the subclass labels to snATAC-seq nuclei by labeling them based on what the majority snRNA-seq and snMultiome nearest neighbors. We separated the epigenomic nuclei based on each subclass and called peaks (as above) within subclasses from 5 randomly selected non-SA donors using ChromA to optimize the feature space. Finally, we integrated the multiple modalities data and transferred supertype labels with each subclass using MultiVI, as above.

**Common reprocessing, integration and mapping of publicly available datasets**

We obtained raw sequencing reads from 10 publicly available datasets[13–22] that performed single cell or single nucleus RNA-seq on or near the PFC of human cohorts that included sporadic AD donors. These included datasets from the AD Knowledge Portal hosted on Synapse: Mathys et al (2019) (Accession syn18485175, stated brain region prefrontal cortex/Brodmann area 10), Zhou et al (2020) (Accession syn21670836, stated brain region dorsolateral prefrontal cortex), Olah et al (2020) (Accession syn21438358, stated brain region dorsolateral prefrontal cortex), Cain et al (2022) (Accession syn16780177, stated brain region dorsolateral prefrontal cortex), Green et al (2023) (Accession syn31512863, stated brain region dorsolateral prefrontal cortex/Brodmann area 9), and Mathys et all (2023) (Accession syn52293417, stated brain region dorsolateral prefrontal cortex). It also included datasets from the Sequencing Read Archive (SRA): Lau et al (2020) (Accession PRJNA662923, stated brain region prefrontal cortex), Leng et al (Accession PRJNA615180, stated brain region superior frontal gyrus), Morabito et al (2021) (Accession PRJNA729525, stated brain region prefrontal cortex), and Yang et al (2022) (Accession PRJNA686798, stated brain region superior frontal cortex). From each of these repositories separate data use agreements with the Rush Alzheimer's Disease Research Center (for donors from the ROSMAP cohort) we also obtained clinical metadata and harmonized it to a standardized schema included below. The harmonization was done reproducibly, using python code to read in source files, make necessary alterations such as renaming Braak 1 to Braak I, and write out finalized files.

Reads from each snRNA-seq library were mapped to the same human reference noted above using the same cellranger pipeline as was used for SEA-AD snRNA-seq data. Mapping of nuclei to the SEA-AD cellular taxonomy was done separately from each dataset using the same iterative scVI and scANVI procedure described above to map SEA-AD nuclei from A9 to the neurotypical BRAIN initiative reference dataset.

Flagging of low quality nuclei, doublets, and donor-specific nuclei and identification of cell types not in the SEA-AD cellular taxonomy was also done identically to above except neighborhood graphs, leiden clustering, and UMAP visualizations were computed with the GPU-accelerated rapids_singlecell python package (version 0.9.2) with the drop-in replacement functions rapids_singlecell.pp.neighbors, rapids_singlecell.tl.leiden, and rapids_singlecell.tl.umap using default parameters. 3 cell types were added (all perivascular cell types from VINE-seq in Yang et al (2022), as expected), which were found in SEA-AD datasets but in levels too low to drive cluster separation. Mapping results were validated two ways: (1) the probabilities from scANVI for each supertype across each dataset and (2) a signature score computed for each supertype. The top 10 marker genes for each supertype within the SEA-AD dataset compared to all other supertypes within its cellular neighborhood (see **Extended Data Figure 9d**) were identified using the same Wilcoxon test described above. Next, we computed each supertypes' signature score for each nucleus by transforming the log-normalized expression values (i.e. natural log of UMIs per 10,000 plus 1, computed with the scanpy.pp.normalize_total and scanpy.pp.log1p functions with default parameters) for each of these marker genes to z-scores using the scanpy.pp.scale function (with default parameters) and then taking their mean (**Extended Data Figure 9c**). Closely related supertypes could have similar signature scores, but would retain the same rank order across datasets, if correctly mapped (e.g. Sst_20, Sst_22, Sst_25, Sst_23 and Sst_11 nuclei would all have a high Sst_25 signature score on average, but the order from highest to lowest should be retained across datasets). To test this, we computed the spearman correlation of each supertypes signature score across all supertypes within a cellular neighborhood between the SEA-AD dataset and every other publicly available dataset using the scipy.stats.spearmanr function (scipy version 1.8.1).

*Common metadata format/specification for every library*

- library_prep - (Required) str
- Donor ID - (Required) str
- Brain Region - (Reguired) literal 'MTG' or 'PFC'
- Method - (Required) literal 3' 10x v2, 3' 10x v3, 3' 10x v3.1, 3' 10x Multiome, or 5' 10x v1
- RIN - float
- barcode – str

*Common metadata format/specification for every donor*

- Donor ID - (Required) str
- Publication - (Required) str
- Primary Study Name - (Required) str
- Age at Death (Required) - int, float or literal '90+'
- Sex - (Required) Male or Female
- Race (choice=White) – literal 'Checked' or 'Unchecked'
- Race (choice=Black/ African American) - literal 'Checked' or 'Unchecked'
- Race (choice=Asian) - literal 'Checked' or 'Unchecked'
- Race (choice=American Indian/ Alaska Native) - literal 'Checked' or 'Unchecked'

- Race (choice=Native Hawaiian or Pacific Islander) - literal 'Checked' or 'Unchecked'
- Race (choice=Unknown or unreported) - literal 'Checked' or 'Unchecked'
- Race (choice=Other) - literal 'Checked' or 'Unchecked'
- Hispanic/Latino - literal 'Y' or 'N'
- Years of education - int or float
- APOE4 Status - (Required) literal 'Y' or 'N'
- PMI - (Required) float
- Fresh Brain Weight - float
- Brain pH - float
- Overall AD neuropathological Change - literal Not AD, Low, Intermediate, or High
- Thal - literal 'Thal 0', 'Thal 1', 'Thal 2', 'Thal 3', 'Thal 4', or 'Thal 5'
- Braak - (Required) literal 'Braak 0', 'Braak I', 'Braak II', 'Braak III', 'Braak IV', 'Braak V', or 'Braak VI'
- CERAD score - literal 'Absent', 'Sparse', 'Moderate', or 'Frequent'
- Cognitive Status - (Required) literal 'No dementia' or 'Dementia'
- Highest Lewy Body Disease - literal 'Not Identified (olfactory bulb not assessed)', 'Not Identified (olfactory bulb assessed)', 'Olfactory bulb only', 'Amygdala-predominant', 'Brainstem-predominant', 'Limbic (Transitional)', or 'Neocortical (Diffuse)'
- LATE - literal 'Unclassifiable', 'Not Identified', 'LATE Stage 1', 'LATE Stage 2', 'LATE Stage 3'
- Overall CAA Score - literal 'Not identified', 'Mild', 'Moderate', 'Severe'
- Atherosclerosis - literal 'None', 'Mild', 'Moderate', 'Severe'
- Arteriolosclerosis - literal 'None', 'Mild', 'Moderate', 'Severe'

## Methods for Spatial Transcriptomic Processing.

### Spatial transcriptomics data quality control and mapping

Resulting transcript location data and cell by gene tables were assessed for quality by comparing total transcript counts across specimens. A rectangular region was selected in each section to encompass a region spanning pia to white matter with uniform layer thickness and minimal in-plane cortical curvature. Transcript counts within these regions were summed to create a spatial transcriptomics pseudo-bulk profile. This pseudo-bulk profile was consistent with the bulk RNASeq measurements summed across 10 donors (Pearson correlation 0.69). Two sections with total transcript correlation less than 0.6 to the spatial transcriptomic pseudo-bulk were eliminated, along with two sections that measured unusually high counts of one gene (HS3ST2). Within the cortical selections, layers were annotated manually based on excitatory subclass annotations and cellular density. After these steps, selected cells from 69 sections from 27 donors formed our spatial dataset for subsequent analysis. Cells were eliminated from further analysis if they fell outside the following criteria: >15 genes detected, 30-4000 total transcripts detected, 100-4000 $um^3$ total cell volume. Cells in this dataset had a mean of 208.9 detected transcripts, and mean volume of 1273 $\mu m^3$.Cells in the spatial transcriptomics dataset were mapped to the integrated taxonomy at the supertype level by finding the supertype whose mean gene expression within the supertype best

matched to each cell. Specifically, we used a bootstrapped Pearson correlation in the map_cells_knn_bs function in the scrattch-mapping R package version 0.16.

**Spatial transcriptomics gene panel selection**

The 140 gene human cortical panel (Vizgen panel VZG167) was selected using a combination of manual and algorithmic based strategies requiring a reference single cell/nucleus RNA-seq data set from the same tissue, in this case the human MTG snRNA-seq dataset and resulting taxonomy[1]. First, an initial set of 40 high-confidence marker genes are selected through a combination of literature search and analysis of the reference data. These genes are used as input for a greedy algorithm (detailed below). Second, the reference RNA-seq data set is filtered to only include genes compatible with mFISH. Retained genes need to be 1) long enough to allow probe design (>960 base pairs); 2) expressed highly enough to be detected (FPKM >=10 in at least one cell type cluster), but not so high as to overcrowd the signal of other genes in a cell (FPKM <500 across all cell type clusters); 3) expressed with low expression in off-target cells (FPKM <50 in non-neuronal cells); and 4) differentially expressed between cell types (top 500 remaining genes by marker score, see code below). To sample each cell type more evenly, the reference data set is also filtered to include a maximum of 50 cells per cluster.

The computational step of gene selection uses a greedy algorithm to iteratively add genes to the initial set. To do this, each cell in the filtered reference data set is mapped to a cell type by taking the Pearson correlation of its expression levels with each cluster median using the initial gene set of size n, and the cluster corresponding to the maximum value is defined as the mapped cluster. The mapping distance is then defined as the average cluster distance between the mapped cluster and the originally assigned cluster for each cell. In this case a weighted cluster distance, defined as one minus the Pearson correlation between cluster medians calculated across all filtered genes, is used to penalize cases where cells are mapped to very different types, but an unweighted distance, defined as the fraction of cells that do not map to their assigned cluster, could also be used. This mapping step is repeated for every possible n+1 gene set in the filtered reference data set, and the set with minimum cluster distance is retained as the new gene set. These steps are repeated using the new get set (of size n+1) until a gene panel of the desired size is attained. Code for reproducing this gene selection strategy is available as part of the mfishtools R library (https://github.com/AllenInstitute/mfishtools).

## Methods for Gene Expression Analysis.

**Gene expression changes along CPS**

To model gene expression changes along CPS while adjusting for other covariates and pseudo-replication within donors we used a general linear mixed effects model implemented in the NEBULA R package[23] (version 1.2.0) accessed in python via the rpy2 package (version 3.5.2). We used objects with all nuclei and with nuclei divided into the first (<0.55, early) and second (>0.45, late) disease phase along CPS. Briefly, we split CPS into two phases: (1) A period where donors had normal cognition and relatively low levels of plaque and tangle pathology (but changes in other quantitative neuropathology variables) and (2) a period where donors had markedly increased levels of plaque and tangle pathology and cognitive

decline. To delineate the cutoffs for these phases, we interrogated the general additive models used to relate the number of amyloid plaques and tau tangles to CPS. Significant coefficients were first observed for splines starting at 0.4 and 0.6, depending on the variable and layer. We chose CPS=0.5 as the intermediary cut point but added a small amount of overlap to account for uncertainty precisely when the transition occurs. For each supertype, we constructed a model matrix from relevant metadata with the base model.matrix function in R with the formula Sex + Age at death + Race + 10x Chemistry + CPS + Number of genes detected after standardizing numerical values to a [0,1] interval. We randomly added single pseudocounts to 3 nuclei to features that had zero values across all nuclei within a supertype in the metadata groupings, which would have prevented the model from properly fitting coefficients (e.g. the X-chromosome gene **XIST** had zero counts across all nuclei from **male** donors, so 1 pseudocount for XIST was added to 3 random male nuclei). We then grouped raw count and model matrices with the group_cell function in NEBULA, passing the counts matrix to count, the model matrix to pred, the number of UMIs detected in each nucleus to offset, and the donor IDs as the random effect to id. To fit the model, we then ran the nebula function using the output of group_cells. We filtered genes with fewer than 0.005 counts per nucleus (as recommended) which resulted in coefficients for roughly 14,000 genes being fit in each supertype. We further restricted the results to genes with convergences equal to 1. We determined the number of significant genes from the resulting p-values in each supertype with the Benjamini-Hochberg procedure with an alpha threshold of 0.01.
We excluded 11 severely affected donors from gene expression analysis due to systematically lower quality data.

**Construction of gene dynamic space**

To identify patterns in gene expression dynamics, we constructed a matrix spanning all genes on one axis and their corresponding normalized early and late beta coefficients (divided by their standard errors) from NEBULA (see above) as well as z-scores of the mean expression (capped at a magnitude of 2) for each supertype along the other axis. Genes that were not tested in a particular supertype because of low counts per cell were assigned a beta coefficient of 0. We then computed a nearest neighbor graph across all genes using Euclidian distances with the scanpy.pp.neighbors function with use_rep set to X and n_neighbors set to 15. To visualize the resulting graph, we computed a low dimensional UMAP representation with the scanpy.ul.umap function with default parameters and computed mean normalized beta coefficient and z-score values across classes and subclasses for visualization purposes.

**Curation of gene sets**

We constructed 31 gene sets that encompass molecular processes important for neurons or implicated in AD from various databases and literature sources noted below. The gene lists are compiled in **Supplementary Table 6**.

- Electron transport chain, based on Gene Ontology (GO) 0022900
- Glycolysis, based on BIOCYC Pathway PWY66-400
- Cholesterol biosynthesis, BIOCYC Pathways PWY66-341, PWY66-3, PWY66-4
- Steroid metabolism, based on UniProt Keyword KW-0753 (reviewed only)
- Fatty acid metabolism, based on UniProt Keyword KW-0276 (reviewed only)

- Phospholipid metabolism, based on UniProt Keyword KW-1208 (reviewed only)
- Sphingolipid metabolism, based on UniProt Keyword KW-0746 (reviewed only)
- Ribosomal proteins, based on GO 0006412
- Eukaryotic initiation, elongation and termination factors, based on GO 0006412
- Transcriptional machinery, based on GO 0006351
- Ubiquitin machinery (split by Category), based on Unibet 2.0[24]
- Kinases (split by group), based on KinHub[25]
- Voltage gated ion channels, based on the Guide to Pharmacology (GtP)[26]
- Ligand gated ion channels, based on the Guide to Pharmacology (GtP)
- Nuclear hormone receptors, based on the Guide to Pharmacology (GtP)
- Transcription factors (split by Family) based on the Guide to Pharmacology (GtP)
- Genes identified by Genome Wide Association Studies (GWAS)[27]
- Cell adhesion, based on GO 0007155
- Actin-Spectrin-Spetin cytoskeleon, based on GO 0005200, 0003779, 0030507, and 0031105
- Microtubule cytoskeleton, based on GO 0005200, 0015630, 0008017
- Molecular motors used in vesicle trafficking, based on Hirokawa et al[28]
- Cargo adaptors used in vesicle trafficking, based on Hirokawa et al[28] and GO 0030705 and 0016192
- Axonal guidance cues, based on GO 0097485
- Neuropeptides, based on Smith et al[29]
- Neuropeptide receptors, based on Smith et al[29]
- Myelin components, based on Morell et al[30]
- OPC differentiation and re-myleination program, based on Nakatani et al[31], Arnett et al[32], Zhang et al[33], and Tomassy et al[34]
- Fc receptors, based on Owen et al[35]
- Major histocompatibility complex class II, based on Jones et al[36]
- Human plaque induced genes, based on Chen et al[37]
- Interferon stimulated genes, based on Schneider et al[38]

**Gene set enrichment analysis**

We employed a bootstrapping procedure to test for significant enrichment of each gene set in the early or late AD epoch along CPS, in specific cell types. Briefly, for each gene set we randomly selected the same number of genes within it 1000 times with the numpy.random.choice (version 1.22.4) function with replace set to False. For each iteration, we computed the mean early and late beta coefficients for the randomly chosen set of genes to create a background distribution for each AD epoch. We then computed a z-score for the actual gene set by computing the mean early and late beta coefficients for the genes within the set, subtracting the mean from the null distributions from them and dividing them by the standard deviation from the null distributions. We computed p-values for these z-scores using the scipy.stats.norm.cdf python function. We applied a Bonferroni correction for the number of gene lists tested and set an alpha threshold of 0.05.

**Identification of marker genes in subclasses with vulnerable and disease associated supertypes**

We used the same general linear mixed effects model (NEBULA[23]) to test for supertype specific expression within subclass. All parameters were the same for the test across CPS, except we constructed a model matrix from relevant metadata with the base model.matrix function in R using the formula Cell type + Sex + Age at death + Race + 10x Chemistry + Number of genes detected after standardizing numerical values to a [0,1] interval. Cell type was encoded as 1 for the supertype being tested and 0 for all other supertypes within the subclass.

**Gene regulatory networks**

To compute gene regulatory networks (GRNs) within non-neuronal subclasses, we used the SCENIC+[39] python package (version 1.0.1.dev3+g3741a4b). Briefly, we first created a fragment file that contained data from all SEA-AD nuclei within each non-neuronal subclass to call subclass-specific peaks using the MACS2[40] package, as recommended by SCENIC+. We constructed an ArchR[41] R object (version 1.0.1) from the fragments file and called peaks within each subclass using MACS2 implemented in the addGroupCoverages and addReproduciblePeakSet functions in ArchR with the groupBy parameter set to the subclass labels. We then exported these cell by peak matrices and created pycisTopic objects (version 1.0.3.dev18+ge563fb6). We used the pycisTopic.cistopic_class.run_cgs_models function with n_topics set to [2,4,10,16,32,48], n_cpu set to 32, and n_iter set to 500 to determine the appropriate number of topics and settled on 16 based on results from the pycisTopic.lda_models.evaluate_models function with select_model set to 16, return_model set to True and the SCENIC+ usage guide. To select candidate enhancer elements, we then binarized the topics using the pycisTopic.topic_binarization.binarize_topics function once with method set to otsu and once with method set to ntop and ntop set to 3000. We also identified differential features with the pycisTopic.diff_features.find_highly_variable_features and pycisTopic.diff_features.find_diff_features functions with default parameters after imputing and normalizing the cisTopic object with the pycisTopic.diff_features.impute_accessibility and pycisTopic.diff_features.normalize_scores functions with scale_factor set to 10**6 and 10**4, respectively. These features were used to define region sets that were associated with transcription factors with the scenicplus.wrappers.run_pycistarget function with species set to homo_sapiens, ctx_db_path set to the hg38_screen_v10_clust.regions_vs_motifs.rankings.feather file obtained from the SCENIC+ guide, dem_db_path set to the hg38_screen_v10_clust.regions_vs_motifs.scores.feather file also obtained from the SCENIC+ guide, path_to_motif_annotations set to the hg38_screen_v10_clust.regions_vs_motifs.scores.feather file also obtained from the SCENIC+ guide, run_without_promoters set to True, n_cpu set to 32, and the annotation_version set to v10nr_clust. We then passed the final pycisTopic object along with the snRNA-seq data for the non-neuronal subclasses to SCENIC+ with the scenicplus.scenicplus_class.create_SCENICPLUS_object function with the key_to_group_by set to the nuclei subclasses and nr_cells_per_metacells set to 5. We then identified GRNs with the scenicplus.wrappers.run_scenicplus function with variable set to the subclass labels, species set to hsapiens, assembly set to hg38, tf_file set to the utoronto_human_tfs_v_1.01.txt file obtained from the University of Toronto Human Transcription Factor database, upstream set to [1000, 150000], downstream set to [1000, 150000], calculate_TF_eGRN_correlation set to True, calculate_TF_eGRN_correlation set to False, and n_cpu set to 32. Finally, only gene regulatory networks with a rho value for TF_cistrome_correlation greater than 0.7 or less than -0.7 were retained, per the SCENIC+ guidelines. We then identified transcription factors within the GRNs that had both subclass-

specific expression (z-score mean gene expression greater than 2) and increased in the early AD epoch (mean NEBULA early beta coefficient greater than 1) and identified predicted downstream target genes that were common across all of them.

## References


1. Hodge, R. D. *et al.* Conserved cell types with divergent features in human versus mouse cortex. *Nature* **573**, 61–68 (2019).
2. Huang, G., Liu, Z., van der Maaten, L. & Weinberger, K. Q. Densely Connected Convolutional Networks. (2016) doi:10.48550/ARXIV.1608.06993.
3. Wolf, F. A., Angerer, P. & Theis, F. J. SCANPY: large-scale single-cell gene expression data analysis. *Genome Biol* **19**, 15 (2018).
4. Mukherjee, S. *et al.* Cognitive domain harmonization and cocalibration in studies of older adults. *Neuropsychology* **37**, 409–423 (2023).
5. Vorontsov, I. E. *et al.* HOCOMOCO in 2024: a rebuild of the curated collection of binding models for human and mouse transcription factors. *Nucleic Acids Research* **52**, D154–D163 (2024).
6. Bailey, T. L., Johnson, J., Grant, C. E. & Noble, W. S. The MEME Suite. *Nucleic Acids Res* **43**, W39–W49 (2015).
7. Lopez, R., Regier, J., Cole, M. B., Jordan, M. I. & Yosef, N. Deep generative modeling for single-cell transcriptomics. *Nat Methods* **15**, 1053–1058 (2018).
8. Xu, C. *et al.* Probabilistic harmonization and annotation of single-cell transcriptomics data with deep generative models. *Mol Syst Biol* **17**, (2021).
9. Jorstad, N. L. *et al.* Comparative transcriptomics reveals human-specific cortical features. *Science* **382**, eade9516 (2023).
10. Jorstad, N. L. *et al.* Transcriptomic cytoarchitecture reveals principles of human neocortex organization. *Science* **382**, eadf6812 (2023).

11. Gabitto, M. I. *et al.* Characterizing chromatin landscape from aggregate and single-cell genomic assays using flexible duration modeling. *Nat Commun* **11**, 747 (2020).

12. Ashuach, T., Gabitto, M. I., Jordan, M. I. & Yosef, N. MultiVI: deep generative model for the integration of multi-modal data. *bioRxiv* 2021.08.20.457057 (2021) doi:10.1101/2021.08.20.457057.

13. Mathys, H. *et al.* Single-cell transcriptomic analysis of Alzheimer's disease. *Nature* **570**, 332–337 (2019).

14. Lau, S.-F., Cao, H., Fu, A. K. Y. & Ip, N. Y. Single-nucleus transcriptome analysis reveals dysregulation of angiogenic endothelial cells and neuroprotective glia in Alzheimer's disease. *Proc. Natl. Acad. Sci. U.S.A.* **117**, 25800–25809 (2020).

15. Olah, M. *et al.* Single cell RNA sequencing of human microglia uncovers a subset associated with Alzheimer's disease. *Nat Commun* **11**, 6129 (2020).

16. Zhou, Y. *et al.* Human and mouse single-nucleus transcriptomics reveal TREM2-dependent and TREM2-independent cellular responses in Alzheimer's disease. *Nature Medicine* **26**, 131–142 (2020).

17. Leng, K. *et al.* Molecular characterization of selectively vulnerable neurons in Alzheimer's disease. *Nature Neuroscience* **24**, 276–287 (2021).

18. Morabito, S. *et al.* Single-nucleus chromatin accessibility and transcriptomic characterization of Alzheimer's disease. *Nature Genetics* **53**, 1143–1155 (2021).

19. Yang, A. C. *et al.* A human brain vascular atlas reveals diverse mediators of Alzheimer's risk. *Nature* **603**, 885–892 (2022).

20. Cain, A. *et al.* Multicellular communities are perturbed in the aging human brain and Alzheimer's disease. *Nat Neurosci* **26**, 1267–1280 (2023).

21. Green, G. S. *et al. Cellular Dynamics across Aged Human Brains Uncover a Multicellular Cascade Leading to Alzheimer's Disease*. http://biorxiv.org/lookup/doi/10.1101/2023.03.07.531493 (2023) doi:10.1101/2023.03.07.531493.

22. Mathys, H. *et al.* Single-cell atlas reveals correlates of high cognitive function, dementia, and resilience to Alzheimer's disease pathology. *Cell* **186**, 4365-4385.e27 (2023).

23. He, L. *et al.* NEBULA is a fast negative binomial mixed model for differential or co-expression analysis of large-scale multi-subject single-cell data. *Communications Biology* **4**, 629 (2021).

24. Li, Z. *et al.* UbiNet 2.0: a verified, classified, annotated and updated database of E3 ubiquitin ligase–substrate interactions. *Database* **2021**, baab010 (2021).

25. Eid, S., Turk, S., Volkamer, A., Rippmann, F. & Fulle, S. KinMap: a web-based tool for interactive navigation through human kinome data. *BMC Bioinformatics* **18**, 16 (2017).

26. Harding, S. D. *et al.* The IUPHAR/BPS Guide to PHARMACOLOGY in 2024. *Nucleic Acids Research* **52**, D1438–D1449 (2024).

27. Bellenguez, line *et al.* New insights into the genetic etiology of Alzheimer's disease and related dementias. *Nature Genetics 2022* 1–25 (2022) doi:10.1038/s41588-022-01024-z.

28. Hirokawa, N., Niwa, S. & Tanaka, Y. Molecular Motors in Neurons: Transport Mechanisms and Roles in Brain Function, Development, and Disease. *Neuron* **68**, 610–638 (2010).

29. Smith, S. J. *et al.* Single-cell transcriptomic evidence for dense intracortical neuropeptide networks. *eLife* **8**, e47889 (2019).

30. Pierre Morell & Richard H Quarles. Characteristic Composition of Myelin. in *Basic neurochemistry: molecular, cellular, and medical aspects* (ed. Siegel, G. J.) (Lippincott Williams & Wilkins, Philadelphia, 1999).

31. Nakatani, H. *et al.* Ascl1/Mash1 promotes brain oligodendrogenesis during myelination and remyelination. *J Neurosci* **33**, 9752–9768 (2013).

32. Arnett, H. A. *et al.* bHLH Transcription Factor Olig1 Is Required to Repair Demyelinated Lesions in the CNS. *Science* **306**, 2111–2115 (2004).

33. Zhang, Y. *et al.* Notch1 signaling plays a role in regulating precursor differentiation during CNS remyelination. *Proc Natl Acad Sci U S A* **106**, 19162–19167 (2009).

34. Tomassy, G. S., Dershowitz, L. B. & Arlotta, P. Diversity Matters: A Revised Guide to Myelination. *Trends in Cell Biology* **26**, 135–147 (2016).

35. Owen, J., Punt, J., Stranford, S. A. & Kuby, J. *Kuby Immunology*. (Macmillan Higher Education, Basingstoke, 2013).

36. Jones, E. Y., Fugger, L., Strominger, J. L. & Siebold, C. MHC class II proteins and disease: a structural perspective. *Nat Rev Immunol* **6**, 271–282 (2006).

37. Chen, W. T. *et al.* Spatial Transcriptomics and In Situ Sequencing to Study Alzheimer's Disease. *Cell* **182**, 976-991.e19 (2020).

38. Schneider, W. M., Chevillotte, M. D. & Rice, C. M. Interferon-stimulated genes: a complex web of host defenses. *Annu Rev Immunol* **32**, 513–545 (2014).

39. Bravo González-Blas, C. *et al.* SCENIC+: single-cell multiomic inference of enhancers and gene regulatory networks. *Nat Methods* **20**, 1355–1367 (2023).

40. Gaspar, J. M. *Improved Peak-Calling with MACS2*. http://biorxiv.org/lookup/doi/10.1101/496521 (2018) doi:10.1101/496521.

41. Granja, J. M. *et al.* ArchR is a scalable software package for integrative single-cell chromatin accessibility analysis. *Nat Genet* **53**, 403–411 (2021).

**Supplementary Figure 1.** Representative FANS gating strategy showing gating for NeuN-positive and -negative singlet nuclei

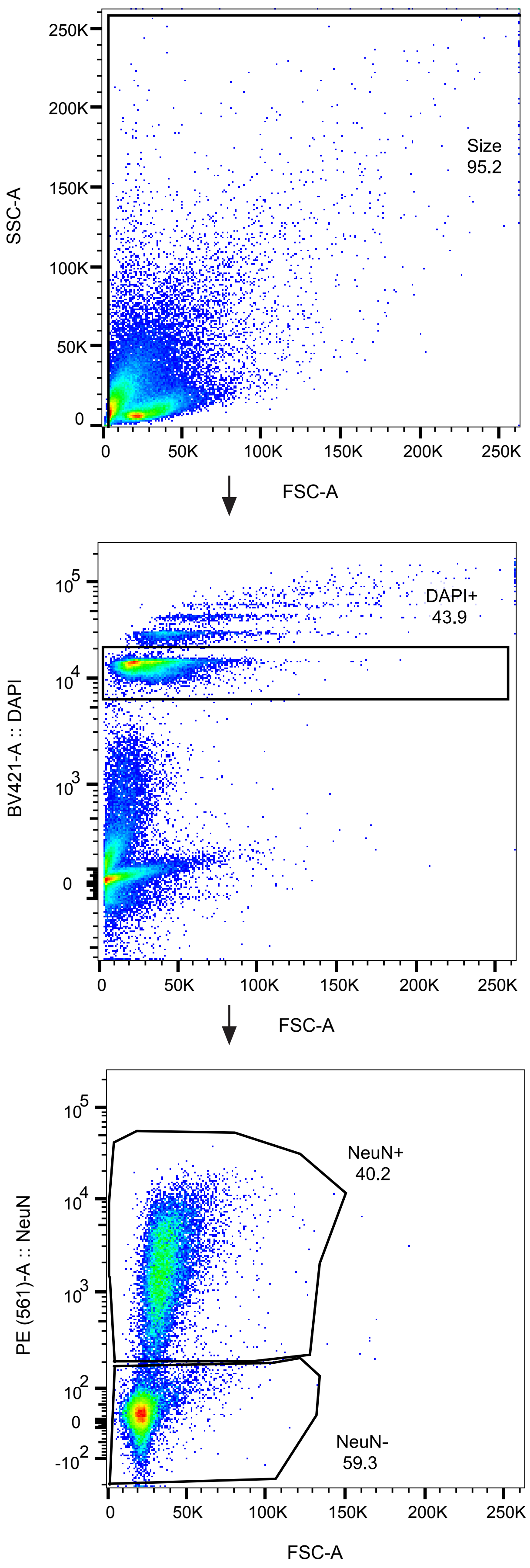

Supplement: Supplementary file 1 — Supplementary Note and Supplementary Fig. 1. [file 41593_2024_1774_MOESM1_ESM.pdf]
