# Supplementary material for: Integrated multimodal cell atlas of Alzheimer’s disease
Source: Nat Neurosci. 2024 Oct 14;27(12):2366–83. doi: 10.1038/s41593-024-01774-5 (PMC11614693; doi:10.1038/s41593-024-01774-5)
Supplement: Supplementary file 2 — Reporting Summary [file 41593_2024_1774_MOESM2_ESM.pdf]

nature portfolio

Corresponding author(s): Michael Hawrylycz, C. Dirk Keene, Ed S. Lein

Last updated by author(s): Aug 19, 2024

Reporting Summary

Nature Portfolio wishes to improve the reproducibility of the work that we publish. This form provides structure for consistency and transparency in reporting. For further information on Nature Portfolio policies, see our Editorial Policies and the Editorial Policy Checklist.

Statistics

For all statistical analyses, confirm that the following items are present in the figure legend, table legend, main text, or Methods section.

n/a	Confirmed	
☐	☒	The exact sample size (*n*) for each experimental group/condition, given as a discrete number and unit of measurement
☐	☒	A statement on whether measurements were taken from distinct samples or whether the same sample was measured repeatedly
☐	☒	The statistical test(s) used AND whether they are one- or two-sided<br>*Only common tests should be described solely by name; describe more complex techniques in the Methods section.*
☐	☒	A description of all covariates tested
☐	☒	A description of any assumptions or corrections, such as tests of normality and adjustment for multiple comparisons
☐	☒	A full description of the statistical parameters including central tendency (e.g. means) or other basic estimates (e.g. regression coefficient) AND variation (e.g. standard deviation) or associated estimates of uncertainty (e.g. confidence intervals)
☐	☒	For null hypothesis testing, the test statistic (e.g. *F*, *t*, *r*) with confidence intervals, effect sizes, degrees of freedom and *P* value noted<br>*Give P values as exact values whenever suitable.*
☐	☒	For Bayesian analysis, information on the choice of priors and Markov chain Monte Carlo settings
☐	☒	For hierarchical and complex designs, identification of the appropriate level for tests and full reporting of outcomes
☐	☒	Estimates of effect sizes (e.g. Cohen's *d*, Pearson's *r*), indicating how they were calculated

Our web collection on statistics for biologists contains articles on many of the points above.

Software and code

Policy information about availability of computer code

Data collection	Aperio AT2 digital scanner software (102.0.7.5) - Whole slide imaging<br>BD Diva software (8.0) - Fluorescence activated nuclei sorting<br>Fragment Analyzer (1.2.0.11) - Quantification of cDNA library fragment sizes<br>Illumina NovaSeq control software - DNA library sequencing<br>Vizgen MERSCOPE control software - Obtaining spatial transcriptomic data
Data analysis	HALO (3.4.2986), cellranger (6.0), cellranger-arc (2.0), vizgen-postprocessing (alpha release), python (3.9.7), numpy (1.22.0), scipy (1.8.1), seaborn (0.11.2), scikit-learn (1.1.1) scanpy (versions 1.8.1 and 1.9.1), pandas (1.4.2), anndata (0.7.8), sccoda (0.1.7), scvi-tools (0.14.6, includes scVI, MultiVI, and scANVI), statsmodels (0.13.2), pytorch (1.10.0), R (4.1.0), nebula (1.2.0), rpy2 (3.5.2), cellxgene (1.1.0).

For manuscripts utilizing custom algorithms or software that are central to the research but not yet described in published literature, software must be made available to editors and reviewers. We strongly encourage code deposition in a community repository (e.g. GitHub). See the Nature Portfolio guidelines for submitting code & software for further information.

Data

Policy information about availability of data

All manuscripts must include a data availability statement. This statement should provide the following information, where applicable:

- Accession codes, unique identifiers, or web links for publicly available datasets
- A description of any restrictions on data availability
- For clinical datasets or third party data, please ensure that the statement adheres to our policy

FASTQs containing sequencing data from snRNA-seq, snATAC-seq, and snMultiome assays are available through controlled access at Sage Bionetworks (accession: syn26223298). Nuclei by gene matrices with counts and normalized expression values from snRNA-seq and snMultiome assays are available through the Open Data Registry on AWS as AnnData objects (h5ad), and viewable on the cellxgene platform. Nuclei by peak matrices for the snATAC-seq data (with peaks called across all nuclei) and cell by gene matrices containing spatial coordinates from MERFISH data are also available on the Open Data Registry on AWS as AnnData objects. Donor, library, and cell-level metadata is available in these objects and also on SEA-AD.org. Raw images from the quantitative neuropathology data are available on the Open Data Registry on AWS and the variables derived from HALO on SEA-AD.org. The collection of scripts used to annotate the SEA-AD and publicly available datasets, perform all analyses, and build each figure are on the Allen Institute GitHub page: https://github.com/AllenInstitute/SEA-AD_2024.

We obtained raw sequencing reads from 10 publicly available datasets that performed single cell or single nucleus RNA-seq on or near the PFC of human cohorts that included sporadic AD donors. These included datasets from the AD Knowledge Portal hosted on Synapse: Mathys et al (2019) (Accession syn18485175, stated brain region prefrontal cortex/Brodmann area 10), Zhou et al (2020) (Accession syn21670836, stated brain region dorsolateral prefrontal cortex), Olah et al (2020) (Accession syn21438358, stated brain region dorsolateral prefrontal cortex), Cain et al (2022) (Accession syn16780177, stated brain region dorsolateral prefrontal cortex), Green et al (2023) (Accession syn31512863, stated brain region dorsolateral prefrontal cortex/Brodmann area 9), and Mathys et all (2023) (Accession syn52293417, stated brain region dorsolateral prefrontal cortex). It also included datasets from the Sequencing Read Archive (SRA): Lau et al (2020) (Accession PRJNA662923, stated brain region prefrontal cortex), Leng et al (Accession PRJNA615180, stated brain region superior frontal gyrus), Morabito et al (2021) (Accession PRJNA729525, stated brain region prefrontal cortex), and Yang et al (2022) (Accession PRJNA686798, stated brain region superior frontal cortex). From each of these repositories separate data use agreements with the Rush Alzheimer's Disease Research Center (for donors from the ROSMAP cohort) we also obtained clinical metadata and harmonized it to a standardized schema included below.

Research involving human participants, their data, or biological material

Policy information about studies with human participants or human data. See also policy information about sex, gender (identity/presentation), and sexual orientation and race, ethnicity and racism.

Reporting on sex and gender	We include sex as a covariate in all our models and report it for each donor in Supplementary Table 1. We discuss our cohorts bias for female donors in the manuscript and how that is expected from Alzheimer's disease prevalence.
Reporting on race, ethnicity, or other socially relevant groupings	We include race as a covariate in all our models and report it for each donor in Supplementary Table 1. Our cohort is almost entirely of European descent, so we use White versus Non-white as a binary categorization in our models.
Population characteristics	We include age at death as a covariate in all our models and report it for each donor in Supplementary Table 1.
Recruitment	Brain specimens were obtained from the Adult Changes in Thought (ACT) Study and the University of Washington Alzheimer's Disease Research Center (ADRC). The study cohort includes all ACT precision rapid autopsies and UW ADRC Clinical Core autopsies, with exclusion of those with a diagnosis of frontotemporal dementia (FTD), frontotemporal lobar degeneration (FTLD), Down's syndrome, amyotrophic lateral sclerosis (ALS) or other confounding degenerative disorder (not including Lewy Body Disease or uVBI). The cohort also excludes individuals that tested positive for COVID-19. The cohort represents the full spectrum of Alzheimer's disease severity.<br>The Adult Changes in Thought (ACT) study is a community cohort study of older adults from Kaiser Permanente Washington (KPW), formerly Group Health, in partnership with the University of Washington (UW). The ACT study seeks to understand the various conditions and life-long medical history that can contribute to neurodegeneration and dementia and has been continuously running since 1994, making it the longest running study of its kind. In 2005, ACT began continuous enrollment with the same methods to replace attrition from dementia, dropout, and death, ensuring a consistent cohort of ≥2,000 at risk for dementia. Total enrollment is nearing 6,000, with over 1,000 incident dementia cases; more than 900 have had autopsies to date with an average rate of approximately 45-55 per year. The study completeness of the follow up index is between 95 to 97%. Subjects are invited to enroll at age 65 by random selection from the patient population of KPW Seattle and undergo bi-annual study visits for physical and mental examinations. In addition to this study data, the full medical record is available for research through KPW. Approximately 25% of ACT autopsies are from people with no MCI or dementia at their last evaluation; roughly 30% meet criteria for MCI, and roughly 45% meet criteria for dementia. Thus, the ACT study provides an outstanding cohort of well-characterized subjects with a range of mixed pathologies including many controls appropriate for studies proposed for this study. Approximately 30% of the ACT cohort consents to brain donation upon death, and tissue collection is coordinated by the UW Biorepository and Integrated Neuropathology (BRaIN) lab, which preserves brain tissue for fixed, frozen, and fresh preparations, as well as performing a full post-mortem neuropathological examination and diagnosis by certified neuropathologists using the NIA-AA criteria.<br>The University of Washington Alzheimer's Disease Research Center (ADRC) has been continuously funded by NIH since 1984. It is part of a nationwide network of Alzheimer's disease research resource centers funded through the NIH's National Institute on Aging (NIA) and contributes uniquely to this premier program through its vision of precision medicine for AD: comprehensive investigation of an individual's risk, surveillance with accurate and early detection of pathophysiologic processes while still preclinical, and interventions tailored to an individual's molecular drivers of disease. Patients enrolled in the UW ADRC Clinical Core undergo annual study visits, including mental and physical exams, donations of biospecimens including blood and serum, and family interviews. The UW ADRC is advancing understanding of clinical and mechanistic

heterogeneity of Alzheimer's disease, developing pre-clinical biomarkers, and, in close collaboration with the ACT study, contributing to the state of the art in neuropathological description of the disease. For subjects who consent to brain donation, tissue is also collected by the UW BRaIN lab, and is preserved and treated with the same full post-mortem diagnosis and neuropathological work up as described above.
Human brain tissue was collected at rapid autopsy (postmortem interval <12 hours, mean close to 6.5, Extended Data Fig. 1a). One hemisphere (randomly selected) was embedded in alginate for uniform coronal slicing (4mm), with alternating slabs fixed in 10% neutral buffered formalin or frozen in a dry ice isopentane slurry. Superior and Middle Temporal Gyrus (MTG) was sampled from fixed slabs and subjected to standard processing, embedding in paraffin (Extended Data Fig. 1b).

Ethics oversight

In compliance with all ethical standards, informed consent for research brain donation was obtained according to protocols approved by the UW and KPWHRI Institutional Review Boards. ACT participants receive compensation for parking/transportation and an incentive of $50 after completing each study visit. Work at the Allen Institute received a regulatory determination of Not Human Subjects research.

Note that full information on the approval of the study protocol must also be provided in the manuscript.

Field-specific reporting

Please select the one below that is the best fit for your research. If you are not sure, read the appropriate sections before making your selection.

- [x] Life sciences
- [ ] Behavioural & social sciences
- [ ] Ecological, evolutionary & environmental sciences

For a reference copy of the document with all sections, see nature.com/documents/nr-reporting-summary-flat.pdf

Life sciences study design

All studies must disclose on these points even when the disclosure is negative.

Sample size

Sample size was not predetermined, we generated quantitative neuropathology single nucleus -omics datasets on all donors with available tissues that were obtained with updated post-mortem processing procedures that met our co-morbidity exclusion criteria at the start of the study.

Data exclusions

We excluded single nucleus -omics data generated from 2 donors because of poor pre-sequencing quality control metrics (low RIN and brain pH). All other data was included. We describe in Methods how we identify and exclude low quality nuclei from otherwise high quality donors.

Replication

All experiments were performed on the same cohort of donors. IHC stains were performed across whole slide images and entire anatomical regions were quantified instead of multiple fields of view within the same region. We generated two single nucleus RNAseq libraries for every donor, but only 1 library for single nucleus ATACseq and single nucleus Multiome (in a subset of donors). We profiled between 2 and 4 whole sections with MERFISH for spatial transcriptomics in a subset of donors. All data (including replicates) generated were included in the manuscript except as explicitly noted (e.g. exclusion of data from 2 donors due to low quality pre-sequencing metrics).

Randomization

There was no randomization used in cohort selection. It is not relevant for this study.

Blinding

Human specimens were assigned a unique numerical code. Researchers had access to demographic and clinical information about donors as well as the unique numerical code assigned to each donor.

Reporting for specific materials, systems and methods

We require information from authors about some types of materials, experimental systems and methods used in many studies. Here, indicate whether each material, system or method listed is relevant to your study. If you are not sure if a list item applies to your research, read the appropriate section before selecting a response.

Materials & experimental systems

n/a	Involved in the study	
[ ]	[x]	Antibodies
[x]	[ ]	Eukaryotic cell lines
[x]	[ ]	Palaeontology and archaeology
[x]	[ ]	Animals and other organisms
[x]	[ ]	Clinical data
[x]	[ ]	Dual use research of concern
[x]	[ ]	Plants

Methods

n/a	Involved in the study	
[x]	[ ]	ChIP-seq
[ ]	[x]	Flow cytometry
[x]	[ ]	MRI-based neuroimaging

Antibodies

Antibodies used

Antibodies targeted to the following antigens were used for IHC: NeuN (1:500, A60, Mouse, Millipore MAB377), pTDP43 (1:1000, Ser409/Ser410, ID3, Rat, Biolegend 829901), Beta Amyloid (1:1000, 6e10, Mouse, Biolegend 803003), Alpha-Synuclein (1:200, LB509,

Mouse, Invitrogen 180215), GFAP (1:1000, Rabbit, DAKO Z033401-2), IBA1 (1:1000, Rabbit, Wako 019-19741), and PHF-TAU (1:1000, AT8, Mouse, Thermofisher MN1020), Rat IgG (Manufacturer's proprietary dilution, Goat, Vector Laboratories MP-7444).

Mouse IgG and Rabbit IgG were detected with proprietary probes using MACH3-Mouse (M3M530 and M3M532) and MACH3-Rabbit (M3R531 and M3R533) from BioCare medical.

Antibodies targeted to the following antigens used for flow cytometry: NeuN (P1:250, E conjugated, Mouse, EMD Millipore, Milli-Mark, clone A60).

Validation

Antibodies were validated by their manufacturers as described below:
1. NeuN: Evaluated by IHC on rat cerebellum and by flow cytometry using U251 cells
2. pTDP43: Evaluated by WB on rat brain lysate
3. Beta amyloid: Evaluated by WB on 50ng of the recombinant human APP751 protein
4. Alpha-Synuclein: Evaluated by IHC staining of human Parkinson's disease tissue
5. GFAP: The antibody has been solid-phase absorbed with human and cow serum proteins. In crossed immunoelectrophoresis using 50 µL antibody per cm2 gel area, no reaction with 2 µL human plasma and 2 µL cow serum is observed. The antibody shows one distinct precipitate (GFAP) with cow brain extract
6. IBA1: Evaluated by IHC in mouse cerebellum tissue.
7. PHF-TAU: Evaulated by ICC/IF on SH-SY5Y cells.
8. Rat IgG: Evaluated by IHC to detect a rat anti-CD45 antibody in human tonsil tissue.

Plants

Seed stocks

Report on the source of all seed stocks or other plant material used. If applicable, state the seed stock centre and catalogue number. If plant specimens were collected from the field, describe the collection location, date and sampling procedures.

Novel plant genotypes

Describe the methods by which all novel plant genotypes were produced. This includes those generated by transgenic approaches, gene editing, chemical/radiation-based mutagenesis and hybridization. For transgenic lines, describe the transformation method, the number of independent lines analyzed and the generation upon which experiments were performed. For gene-edited lines, describe the editor used, the endogenous sequence targeted for editing, the targeting guide RNA sequence (if applicable) and how the editor was applied.

Authentication

Describe any authentication procedures for each seed stock used or novel genotype generated. Describe any experiments used to assess the effect of a mutation and, where applicable, how potential secondary effects (e.g. second site T-DNA insertions, mosiacism, off-target gene editing) were examined.

Flow Cytometry

Plots

Confirm that:

☒ The axis labels state the marker and fluorochrome used (e.g. CD4-FITC).

☒ The axis scales are clearly visible. Include numbers along axes only for bottom left plot of group (a 'group' is an analysis of identical markers).

☒ All plots are contour plots with outliers or pseudocolor plots.

☒ A numerical value for number of cells or percentage (with statistics) is provided.

Methodology

Sample preparation

To remove a specific region of interest from frozen 4mm thick brain slabs for downstream nuclear sequencing applications, tissue slabs were removed from storage at –80C, briefly transferred to a –20C freezer to prevent tissue shattering during dissection, and then handled on a custom cold table maintained –20C during dissection. Dissections were performed using dry ice cooled razor blades or scalpels to prevent warming of tissues. Dissected tissue samples were transferred to vacuum seal bags, sealed, and stored at -80C until the time of use. Single nucleus suspensions were generated using a previously described standard procedure (https://www.protocols.io/view/isolation-of-nuclei-from-adult-human-brain-tissue-ewov149p7vr2/v2). Briefly, after tissue homogenization, isolated nuclei were stained with a primary antibody against NeuN (FCMAB317PE, Millipore-Sigma) to label neuronal nuclei. Nuclei samples were analyzed using a BD FACS Aria flow cytometer and nuclei were sorted using a standard gating strategy to exclude multiplets17. A defined mixture of neuronal (70%) and non-neuronal (30%) nuclei was sorted for each sample.

Instrument

BD FACS Aria flow cytometer

Software

BD Diva software (8.0)

Cell population abundance

A defined mixture of neuronal/NeuN-positive (70%) and non-neuronal/NeuN-negative (30%) nuclei were sorted for each donor.

Gating strategy

Nuclei were first gated based on size (forward scatter area, FSC-A) and granularity (side scatter area, SSC-A). Nuclei were then gated on DAPI fluorescence, followed by gates to exclude doublets and aggregates (FSC-single cells, SSC-single cells). Lastly, nuclei were gated based on NeuN PE signal (NeuN-PE-A) to differentiate neuronal (NeuN+) and non-neuronal (NeuN-) nuclei.

☒ Tick this box to confirm that a figure exemplifying the gating strategy is provided in the Supplementary Information.
